# An Expeditious Approach towards the Synthesis and Application of Water-Soluble and Photostable Fluorogenic Chromones for DNA Detection

**DOI:** 10.3390/molecules27072267

**Published:** 2022-03-31

**Authors:** Steve Vincent, Suman Mallick, Guillaume Barnoin, Hoang-Ngoan Le, Benoît Y. Michel, Alain Burger

**Affiliations:** Institut de Chimie de Nice, CNRS UMR 7272, Université Côte d’Azur, Parc Valrose, CEDEX 2, 06108 Nice, France; steve.vincent@univ-cotedazur.fr (S.V.); suman22msc@gmail.com (S.M.); guillaume.barnoin@univ-cotedazur.fr (G.B.); lhngoan2109@gmail.com (H.-N.L.)

**Keywords:** fluorescent dyes, chromones, post-synthetic ODN labeling, hybridization, fluorogenic sensing, multistep synthesis

## Abstract

The intensive research for hybridization probes based on organic molecules with fluorogenic properties is currently attracting particular attention due to their potential to efficiently recognize different DNA conformations and the local environment. However, most established organic chromophores do not meet the requirements of this task, as they do not exhibit good brightness in aqueous buffer media, develop aggregation and/or are not easily conjugated to oligodeoxynucleotides (ODNs) while keeping their photophysics intact. Herein, an important modification strategy was employed for a well-known fluorophore, 2-(4-(diethylamino)phenyl)-3-hydroxychromone (**dEAF**). Although this push–pull dye absorbs intensively in the visible range and shows emission with large Stokes shifts in all organic solvents, it is strongly quenched in water. This Achilles’ heel prompted us to implement a new strategy to obtain a series of dyes that retain all the photophysical features of **dEAF** in water, conjugate readily with oligonucleotides, and furthermore demonstrate sensitivity to hydration, thus paving the way for a high-performance fluorogenic DNA hybridization probe.

## 1. Introduction

The use of hybridization probes composed of organic fluorophores attached to single strands (ss) of nucleic acids has found broad applications in nanotechnology, diagnostics, biology, and medicinal chemistry [1,2,3]. Among organic labels, fluorescence reporters that “turn on” their emission (fluorogenic dyes) in response to interactions with their target are very attractive [4,5,6]. Ideally, this technique should exclude the double labeling required for quenching, energy transfer or excimer formation. This preference can be justified by the fact that the production of doubly labeled nucleic acid probes is associated with synthesis and purification issues, which also results in a higher production cost. For instance, the main source of error in detection methods based on Förster resonance energy transfer (FRET) is due to the absence of one of the partners of the FRET pair, which leads to false results [7]. The use of a unique marker should limit these complications and simplify the implementation of the technology. To this end, upon interactions with the target, the single fluorescent reporter should generate a novel fluorescence signal that can be recorded and interpreted (e.g., through a change in intensity, anisotropy, lifetime, or color). In addition, these tools should provide more convenient staining protocols as they should not require additional washing of excess reagents. This feature is particularly attractive for diagnostic and imaging applications. Although amplification of signal change is an important criterion for biological applications, other desired properties include absorption in the visible range, high brightness, large Stokes shifts, and significant photostability to limit photobleaching. However, fulfilling all these requirements presents a challenge. Several groups have taken advantage of cyanine-derived dyes whose fluorescence emission is sensitive to viscosity [8,9]. The ability of these fluorophores to increase their signal intensity in rigid media has been used in the construction of forced-intercalation (FIT) probes [10]. FIT probes were first designed as PNA (peptide nucleic acid) or DNA containing a single cyanine (e.g., thiazole orange, TO). TO was covalently introduced as a nucleobase substitute. In their single-stranded form, probes incorporating TO exhibit low fluorescence. The formation of the probe–target duplex forces the intercalation of TO between the vicinal flat aromatic nucleobases and lights up its emission (10-fold increase in fluorescence). The performance of the probe can be further improved either by introducing modified nucleotides around it (gapmer) [11], by coupling a second partner for FRET or contact quenching [12,13]. 

**Scheme 1:**
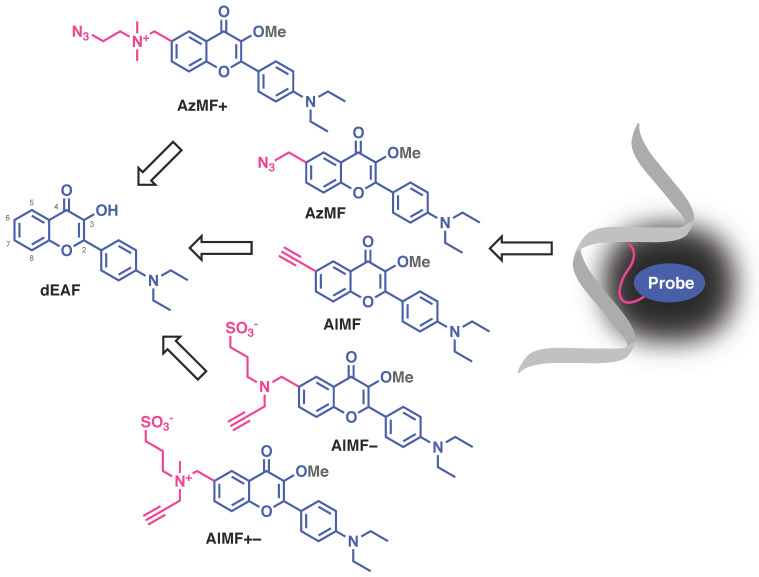
Chemical structures of the parent chromone (**dEAF**) as well as the azido (**AzMF**) and alkynyl (**AlMF**) derivatives developed for click-type post-synthetic DNA labeling. Systematic flavonoid numbering was displayed on the original core.

In our project, we sought to exploit another option to turn on the fluorescence signal and for this, we propose to harness the peculiar properties of the known 2-(4-(diethylamino)phenyl)-3-hydroxychromone (**dEAF**, Scheme 1) [14,15]. This fluorophore belongs to the family of push–pull dyes demonstrating a Donor-*π*-Acceptor (D-*π*-A) relationship. The fluorophore **dEAF** incorporates in its structure a strong donor substituent—the diethylamino group—electronically coupled to the carbonyl acceptor group. It absorbs intensively in the visible range, exhibits large Stokes shifts in polar solvents, and is emissive in all screened solvents except water where it is strongly quenched. Consequently, shielding the dye from bulk water results in a turn-on of its emission (fluorogenicity), and thus a substantial increase of the signal-to-noise ratio (SNR). This fluorogenic behavior is expected when the labeled DNA single strand anneals to its target, as the environment of the formed duplex is hydrophobic on the inside and its grooves are less hydrated than the bulk water [16,17].

**Figure 1:**
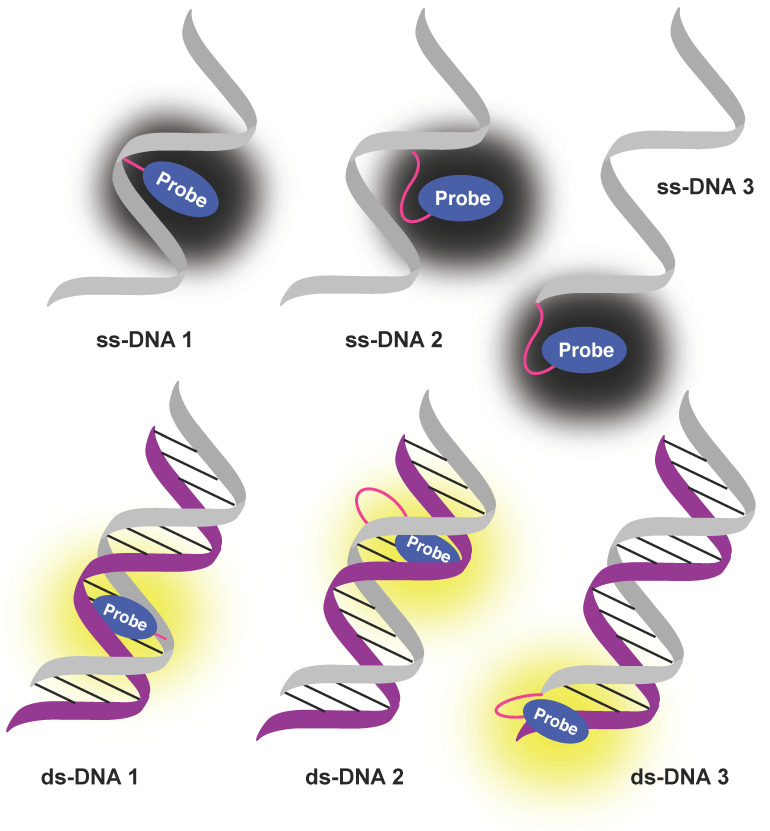
Schematic representation of the three ways to fluorescently label ODNs (**ss-DNA 1–3**) and turn on their emission upon hybridization (**ds-DNA 1–3**).

Our objective was to synthesize labeled oligodeoxynucleotides (ODNs) with turn-on emission after annealing with their complementary strand. As a convenient and straightforward access, we selected an ODN labeling through a post-synthetic CuAAC coupling. For this purpose, we had to synthesize the corresponding azido and ethynyl derivatives of the reference 3-hydroxychromone **dEAF** (Scheme 1). Position 6 of the chromone scaffold was preferred because chemical modification at this location should have minimal impact on the photophysics of the dye since it is not conjugated with the 4-ketone. The methoxy group was preferred over the hydroxyl group to radically improve the photostability of the chromone [18]. Three labeling methods were considered to allow for various modes of interaction of the dye with the double-stranded (ds) DNA (Figure 1). In **ss-DNA 1**, the fluorophore will substitute for a natural base and be positioned at the central position of the strand. After hybridization, it will be forced to intercalate (**ds-DNA 1**). For **ss-DNA 2–3**, the dye will be connected to a central or terminal position via a flexible tether. In the case of **ds-DNA 2–3**, the fluorescent reporter will be free to interact with the duplex either by intercalation/*π*-stacking or by groove binding. For our research program, we considered all options (1,2 and 3). 

Herein, we report the synthesis and application of the critically modified **dEAF** derivatives with a significant increase in the water solubility and photostability while keeping intact, the rest of the photophysics. These modified dyes were also designed to be rapidly conjugated to ODNs using standard CuAAC click-chemistry protocol [19,20]. All synthesized probes are environment-sensitive and exhibit typical fluorogenic features upon hybridization. We investigated the solvent-dependent photophysical properties of the fluorophores using steady-state spectroscopic techniques before moving on to applications. Next, we adopted a post-synthetic approach to tag with them, a variety of ss-ODNs and explored their potential to sense the hybridization process upon interaction with the complementary sequence using UV–Vis and fluorescence spectroscopies.

## 2. Results and Discussion

### 2.1. Synthesis

The 3-Hydoxychromones (3HCs) are very popular in the photophysics community for their unique ability to display environmentally sensitive excited-state intramolecular proton transfer (ESIPT), their ease of preparation, and their relatively high brightness considering their modest size. This atypical photophysical signature arises from the presence of a hydroxyl group vicinal to the ketone. However, this OH group reacts rapidly by photochemistry when irradiated by a high intensity light source (usually the laser required for bioimaging related studies), which induces a low photostability. To counteract this problem, we decided to replace the hydroxyl with a methoxy group [16]. This simple modification significantly increases the photostability of the probe and, at the same time, simplifies the photophysics by preventing the ESIPT reaction from occurring.

We also introduced the diethylamino group into the conjugation, strategically positioned to act as a donor for these push–pull probes. This incorporation is known to shift the absorption and emission to longer wavelengths compared to the parent chromophore, making it more attractive for bioimaging applications. In our group, diethylaminohydroxyflavone has already been studied; it offers attractive photophysical properties such as large Stokes shifts, red-shifted emission wavelengths, and high quantum yields in non-aqueous media. On this basis, the methoxy version was therefore a logical next step in our research project on probe engineering for application in DNA sensing. Two molecules were developed with an alkyne (**AlMF**) and azide (**AzMF**) functional group, making them easy to conjugate with the target of interest through a Cu(I)-mediated click chemistry approach. It is noteworthy that these anchor points were located at position 6 (Scheme 1), as modifying this position of the benzo ring should not generate changes in photophysics. Indeed, this position is not mesomerically coupled to the acceptor carbonyl group and the inductive effects are negligible at four bonds away. Thus, this is an appropriate location to bind fluorescence reporters to DNA.

**Scheme 2:**
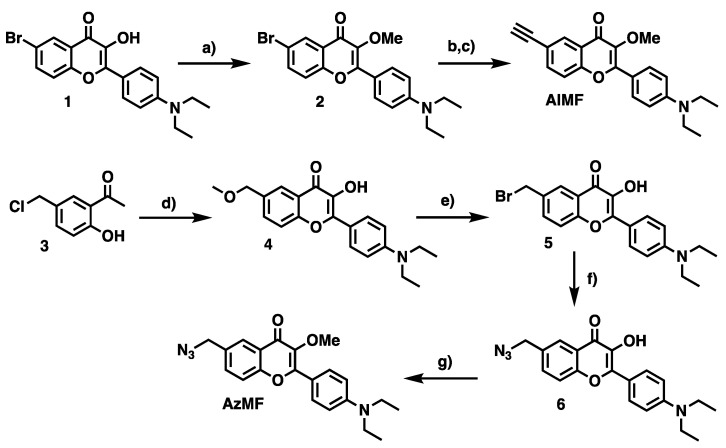
Synthetic routes to **AlMF** and **AzMF**: (a) Me_2_SO_4_ (4 eq.), 18-crown-6 (7 mol%), KOH, H_2_O/DCM, rt, 88%. (b) TMS-acetylene (3 eq.), TEA (10 eq.), CuI (20 mol%), PdCl_2_(PPh_3_)_3_ (20 mol%), DMF, 70 °C. (c) K_2_CO_3_, MeOH, rt, 64% over 2 steps. (d) *p*-Diethylaminobenzaldehyde (1.05 eq.), NaOH (1.05 eq.), H_2_O_2_, MeOH, rt, 97%. (e) HBr (47% in H_2_O), reflux, quant. (f) NaN_3_ (1.5 eq.), Acetone/DMF. (g) Me_2_SO_4_ (4 eq.), 18-crown-6 (7 mol%), KOH, H_2_O/DCM, rt, 70% over 2 steps.

The chromone core for **AlMF** was synthesized according to our previously reported strategy [21]. As for the methylation of the 3-OH group, it was performed following an in-house protocol based on phase-transfer conditions (PTC) to afford the corresponding methyl ether **2** (Scheme 2) [22]. The ethynyl moiety was finally introduced using a standard Sonogashira coupling with TMS-acetylene. Subsequent cleavage of the silyl group provided **AlMF**, the first targeted fluorescent label.

To avoid coupling the azide directly to the aromatic ring [23], an alternative synthetic route was considered for **AzMF.** The first step consists in the construction of the 3HC scaffold via an Algar-Flynn-Oyamada reaction [24] between *p*-diethylaminobenzaldehyde and **3** bearing a benzylic chloride, which was concomitantly displaced by a methoxide anion to yield **4**. Then, using harsh acidic conditions with conc. aqueous HBr solution, a bromide was incorporated in place of the methoxy group. A nucleophilic substitution on this benzyl position introduced the azide functionality required for post-synthetic labeling. Finally, to obtain **AzMF**, the 3-hydroxyl group was methylated using the same PTC procedure as **AlMF** (*vide supra*).

**Scheme 3:**
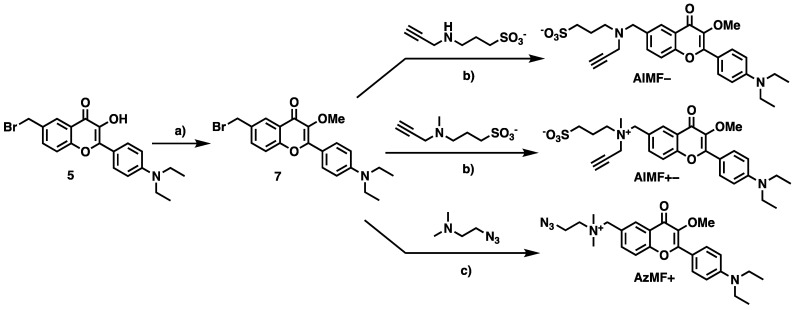
Synthetic routes to **AlMF−**, **AlMF+−**, and **AzMF+**: (a) Me_2_SO_4_ (4 eq.), 18-crown-6 (7 mol%), KOH, H_2_O/DCM, 80%. (b) K_2_CO_3_ (2.5 eq.), H_2_O/DMF, [AlMF−, 95%], [AlMF+−, 36%]. (c) THF, 63%.

It is worth mentioning that the conversion of the 3-OH into the 3-OMe group induces a drop in the hydrophilicity of the chromophore. To counterbalance this effect, three linkers were designed to significantly improve the hydrophilic character of **AlMF** and **AzMF**. By introducing this type of tethers, we also wanted to study the influence of the charge on the photophysical and DNA interaction properties of the dye. The first one carries a negative charge (**AlMF−**), while the second a positive one (**AzMF+**) and the last is a zwitterionic label (**AlMF+−**) (Scheme 3). The synthetic procedure of these three charged fluorophores is very similar to the **AlMF** and **AzMF** models.

**Scheme 4:**
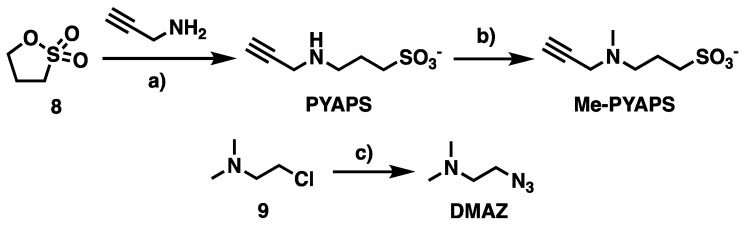
Synthetic pathways for the linkers **PYAPS**, **Me-PYAPS**, and **DMAZ**: (a) CH_3_CN, 72%. (b) Formol, formic acid, quant. (c) NaN_3_ (3 eq.), H_2_O, quant.

The first step, conversely to **AzMF,** is the establishment of the methoxy group leading to **7**. Since the introduction of a charge will complicate the purification process, it is wise to perform it in the last stage. The final step is the substitution of the benzyl bromide of **7** by three small amino molecules, two of them are described here for the first time (**PYAPS**, **Me-PYAPS**, Scheme 4). Note that owing to these clickable connectors, it is now possible to convert any fluorophore bearing a functional group which is prone to substitution on its main architecture, into its ionic conjugate with significantly increases water solubility. In this way, the possibility of modifying lipophilic probes for use in a polar solvent is opened.

### 2.2. Photophysics

**Scheme 5:**
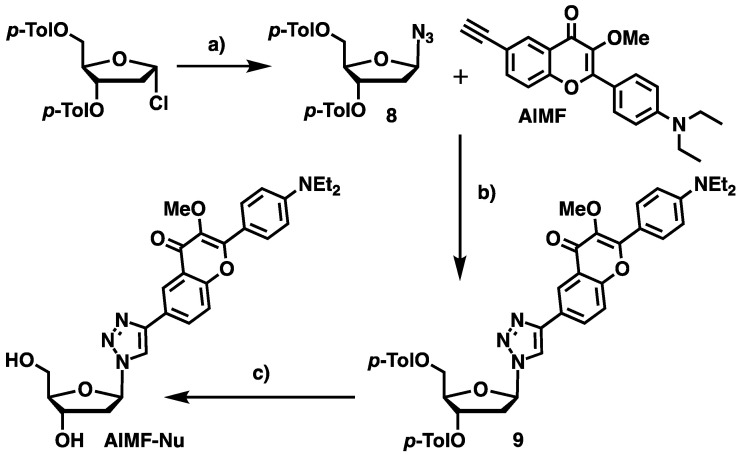
Synthetic access to the **AIMF**-derived model nucleoside **AlMF-Nu**: (a) NaN_3_ (5 eq.), Acetone, 0 °C, 80%. (b) DIPEA (12 eq.), AcOH (6 eq.), CuI (2.8 eq.), DCE, 40 °C, 98%. (c) K_2_CO_3_, MeOH/DCM, 35 °C, 56%.

To obtain an idea about the photophysical behavior of these fluorescent dyes in a DNA environment, a simple clicked model compound was designed, with the hope that this modified nucleoside could simulate the same photophysics. Thus, **AlMF-Nu** was synthesized using a standard CuAAC approach where **AlMF** was efficiently clicked with the 3′,5′-*bis*-toluoyl azido sugar **8**, followed by cleavage of the esters of **9** by methanolysis (Scheme 5).

**Figure 2:**
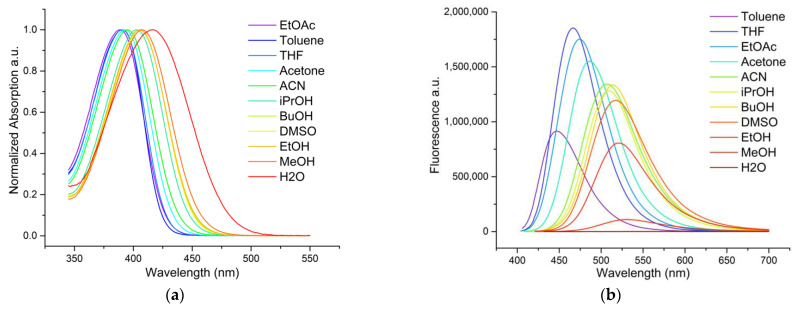
Absorption (**a**) and fluorescence (**b**) spectra of **AlMF-Nu**.

Our photophysical characterization began by investigating the spectroscopic features of these three **dEAF**-based fluorophores in methoxy series (**AlMF**, **AzMF,** and **AlMF-Nu**) in several solvent environments with varying polarity and H-bonding ability. All of these three dyes follow the same trend in their absorption and emission properties, including quantum yields, in accordance with our expectations (Table S1, Supplementary Materials). Those values are comparable to those of the reference methoxychromone (**dEAMF)** [18], without significant differences. For example, in MeOH, the maxima of the absorption and emission spectra oscillate between 405–410 nm and 529–533 nm (*Φ* = 4.1–5%), respectively for **AlMF**, **AzMF,** and **AlMF-Nu**, significantly close to the **dEAMF** photophysical features (*λ*_Abs_ = 405 nm, *λ*_Em_ = 501 nm, *Φ* = 4.7%). These results confirmed that modifications introduced at position 6 have little influence on the photophysics compared to the parent chromone. Since **AlMF**, **AzMF,** and **AlMF-Nu** belong to a class of push–pull dyes, they show a progressive redshift in absorption and emission maxima when moving from a lower to a higher polarity solvent, as expected (positive solvatochromism, Figure 2 and Table S1, Supplementary Materials). 

However, these bathochromic shifts are more pronounced in emission than in absorption (e.g., for **AlMF-Nu**, compare the wavelength range between toluene and MeOH in absorption 391–407 nm and emission 448–529 nm, ∆*λ* = 16 vs. 81 nm). In line with **dEAMF** and other similar chromones conjugating strong electron-donating groups, this observation indicates that even though the dipole moments have parallel directions, the one in the excited state is larger than that in the ground state [25]. This means that the stabilization of the excited state by the solvent is therefore more important, and consequently the excited state has a greater sensitivity to polarity. As for the reference 3-methoxychromone **dEAMF**, low quantum yields were noticed in polar protic solvents, while high efficiencies were observed in apolar media. Such behavior is typical of push–pull dyes.

**Figure 3:**
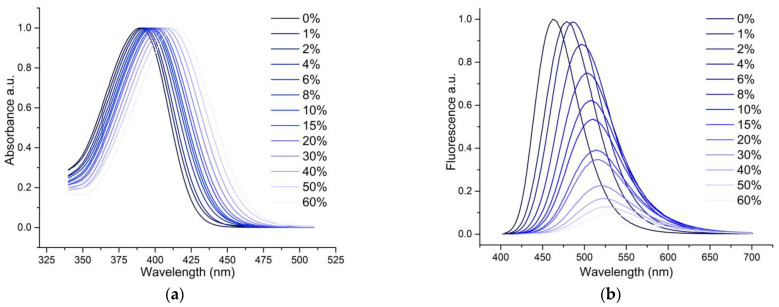
Absorption (**a**) and fluorescence (**b**) responses of **AlMF** to hydration. Data were recorded in gradual mixtures of water in THF.

Interestingly, **AlMF** exhibits an exquisite sensitivity to the local hydration rate via three channels of information: a bathochromic *λ*-shift in absorption and emission as well as fluorescence intensity. Indeed, hydration studies revealed that increasing the water content to 10% results in a radical drop in quantum yield of ca. 50% (Figure 3 and Table S2, Supplementary Materials). This represents the most desirable feature for our developed dyes, as it opens the door to turn-on hybridization applications. Indeed, accommodation or base stacking of the reporter upon annealing should shield it from a water exposure and lead to a fluorescence signal enhancement.

**Figure 4:**
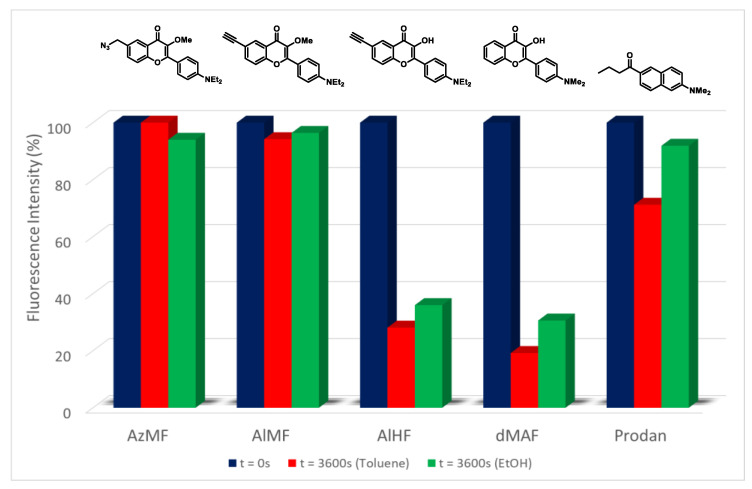
Photobleaching evolution of **AzMF**, **AlMF**, **AlHF**, and push–pull references **dMAF** and **Prodan** over the course of a one-hour experiment in toluene and EtOH.

To discover whether the advanced photophysics of the labels are compatible with a physiological environment, a pH titration was performed and provided a p*K*_A_ value of approx. 3.7 (Figures S1 and S2, Supplementary Materials). This result guarantees a stable fluorescence intensity over a comfortable working pH range of 5 to strongly basic media without risking a possible protonation of the tertiary aniline, the concomitant loss of the push–pull relationship and its associated photophysical properties. The photostability of the fluorescent markers was also checked to ensure that they are suitable for laser excitations, typical of bioimaging detection. To do so, the considered fluorophores were irradiated for 1 h with high-intensity xenon light at their absorption maximum, while monitoring their fluorescence intensity in protic and non-protic media. The decay in the number of photons collected by the detector means that a part of the dye population is photobleached. As a result, it is clear that methoxy derivatives (**AzMF** and **AlMF**) are considerably more photostable than their hydroxy counterparts (**AlHF** and the standard D-*π*-A 3HC **dEAF**), even more than the reference push–pull fluorophore, Prodan, which is an asset to be emphasized (Figure 4 and Figure S3, Supplementary Materials).

**Figure 5:**
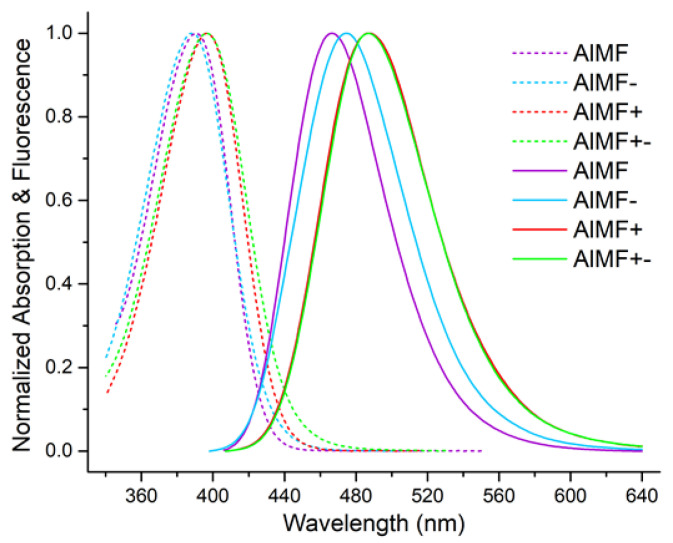
Photophysical comparison of neutral and charged dyes in THF. Dashed and solid lines refer to absorbance and fluorescence observables, respectively.

As for the charged conjugates, the p*K*_A_ value and photostability were preserved since no modifications of the aniline moiety and the conjugation were made. In comparison with the reference compound **AlMF**, the main difference lies in the solvatochromic character. The positively charged and zwitterionic derivatives, respectively **AlMF+** and **AlMF+−**, displayed many differences from the neutral form (Table S3 and Figures S4–S9, Supplementary Materials), such as solvatochromism in absorption and emission; the solvatofluorochromism being more exacerbated. These effects are less pronounced for the negatively charged **AlMF−**. One of the possible explanations is based on the distance and position of the charge with respect to the push–pull core (Scheme 3). The electric field produced by the proximal positive charge and the electron-rich carbonyl oxygen leads to an increase in the push–pull relationship between the donor and the acceptor. This results in a red-shifted absorption and emission of fluorophores [26], which is the consequence of what is known as the Stark effect, already reported for **dEAMF** (Figure 5 and Table S3, Supplementary Materials) [25].

### 2.3. Spectroscopic Studies of Labeled ODNs

In this study, a post-synthetic CuAAC strategy was adopted with our synthesized push–pull dyes to tag single-stranded (ss) ODNs bearing varied azide and alkyne modifications. In order to optimize the fluorescence turn-on upon hybridization, sequences of diverse composition (AT- and GC-rich, polyA) with various linkers differing in size, flexibility, position onto the nucleotide, and location on the strand were selected (Table 1). Indeed, with a more or less short tether, three of them consist in a C5-modification of a central T base, and two others are positioned at the 5′-end. Finally, the last connector is ultra-short since it is directly attached to the anomeric position of the ribose unit, and thus allows the substitution of a nucleobase. By combining all these transformations, 10 sequences could be labeled and screened for their fluorogenic behavior upon hybridization (Table 1 and Table S4, Supplementary Materials).

A classical Cu(I)-mediated click protocol was employed to fluorescently label the listed ODNs. Once clicked onto the ODNs, since the charged conjugates demonstrated very similar photophysics to **AlMF** and **AzMF** (Tables S1–S3, Supplementary Materials) as well as to keep the work compact and concise, only **AlMF** and **AzMF** were described in the hybridization studies. As noticed in former works, post-synthetic click reaction is very sensitive to the type of fluorophores used. In fact, the main issue in ODN labeling is related to the solubility of the dye. Another important point is the concentration of each reagent; a slight modification can drastically affect the yields. The ligand employed proves to be the cornerstone for obtaining outstanding yields. Starting the screening with the first-generation copper ligand (**TBTA**), labeling yields limited to 20% were observed; this is probably due to its poor solubility in water. Next, the water-soluble **THPTA** ligand was tested, and a clear improvement in performance was noticed, as the yield was more than doubled (reaching about 50%). Finally, using one of the latest generation ligands, **BTTES**, excellent yields were achieved (~90%) [27]. All labeled ODNs were analyzed and purified by RP-HPLC (Figures S10 and S11, Supplementary Materials). Their integrity and mass were confirmed by spectrophotometry and HRMS (Tables S5 and S6, Supplementary Materials). Indeed, labeled ODNs bear a typical signature in UV–Vis spectroscopy, corresponding to the absorption ratio between 260 nm (nucleobases) and 420–450 nm (clicked fluorophore), which should match with the ratio of their extinction coefficient (for dyes: ε_max_ ≃ 41,000 M^−1^·cm^−1^).

First, the room temperature stability of the duplexes—formed from ss-ODNs modified with our dyes—was controlled by determining their melting temperature (Tables S7 and S8 and Figures S12–S14, Supplementary Materials). Then, the photophysical properties of the labeled ss-ODNs and their ds-constructs—obtained after hybridization of the complementary strand—were investigated (Table 2, charged probes in Table S9 and Figures S15–S17, Supplementary Materials). Interestingly, when the dyes were bound at the 5′-end, no increase in fluorescence intensity was noted and this observation was true for all sequence types with their respective linkers (Figures S18 and S19, Supplementary Materials). It is worth mentioning that the maximum fluorescent enhancement was obtained for the AT-rich context with the **X**-linker, while the most red-shifted absorption was remarked for the **W**-linker, i.e., the ethynyl-ribose located in the middle of the sequence.

Note that those functionalized with the **Z**- and **Xs**-linkers showed only a small to almost no increment. These results clearly suggest that the chain length of the tether is crucial to obtain a turn-on emission upon hybridization. This would explain why **Z**- and **Xs**-linkers with medium-sized chain lengths do not allow a proper folding of the tag and thus, displayed almost no change in their fluorescence emission. Conversely, when the linker is long enough such as **X**, it provides sufficient flexibility for the fluorescent marker to interact with the grooves or force the intercalation during annealing. Thus, shielding its exposure to water allows the dye to recover its fluorescence potential and causes, in addition, redshifts in absorption by accommodation or stacking with adjacent nucleobases. Similarly, when the linker is short such as **W** and directly connected to the deoxyribose moiety, the clicked fluorophore is forced to intercalate inside the duplex with the neighboring base pairs, leading to the same photophysical conclusion as for **X**-tether (*Φ*⬈, red-shifted *λ*_Abs_).

In all the cases, the duplex formation was confirmed by an almost 2-fold increase in absorption at 260 nm. For the GC-rich sequence, almost no notable change in fluorescence intensity upon hybridization was detected. This can be explained by the reducing propensity of the guanine base, which is known to quench most of the fluorophores by photoinduced electron transfer (PET) process [1,29]. Alternatively, GC-rich duplexes being more stable are perhaps less prone to fluorophore intercalation.

It is worth noting that in all labeled sequences studied, absorption maxima of single- and double-stranded probes are identical to those of the hydrophilic charged dyes in bulk water (***λ*_Abs_** ≈ 430 nm, Table 2, Tables S2 and S3, Supplementary Materials). This is therefore evidence of a highly hydrated environment around the fluorescent reporter, with the exception of the **TXT** vs. **TXT·***AAA* and to a lesser extent **AXA** vs. **AXA·***TAT* combinations for which a significant bathochromic shift is observed upon hybridization (10–20 nm). This result confirmed the ability of the aromatic fluorophore **AlMF** to stack with the neighboring base pairs [30].

**Figure 6:**
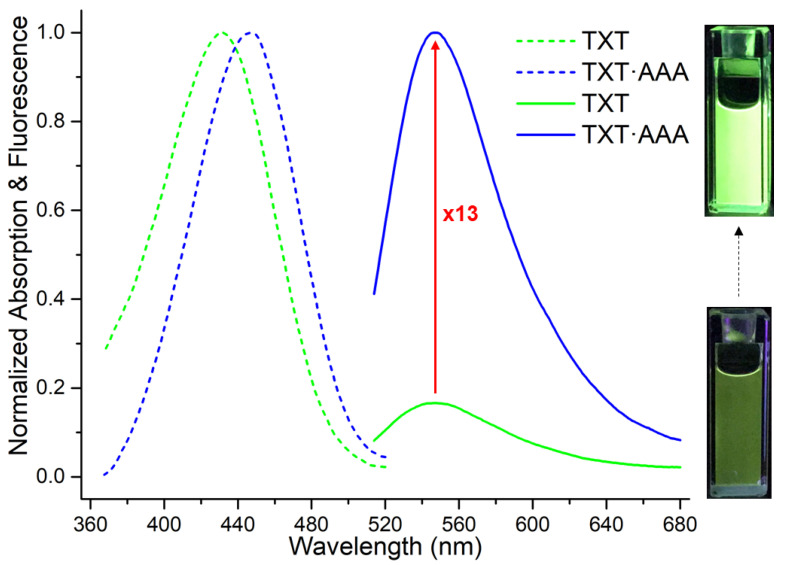
Absorbance (dashed) and fluorescence (solid) observables of **TXT** and **TXT•***AAA* (488-nm excitation).

To determine accordingly the highest fluorescence amplification from ss- to ds-ODNs labeled with **AlMF**, given that a 20-nm redshift occurred between the two contexts, the entire absorption range was examined to define the most appropriate excitation wavelength (**TXT** vs. **TXT·***AAA*, Table 3 and Figure 6 and Figure S20, Supplementary Materials). It was clear that excitation in the red edge of the ss absorption band should trigger a lower ss emission and thus, a higher contrast. Nevertheless, exciting too far from the absorption maximum should affect the brightness considerably. A good balance was found at the argon laser excitation (490-nm region), where a remarkable 13-fold fluorogenic turn-on was noticed, as attested by the bright green–yellow color of the cuvette inserts. With respect to the **AXA** sequence, almost a manifold fluorescence increase was recorded upon hybridization (**AXA** vs. **AXA·***TAT*, Table S10 and Figure S21, Supplementary Materials).

## 3. Materials and Methods

### 3.1. General Procedures

All reactions involving air- and water-sensitive conditions were performed in oven-dried glassware under argon by using Schlenk techniques employing a dual vacuum/argon manifold system and dry solvents. The synthetic intermediates were initially co-evaporated twice with toluene and dried in vacuo before use. All chemical reagents were purchased from commercial sources (Sigma-Aldrich (Saint-Louis, MO, USA), Acros (Geel, Belgium), Alfa Aesar (Heysham, Lancashire, UK)) and were used as supplied. Anhydrous solvents were obtained according to standard procedures [31]. The reactions were monitored simultaneously by liquid chromatography-mass spectrometry (LC-MS) and thin-layer chromatography (TLC, silica gel 60 F254 plates). Compounds were visualized on TLC plates by both UV radiation (254 and 365 nm) and spraying with a staining agent (phosphomolybdic acid, KMnO_4_ or ninhydrin) followed by subsequent warming with a heat gun. Column chromatography was performed with flash silica gel (40−63 µm) with the indicated solvent system using gradients of increasing polarity in most cases [32]. All NMR spectra (^1^H, ^13^C, and 2D) were recorded on 200 or 400 MHz Bruker advance spectrometers (Bruker, Billerica, MA, USA). The ^1^H-NMR (200 and 400 MHz) and ^13^C{^1^H}-NMR (50 and 101 MHz, recorded with complete proton decoupling) spectra were obtained with samples dissolved in CDCl_3_, CD_2_Cl_2_, CD_3_OD or DMSO-d_6_ with the residual solvent signals used as internal references: 7.26 ppm for C*H*Cl_3_, 5.32 ppm for CD*H*Cl_2_, 3.31 ppm for CD_2_*H*OD, 2.50 ppm for (CD_3_)(CD_2_*H*)S(O) regarding ^1^H-NMR experiments, and 77.2 ppm for CDCl_3_, 53.8 ppm for CD_2_Cl_2_, 49.0 ppm for CD_3_OD, 39.4 ppm for (CD_3_)_2_S(O) concerning ^13^C-NMR experiments [33]. Chemical shifts (δ) are given in ppm to the nearest 0.01 (^1^H) or 0.1 ppm (^13^C). The coupling constants (*J*) are given in hertz (Hz). The signals are reported as follows (s = singlet, d = doublet, t = triplet, m = multiplet, br = broad). Assignments of ^1^H and ^13^C-NMR signals were achieved with the help of D/H exchange, COSY, HMQC, HSQC, NOESY and HMBC experiments (Figures S22–S38). LC-MS spectra were recorded using an ion trap Esquire 3000 Plus mass spectrometer (Bruker, Billerica, MA, USA) equipped with an electrospray ionization (ESI) source in both positive and negative modes. High-resolution mass spectrometry (HRMS) was conducted with a hybrid ion trap-Orbitrap mass spectrometer (Thermo Fisher Scientific, Bremen, Germany)—combining quadrupole precursor selection with high-resolution and accurate-mass Orbitrap detection—using ESI techniques. Systematic flavone and nucleoside nomenclatures are used below for the spectral assignment of each synthesized derivative (Schemes S1–S4, Supplementary Materials). All solvents for absorption and fluorescence experiments were of spectroscopic grade. Absorbance spectra were recorded on a Cary 100 Bio UV-Vis spectrophotometer (Varian/Agilent, Palo Alto, CA, USA) using Suprasil^®^ quartz 500-µL cuvettes (Haraeus, Hanau, Germany) with 1-cm path length. Stock solutions of the fluorescent nucleoside or ODNs were prepared using THF or Milli-Q^®^ water (Merck Millipore, Burlington, MA, USA). The nucleoside sample used for spectroscopic measurements contained ≈0.2% (*v*/*v*) of the stock solution solvent. Fluorescence measurements were conducted on a FluoroMax 4.0 spectrofluorometer (Jobin Yvon, Horiba, Kyoto, Japan) with a thermostatically controlled cell compartment at 20 ± 0.5 °C with slits open to 2 nm and were corrected for Raman scattering, lamp fluctuations and instrumental wavelength-dependent bias. Emission spectra were performed with an absorbance of about 0.05. The excitation wavelength corresponds to the absorption maximum of the considered sample, except when specified. Quantum yields were corrected according to the variation of the refractive index of the different solvents. They were determined by using *p*-DiMethylAminoFlavone (DMAF) in EtOH (*λ*_Ex_ = 404 nm, *Φ* = 27%) as a reference [28], with ±10% mean standard deviation. Fluorescent nucleoside **AlMF-Nu** was analyzed in duplicate at 10 and 2 µM, respectively for UV–Vis and fluorescence measurements. Labeled ODNs were analyzed in duplicate at 2 µM in phosphate-buffered saline pH 7.4 (50 mM sodium phosphate, 150 mM NaCl). In order to ensure reproducibility of hybridization and therefore of measurements, the double-stranded samples were first denatured and then cooled to room temperature.

### 3.2. Synthetic Procedures

*6-Bromo-2-(4-diethylamino)phenyl-3-hydroxy-4H-chromen-4-one* (**1**): 1-(5-Bromo-2-hydroxyphenyl)ethanone (1.7 g, 7.75 mmol, 1 eq.) and 4-(diethylamino)benzaldehyde (1.56 g, 8.52 mmol, 1.1 eq.) were solubilized in 1,2-dichloroethane (10 mL) and morpholine (10 mL). The reaction solution was irradiated by microwave (25 min, 100 °C, 200 W). The volatiles were removed in vacuo to give a reddish brown solid, which was solubilized in ethanol (15 mL). To the stirred mixture cooled in an ice bath, were added sequentially an aq. 5 M NaOH solution (20 mL, 13 eq.) and H_2_O_2_ (30% *w*/*w* in H_2_O, 7.65 mL, 10 eq.). After stirring overnight, the mixture was neutralized by an aq. 1 N HCl solution, and the resulting precipitate was filtered and washed with water and cyclohexane sequentially. Compound **1** was obtained as an orange solid. (2.24 g, 77%). R_f_ = 0.38 (Toluene/Acetone 4:1) or 0.58 (DCM/MeOH, 99.5:0.5). ^1^H-NMR (CDCl_3_, 200 MHz): *δ* 1.15 (t, ^3^*J* = 7.0 Hz, 6H, NCH_2_-C*H_3_*), 3.41 (q, ^3^*J* = 7.0 Hz, 4H, NC*H_2_*-CH_3_), 6.67 (d, ^3^*J* = 9.0 Hz, 2H, H*_meta_*), 7.35 (d, ^3^*J* = 8.8 Hz, 1H, H8), 7.62 (dd, ^3^*J* = 8.8 Hz, ^4^*J* = 2.4 Hz, 1H, H7), 8.05 (d, ^3^*J* = 9.0 Hz, 2H, H*_ortho_*), 8.26 (d, ^4^*J* = 2.4 Hz, 1H, H5), 9.37 (s, 1H, OH). ^13^C-NMR (CDCl_3_, 50 MHz): *δ* 12.7 (N-CH_2_-*C*H_3_), 44.5 (N-*C*H_2_-CH_3_), 110.9 (C*_m_*), 116.7 (C*_p_*), 119.8 (C*_i_*), 122.3 (C8), 127.6 (C6), 129.5 (C7), 135.5 (C*_o_*), 136.9 (C5), 147.3 (C10), 149.1 (C2), 153.7 (C9), 171.0 (C4). HRMS (ESI^+^): *m*/*z* calcd for C_19_H_18_NO_3_BrH^+^: 388.0543, 390.0522 [M + H]^+^; found: 388.0550, 390.0529 [M + H]^+^.

*6-Bromo-2-(4-diethylamino)phenyl-3-methoxy-4H-chromen-4-one* (**2**): To a stirred suspension of **1** (110 mg, 0.33 mmol, 1 eq.) in DCM (0.25 M, 6 mL), were sequentially added 18-crown-6 (25 mg, 7 mol%), an aq. KOH solution (25% *w*/*w*, 0.9 mL) and dimethyl sulfate (139 µL, 4 eq.). The reaction mixture was stirred 30 min at rt. After addition of H_2_O (8 mL), the organic layer was extracted with DCM (3×), dried over MgSO_4_, filtered and the volatiles were removed in vacuo. The residue was purified by flash chromatography on silica gel eluted with DCM/DCM–1% MeOH mixture (90:10 → 10:90, *v*/*v*) to provide the desired compound **2** as a yellow powder (101 mg, 0.4 mmol, 88%). R_f_ = 0.29 (DCM/MeOH 99.5:0.5). ^1^H-NMR (CDCl_3_, 400 MHz): *δ* 1.16 (t, ^3^*J* = 7.1 Hz, 6H, NCH_2_-C*H_3_*), 3.38 (d, ^3^*J* = 7.1 Hz, 4H, NC*H_2_*-CH_3_), 3.81 (s, 3H, OCH_3_), 6.67 (d, ^3^*J* = 9.3 Hz, 2H, H*_meta_*), 7.32 (d, ^3^*J* = 8.9 Hz, 1H, H8), 7.62 (dd, ^3^*J* = 8.9 Hz, ^4^*J =* 2.4 Hz, 1H, H7), 8.00 (d, ^3^*J* = 9.3 Hz, 2H, H*_ortho_*), 8.30 (d, ^4^*J* = 2.4 Hz, 1H, H5). ^13^C-NMR (CDCl_3_, 101 MHz): *δ* 12.6 (NCH_2_-*C*H_3_), 44.5 (N*C*H_2_-CH_3_), 56.7 (OCH_3_), 110.8 (C*_meta_*), 116.4 (C6), 117.6 (C*_i_*), 119.6 (C8), 127.7 (C10), 128.2 (C5), 130.3 (C*_ortho_*), 135.7 (C7), 140.0 (C3), 149.6 (C*_para_*), 153.8 (C2), 157.0 (C9), 173.2 (C4). HRMS (ESI^+^): *m*/*z* calcd for C_20_H_20_BrNO_3_H^+^: 402.0699, 404.0679 [M + H]^+^; found: 402.0698, 404.0679 [M + H]^+^.

*2-(4-(Diethylamino)phenyl)-3-methoxy-6-((trimethylsilyl)ethynyl)-4H-chromen-4-one* (**AlMF-TMS**): To a stirred solution of **2** (50 mg, 0.12 mmol) in dry DMF (3 mL) under argon, were sequentially added TMS-acetylene (53 µL, 0.37 mmol, 3 eq.), triethylamine (175 µL, 1.24 mmol, 10 eq.) and a mixture of CuI (5 mg, 20 mol%)/PdCl_2_(PPh_3_)_2_ (18 mg, 20 mol%). The reaction mixture was warmed to 70 °C overnight. The volatiles were removed in vacuo and the residue was directly engaged in the next step.

*2-(4-(Diethylamino)phenyl)-6-ethynyl-3-methoxy-4H-chromen-4-one* (**AlMF**): To a 10-mL vial containing **AlMF-TMS** (20 mg, 45 µmol, 1 eq.), was added a minimum of MeOH (2 mL) to solubilize the compound. K_2_CO_3_ (63 mg, 0.45 mmol, 10 eq.) was then poured, and the resulting solution was stirred overnight at room temperature. The reaction mixture was quenched by acetic acid and reduced in vacuo to give the crude product. The residue was purified by preparative TLC (SiO_2_) eluted with DCM/MeOH (99:1, *v*/*v*) to provide the desired product **AlMF** as a yellow powder (10 mg, 30 µmol, 64% over 2 steps). R_f_ = 0.24 (DCM/MeOH 99.5:0.5). ^1^H-NMR (CDCl_3_, 400 MHz): *δ* 1.16 (t, ^3^*J* = 7.2 Hz, 6H, NCH_2_-C*H_3_*), 3.04 (s, 1H, HC≡C), 3.38 (d, ^3^*J* = 7.2 Hz, 4H, NC*H_2_*-CH_3_), 3.82 (s, 3H, OCH_3_), 6.67 (d, ^3^*J* = 9.2 Hz, 2H, H*_meta_*), 7.38 (d, ^3^*J* = 8.7 Hz, 1H, H8), 7.62 (dd, ^3^*J* = 8.7, ^4^*J* = 2.0 Hz, 1H, H7), 8.00 (d, ^3^*J* = 9.2 Hz, 2H, H*_ortho_*), 8.31 (d, ^4^*J* = 2.0 Hz, 1H, H5). ^13^C-NMR (CDCl_3_, 101 MHz): *δ* 12.6 (NCH_2_-*C*H_3_), 44.5 (N*C*H_2_-CH_3_), 59.7 (OCH_3_), 77.8 (H*C*≡C), 82.3 (HC≡*C*), 110.8 (C*_meta_*), 116.5 (C6), 118.0 (C8), 118.4 (C*_i_*), 124.2 (C10), 129.9 (C5), 130.2 (C*_ortho_*), 136.0 (C7), 140.0 (C3), 149.5 (C*_para_*), 154.7 (C2), 156.8 (C9), 173.7 (C4). HRMS (ESI^+^): *m/z* calcd for C_22_H_21_NO_3_H^+^: 348.1594 [M + H]^+^; found: 348.1600.

*1-(5-(Chloromethyl)-2-hydroxyphenyl)ethan-1-one* (**3**): Adapted from the protocol described in [34]. To a solution of paraformaldehyde (2.4 g, 79.3 mmol, 1.08 eq.) in conc. HCl solution (45 mL), was added 2-hydroxyacetophenone (8.85 mL, 73.4 mmol, 1 eq.). Next, the reaction mixture was stirred at 35 °C for 5 h. A yellow precipitate was formed which was then filtered and washed with water (3×). The resulting solid was solubilized in DCM, dried over MgSO_4_, filtered and the volatiles were removed in vacuo to obtain the product as a yellow powder (11.8 g, 63.9 mmol, 87%). R_f_ = 0.52 (Cyclohexane/EtOAc 4:1). ^1^H-NMR (DMSO-d_6_, 400 MHz): *δ* 2.63 (s, 3H, H9), 4.76 (s, 2H, H8), 6.98 (d, ^3^*J* = 8.5 Hz, 1H, H4), 7.59 (dd, ^3^*J* = 8.5 Hz, ^4^*J* = 2.2 Hz, 1H, H5), 7.95 (d, ^4^*J* = 2.2 Hz, 1H, H7). ^13^C-NMR (DMSO-d_6_, 101 MHz): *δ 28*.0 (C9), 45.9 (C8), 118.2 (C4), 120.5 (C2), 128.5 (C6), 131.8 (C7), 136.9 (C5), 160.6 (C3), 203.8 (C1).

*2-(4-(Diethylamino)phenyl)-3-hydroxy-6-methoxymethyl-4H-chromen-4-one* (**4**): To a stirred solution of **3** (2.5 g, 13.5 mmol, 1 eq.) in methanol (45 mL, 0.3 M) was added NaOH (1.66 g, 14.2 mmol, 1.05 eq.) and 4-(diethylamino)benzaldehyde (2.55 g, 14.2 mmol, 1.05 eq.). The reaction mixture was refluxed for 8 h. After cooling to 4 °C, H_2_O_2_ (30% *w*/*w* in H_2_O, 5.5 mL) was added dropwise. Then, cold water (200 mL) was poured, and the reaction mixture was acidified with an aq. 2 N HCl solution to pH 6.5. MeOH was evaporated under reduced pressure. DCM (200 mL) was added, and the organic layer was extracted (3×). The combined organic phases were reduced in vacuo to give the crude compound **4** as a brown oil, which was pure enough to be taken to the next step without further purification (4.66 g, 13.2 mmol, 97%). R_f_ = 0.44 (Cyclohexane/EtOAc 4:1). ^1^H-NMR (DMSO-d_6_, 400 MHz): *δ* 1.16 (t, ^3^*J* = 7.0 Hz, 6H, NCH_2_-C*H_3_*), 3.33 (s, 3H, OCH_3_), 3.42 (q, ^3^*J* = 7.0 Hz, 4H, NC*H_2_*-CH_3_), 4.54 (s, 2H, OC*H_2_*Ph), 6.80 (d, ^3^*J* = 9.2 Hz, 2H, H*_meta_*), 7.68 (m, 2H, H7 and H8), 8.01 (d, ^4^*J* = 2.0 Hz, 1H, H5), 8.09 (d, ^3^*J* = 9.2 Hz, 2H, H*_ortho_*). ^13^C-NMR (CDCl_3_, 101 MHz): *δ* 12.0 (NCH_2_-*C*H_3_), 43.3 (N*C*H_2_-CH_3_), 57.2 (OCH_3_), 72.3 (OCH_2_), 110.3 (C*_meta_*), 116.5 (C6), 117.7 (C8), 118.1 (C*_i_*), 122.5 (C5), 124.2 (C10), 128.8 (C*_ortho_*), 131.9 (C7), 142.6 (C3), 146.6 (C*_para_*), 148.0 (C2), 153.1 (C9), 171.3 (C4).

*6-(Bromomethyl)-2-(4-(diethylamino)phenyl)-3-hydroxy-4H-chromen-4-one* (**5**): A mixture of **4** (610 mg, 1.7 mmol, 1 eq.) and 47% hydrobromic acid in H_2_O (5 mL) was refluxed for 3 h. After cooling to room temperature, a saturated aq. solution of sodium carbonate was added slowly to neutralize the reaction until pH 7 was reached. Then, the solid was filtered to directly obtain the desired crude solid **5** as a khaki solid (960 mg, 2.4 mmol, quant.), which was directly committed to the next step without further purification. R_f_ = 0.24 (Cyclohexane/EtOAc 4:1). ^1^H-NMR (CDCl_3_, 400 MHz): *δ* 1.23 (t, ^3^*J* = 7.1 Hz, 6H, NCH_2_-C*H_3_*), 3.45 (q, ^3^*J* = 7.1 Hz, 4H, NC*H_2_*-CH_3_), 4.60 (s, 2H, OC*H_2_*Ph), 6.77 (d, ^3^*J* = 9.1 Hz, 2H, H*_meta_*), 7.54 (d, ^3^*J* = 8.7 Hz, 1H, H8), 7.69 (dd, ^3^*J* = 8.7 Hz, ^4^*J* = 2.1 Hz, 1H, H7), 8.16 (d, ^3^*J* = 9.1 Hz, 2H, H*_ortho_*), 8.22 (d, ^4^*J* = 2.1 Hz, 1H, H5).

*6-(Azidomethyl)-2-(4-(diethylamino)phenyl)-3-hydroxy-4H-chromen-4-one* (**6**): **5** (242 mg, 0.60 mmol, 1 eq.) was solubilized in a mixture of acetone (6 mL) and DMF (1.5 mL) to which was added NaN_3_ (60 mg, 0.90 mmol, 1.5 eq.). The solution was stirred overnight at room temperature. Then, EtOAc (20 mL) was introduced, and acetone was evaporated under reduced pressure. A saturated aq. solution of NH_4_Cl was added (10 mL) to quench the reaction. The organic layer was extracted with EtOAc (×2), dried over MgSO_4_, filtered, and concentrated in vacuo. The crude product was purified by flash chromatography on silica gel eluted with cyclohexane/ethyl acetate (9:1 → 7:3, *v*/*v*) to give **6** as an orange powder, which was directly engaged in the next step.

*6-Azidomethyl-2-(4-(diethylamino)phenyl)-3-methoxy-4H-chromen-4-one* (**AzMF**): To a stirred solution of **6** (144 mg, 0.40 mmol, 1 eq.) in CH_2_Cl_2_ (2.6 mL), were sequentially added 18-crown-6 (30 mg, 7 mol%), an aq. KOH solution (25% *w*/*w*, 0.4 mL) and dimethyl sulfate (166 µL, 1.58 mmol, 4 eq.). The resulting mixture was stirred overnight at room temperature. After quenching the reaction by addition of H_2_O (5 mL), the organic layer was extracted with CH_2_Cl_2_ (3×). The combined organic phases were dried over MgSO_4_, filtered and the volatiles were removed in vacuo. The residue was purified by flash chromatography on silica gel eluted with cyclohexane/ethyl acetate (9:1 → 3:1, *v*/*v*) to give the desired compound as a yellow solid (146 mg, 0.38 mmol, 70% over 2 steps). ^1^H-NMR (CDCl_3_, 400 MHz): *δ* 1.17 (t, ^3^*J* = 7.1 Hz, 6H, NCH_2_-C*H_3_*), 3.39 (q, ^3^*J* = 7.1 Hz, 4H, NC*H_2_*-CH_3_), 3.83 (s, 3H, OCH_3_), 4.39 (s, 2H, N_3_C*H_2_*Ph), 6.68 (d, ^3^*J* = 9.3 Hz, 2H, H*_meta_*), 7.46 (d, ^3^*J* = 8.6 Hz, 1H, H8), 7.54 (dd, ^3^*J* = 8.6 Hz, ^4^*J* = 2.2 Hz, 1H, H7), 8.02 (d, ^3^*J* = 9.3 Hz, 2H, H*_ortho_*), 8.22 (d, ^4^*J* = 2.2 Hz, 1H, H5). ^13^C-NMR (CDCl_3_, 101 MHz): *δ* 12.6 (NCH_2_-*C*H_3_), 44.5 (N*C*H_2_-CH_3_), 54.2 (N_3_*C*H_2_Ph), 59.7 (OCH_3_), 110.8 (C*_meta_*), 116.6 (C6), 118.6 (C8), 124.3 (C*_i_*), 125.3 (C5), 130.2 (C*_ortho_*), 131.7 (C10), 132.6 (C7), 140.1 (C3), 149.5 (C*_para_*), 154.8 (C2), 156.8 (C9), 174.2 (C4). HRMS (ESI^+^): *m*/*z* calcd for C_21_H_22_N_4_O_3_H^+^: 379.1765; [M + H]^+^; found: 379.1777.

*6-(Bromomethyl)-2-(4-(diethylamino)phenyl)-3-methoxy-4H-chromen-4-one* (**7**): To a stirred solution of **5** (240 mg, 0.60 mmol, 1 eq.) in DCM (12 mL), were sequentially added 18-crown-6 (79 mg, 0.30 mmol, 0.1 eq.), an aq. KOH solution (25% *w*/*w*, 1.7 mL, DCM/KOH 7:1) and dimethyl sulfate (282 µL, 2.98 mmol, 5 eq.). The reaction mixture was stirred for 30 min at room temperature. The organic layer was extracted with DCM (3×), dried over MgSO_4_, filtered, and concentrated in vacuo. The residue was then purified by flash chromatography on silica gel eluted with cyclohexane/ethyl acetate (9:1 → 7:3, *v*/*v*) to give the desired compound **7** as a red oil (200 mg, 0.48 mmol, 80%). R_f_ = 0.58 (Cyclohexane/EtOAc 3:1). ^1^H-NMR (CDCl_3_, 400 MHz): *δ* 1.23 (t, ^3^*J* = 7.1 Hz, 6H, NCH_2_-C*H_3_*), 3.45 (q, ^3^*J* = 7.1 Hz, 4H, NC*H_2_*-CH_3_), 3.88 (s, 3H, OCH_3_), 4.59 (s, 2H, BrC*H_2_*Ph), 6.74 (d, ^3^*J* = 9.2 Hz, 2H, H*_meta_*), 7.49 (d, ^3^*J* = 8.7 Hz, 1H, H8), 7.67 (dd, ^3^*J* = 8.7 Hz, ^4^*J* = 2.2 Hz, 1H, H7), 8.08 (d, ^3^*J* = 9.2 Hz, 2H, H*_ortho_*), 8.24 (d, ^4^*J* = 2.2 Hz, 1H, H5). ^13^C-NMR (CDCl_3_, 101 MHz): *δ* 12.6 (NCH_2_-*C*H_3_), 32.4 (Br*C*H_2_Ph), 44.5 (N*C*H_2_-CH_3_), 59.7 (OCH_3_), 110.8 (C*_meta_*), 116.6 (C6), 118.6 (C8), 124.2 (C*_i_*), 125.8 (C5), 130.2 (C*_ortho_*), 133.7 (C7), 134.1 (C10), 140.1 (C3), 149.5 (C*_para_*), 154.7 (C2), 156.8 (C9), 174.1 (C4). HRMS (ESI^+^): *m*/*z* calcd for C_21_H_22_BrNO_3_H^+^: 416.0856, 418.0835 [M + H]^+^; found: 416.0870, 418.0848.

*Acid 3-(prop-2-yn-1-ylamino)propane-1-sulfonic* (**PYAPS**): To a solution of propargylamine (116 µL, 1.8 mmol, 1 eq.) in CH_3_CN (5 mL) was added dropwise 1,3-propane sultone **8** (169 µL, 1.9 mmol, 1.05 eq.). The reaction mixture was stirred at room temperature for 2 d. The resulting precipitate was filtered and washed with CH_3_CN to afford the desired compound **PYAPS** as a pinkish solid (230.5 mg, 1.31 mmol, 72%). R_f_ = 0.25 (DCM/MeOH 4:1). Green staining by ninhydrin. ^1^H-NMR (DMSO-d_6_, 400 MHz): *δ* 1.93 (p, ^3^*J* = 6.8 Hz, 2H, H5), 2.60 (t, ^3^*J* = 6.8 Hz, 2H, H4), 3.09 (t, ^3^*J* = 6.8 Hz, 2H, H6), 3.68 (t, ^4^*J* = 2.5 Hz, 1H, H1), 3.90 (d, ^4^*J* = 2.5 Hz, 2H, H3). ^13^C-NMR (DMSO-d_6_, 101 MHz): *δ* 21.7 (C5), 35.6 (C3), 46.0 (C4), 48.8 (C6), 75.1 (C2), 79.4 (C1). HRMS (ESI^+^): *m*/*z* calcd for C_6_H_11_NO_3_SH^+^: 178.0532; [M + H]^+^; found: 178.0534.

*Sodium 3-(methyl(prop-2-yn-1-yl)amino)propane-1-sulfonate* (**Me-PYAPS**): **PYAPS** (150 mg, 0.85 mmol, 1 eq.), formol (37% formaldehyde in H_2_O) (900 µL) and formic acid (903 µL) were heated overnight at 70 °C. Then, the solution was concentrated in vacuo until a paste was obtained. NaHCO_3_ (1 g) was added to the solid until bubbling ceased to provide the sodium sulfonate salt **Me-PYAPS** as a white solid (375 mg, quant.). Bicarbonate salts contaminate the product due to the excess used. ^1^H-NMR (DMSO-d_6_, 400 MHz): *δ* 1.60–1.73 (m, 2H, H5), 2.15 (s, 3H, H7), 2.32–2.42 (m, 4H, H4 and H6), 3.07 (t, ^4^*J* = 2.4 Hz, 1H, H1), 3.25 (d, ^4^*J* = 2.4 Hz, 2H, H3). ^13^C-NMR (DMSO-d_6_, 101 MHz): *δ* 22.7 (C5), 40.7 (C3), 44.3 (C6), 48.9 (C7), 53.7 (C4), 75.1 (C2), 81.5 (C1). HRMS (ESI^+^): *m*/*z* calcd for C_7_H_12_NO_3_SH^+−^: 192.0689; [M + H]^+^; found: 192.0690.

*2-Azido-N,N-dimethylethanamine* (**DMAZ**): 3-Chloro-1,1-dimethylpropylamine **9** (800 mg, 5.6 mmol, 1 eq.) and NaN_3_ (1.1 g, 16.7 mmol, 3 eq.) were sequentially dissolved in H_2_O (18.5 mL, 0.3 M). The solution was stirred at 80 °C for 24 h. After cooling to room temperature, the reaction mixture was adjusted to pH 10 by addition of an aq. 0.5 M NaOH solution. The organic layer was then extracted with diethyl ether (3×). The combined extracts were dried over MgSO_4_, filtered, and concentrated under vacuum to yield the product **DMAZ** as a yellowish liquid (970 mg, 9.03 mmol, quant.). ^1^H-NMR (CDCl_3_, 400 MHz): *δ* 2.24 (s, 6H, H3 and H3′), 2.47 (t, ^3^*J* = 6.2 Hz, 2H, H2), 3.31 (t, ^3^*J* = 6.2 Hz, 2H, H1). ^13^C-NMR (CDCl_3_, 101 MHz): *δ* 45.5 (C3 and C3′), 49.1 (C1), 58.0 (C2). HRMS (ESI^+^): *m*/*z* calcd for C_4_H_10_N_4_H^+−^: 115.0978; [M − H]^+^; found: 115.0982.

*Potassium N(3-(((2-(4-(diethylamino)phenyl)-3-methoxy-4H-chromen-6-yl)methyl), N(prop-2-yn-1-yl)amino)propane-1-sulfonate* (**AlMF−**): To a solution of **7** (22 mg, 50 µmol, 1 eq.) in DMF (0.5 mL), were added **PYAPS** (19 mg, 0.11 mmol, 2 eq.), H_2_O (0.4 mL), and K_2_CO_3_ (19 mg, 0.13 mmol, 2.5 eq.). The solution was stirred at room temperature for 24 h. Then, DMF and water were evaporated in vacuo and the crude (solubilized with DCM/MeOH) was purified by flash chromatography on silica gel eluted with *i*-PrOH/NH_3_/H_2_O (12:2:1, *v*/*v*) to afford the desired product **AlMF−** as an orange solid (26 mg, 50 µmol, 95%). ^1^H-NMR (MeOD, 400 MHz): *δ* 1.22 (t, *^3^J* = 7.0 Hz, 6H, NCH_2_-C*H_3_*), 1.97–2.08 (m, 2H, H6′), 2.73 (t, *^3^J* = 6.9 Hz, 2H, H5′), 2.90 (m, 2H, H7′), 3.49 (q, *^3^J* = 7.0 Hz, 4H, NC*H_2_*-CH_3_), 3.78 (m, 2H, H2′), 3.79 (s, 3H, OCH_3_), 3.94 (d, *^3^J* = 2.6 Hz, 1H, H4′), 4.84 (s, 2H, H1′), 6.80 (d, *^3^J* = 7.0 Hz, 2H, H*_meta_*), 7.60 (d, *^3^J* = 8.6 Hz, 1H, H8), 7.77 (dd, *^3^J* = 8.6 Hz, *^2^J* = 2.1 Hz 1H, H7), 8.07 (s, 1H, H5), 8.08 (m, *^3^J* = 7.0 Hz, 2H, H*_ortho_*). HRMS (ESI^+^): *m*/*z* calcd for C_27_H_32_N_2_O_6_SH^+^: 513.2054; [M − H]^+^; found: 513.2064.

*3-(((2-(4-(diethylamino)phenyl)-3-methoxy-4-oxo-4H-chromen-6-yl)methyl)(methyl)(prop-2-yn-1-yl)ammonio)propane-1-sulfonate* (**AlMF+−**): To a solution of **7** (80 mg, 0.19 mmol, 1 eq.) in DMF (4.5 mL), were added **Me-PYAPS** and water (2 mL). The reaction mixture was stirred at room temperature for 24 h before the volatiles were removed under vacuum. The residue (diluted with DCM/MeOH) was purified by flash chromatography on silica gel eluted with *i*-PrOH/NH_3_/H_2_O (12:2:1, *v*/*v*) to give the desired product **AlMF+−** as a yellow oil (36 mg, 68 µmol, 36%). ^1^H-NMR (MeOD, 400 MHz): *δ* 1.23 (t, *^3^J* = 7.1 Hz, 6H, NCH_2_-C*H_3_*), 2.07–2.17 (m, 2H, H6′), 2.86 (s, 3H, H8′), 2.88 (m, 2H, H5′), 3.07–3.14 (m, 2H, H7′), 3.50 (q, *^3^J* = 7.1 Hz, 4H, NC*H_2_*-CH_3_), 3.68–3.69 (m, 5H, H2′ and OCH_3_), 4.30 (m, 1H, H4′), 4.79 (s, 2H, H1′), 6.81 (d, *^3^J* = 9.0 Hz, 2H, H*_meta_*), 7.72 (d, *^3^J* = 8.6 Hz, 1H, H8), 7.96 (dd, *^3^J* = 8.6 Hz, *^2^J* = 2.0 Hz 1H, H7), 8.07 (d, *^3^J* = 9.0 Hz, 2H, H*_ortho_*), 8.35 (s, 1H, H5). ^13^C-NMR (MeOD, 101 MHz): *δ* 11.5, 21.0, 44.0, 44.7, 53.8, 58.7, 60.9, 64.4, 71.1, 77.6, 82.6, 110.7, 115.4, 119.2, 123.5, 123.9, 130.0, 130.2, 137.1, 139.5, 150.1, 156.0, 158.0, 173.9. HRMS (ESI^+^): *m*/*z* calcd for C_28_H_34_N_2_O_6_SH^+^: 527.2210; [M + H]^+^; found: 527.2215.

*2-Azidoethyl-N-((2-(4-(diethylamino)phenyl)-3-methoxy-4-oxo-4H-chromen-6-yl)methyl)-N,N-dimethylethanammonium bromide* (**AzMF+**): To a solution of **7** (12 mg, 29 µmol, 1 eq.) in anhydrous THF (0.4 mL, 80 mM), was added **DMAZ** (16 mg, 0.144 mmol, 5 eq.). The reaction mixture was stirred at room temperature for 6 h. The formed precipitate was centrifuged, and the upper layer was discarded. The compound was washed twice more after suspension in dry THF, centrifugation and removal of the supernatant. The recovered solid was dried under reduced pressure to provide **AzMF+** as a yellow powder (9.6 mg, 18 µmol, 63%). ^1^H-NMR (MeOD, 400 MHz): *δ* 1.24 (t, *^3^J* = 7.1 Hz, 6H, NCH_2_-C*H_3_*), 3.16 (s, 6H, H2′and H3′), 3.52 (q, *^3^J* = 7.1 Hz, 4H, NC*H_2_*-CH_3_), 3.62 (t, *^3^J* = 5.4 Hz, 2H, H5′), 3.83 (s, 3H, OCH_3_), 4.08 (t, *^3^J* = 5.4 Hz, 2H, H4′), 4.76 (s, 2H, H1′), 6.84 (d, *^3^J* = 9.3 Hz, 2H, H*_meta_*), 7.82 (d, *^3^J* = 8.6 Hz, 1H, H8), 7.93 (d, *^3^J* = 8.6 Hz, 1H, H7), 8.13 (d, *^3^J* = 9.3 Hz, 2H, H*_ortho_*), 8.35 (s, 1H, H5). ^13^C-NMR (MeOD, 101 MHz): *δ* 11.5 (NCH_2_-*C*H_3_), 44.0 (N*C*H_2_-CH_3_), 44.6 (C2′, C3′), 49.5 (N_3_CH_2_), 54.2 (N^+^*C*H_2_Ph), 58.7 (OCH_3_), 62.6 (CH_2_N^+^), 67.6 (N^+^*C*H_2_Ph), 110.8 (C*_meta_*), 115.4 (C6), 119.1 (C8), 123.8 (C_i_), 123.9 (C10), 130.2 (C5), 130.2 (C*_ortho_*), 137.2 (C7), 137.2 (C3), 139.6 (C*_para_*), 150.2 (C2), 156.1 (C9), 174.1 (C4). HRMS (ESI^+^): *m*/*z* calcd for C_25_H_32_N_5_O_3_^+^: 450.2500 [M − H]^+^; found: 450.2500.

*1-Azido-1,2-dideoxy-3,5-di-O-p-toluoyl-β-d-ribofuranose* (**8**): Adapted from the procedure described in [35,36]. To a stirred solution of 1α-chloro-3,5-di-toluoyl-2-deoxy-l-ribose (1.4 g, 3.64 mmol, 1 eq.) in acetone (20 mL) at 0 °C, was slowly added NaN_3_ (1.22 g, 18.2 mmol, 5.0 eq.). The reaction mixture was kept under vigorous stirring for 5 h. After completion, as evidenced by TLC monitoring, the reaction mixture was quenched with a saturated aq. NaHCO_3_ solution (15 mL), and the organic layer was extracted with CH_2_Cl_2_ (3×). The combined extracts were dried over MgSO_4_, filtered, and evaporated under reduced pressure. The crude oil obtained is a mixture of both anomers (*β*/*α* 9:1), which was subjected to flash chromatography on silica gel eluted with PE/Et_2_O (19:1 → 4:1, *v*/*v*) to furnish the pure *β* anomer of **8** as a white crystalline compound (1.0 g, 80%). C_21_H_23_O_5_N_3_ (384.13). R_f_ *=* 0.67 (Toluene/Acetone 9:1). ^1^H-NMR (CD_2_Cl_2_, 400 MHz): *δ* 2.43 (s, 3H, C*H_3_*-Tol), 2.44 (s, 3H, C*H_3_*-Tol), 2.46–2.49 (m, 2H, H2′), 4.55–4.67 (m, 3H, H4′, H5′), 5.64 (ddd, ^3^*J* = 7.9 Hz, ^3^*J* = 4.4Hz, ^4^*J* = 2.2 Hz, 1H, H3′), 5.77 (t, ^3^*J* = 5.1 Hz, 1H, H1′), 7.28–7.31 (m, 4H, H*_m_*_-Tol_), 7.98 (d, ^3^*J* = 8.2 Hz, 2H, H*_o_*_-Tol_), 8.05 (d, ^3^*J* = 8.2 Hz, 2H, H*_o_*_-Tol_). ^13^C-NMR (CDCl_3_, 50 MHz): *δ* 22.1 (CH_3_), 22.1 (CH_3_), 39.3 (C2′), 65.0 (C5′), 75.7 (C4′), 83.5 (C3′), 93.0 (C1′), 127.5 (C*_i_*), 127.9 (C*_i_*), 129.9 (C*_m_*), 129.9 (C*_m_*), 130.4 (C*_o_*), 130.4 (C*_o_*), 144.7 (C*_p_*), 145.1 (C*_p_*), 166.5 (CO), 166.8 (CO).

*1,2-Dideoxy-1β-(4-(2-(4-(diethylamino)phenyl)-3-methoxy-4H-chromen-6-yl)-1H-1,2,3-triazol-1-yl)-3,5-di-O-p-toluoyl-β-d-ribofuranose* (**9**): To a stirred solution of **AlMF** (10 mg, 30 µmol, 1 eq.) in DCE (2.5 mL) inside a 4-mL vial, were sequentially added **8** (18 mg, 50 µmol, 1.8 eq.), DIPEA (61 µL, 0.35 mmol, 12 eq.), acetic acid (10 µL, 0.17 mmol, 6 eq.) and CuI (16 mg, 80 µmol, 2.8 eq.). The reaction was heated at 40 °C for 1 h under an argon atmosphere to give a homogeneous blue solution. The mixture was concentrated under reduced pressure and the residue was purified by preparative TLC (SiO_2_) eluted with DCM/MeOH (99:1, *v*/*v*) to give the desired product **9** as a yellow powder (21 mg, 30 µmol, 98%). ^1^H-NMR (CDCl_3_, 400 MHz): *δ* 1.14 (t, *^3^J* = 7.0 Hz, 6H, NCH_2_-C*H_3_*), 2.28 and 2.37 (2s, 6H, H12 and H14), 2.84 (ddd, *^2^J* = 14.2 Hz, *^3^J* = 6.1 Hz, *^3^J* = 3.3 Hz, 1H, H2′), 3.16–3.25 (m, 1H, H2′), 3.3–3.44 (m, 4H, NC*H_2_*-CH_3_), 3.84 (s, 3H, OCH_3_), 4.49–4.54 (m, 1H, H5′), 4.58–4.66 (m, 2H, H5′, H4′), 5.71–5.77 (m, 1H, H3′), 6.47 (t, *^3^J* = 6.3 Hz, 1H, H1′), 6.69 (d, *^3^J* = 9.2 Hz, 2H, H*_m_*), 7.12 (d, *^3^J* = 8.0 Hz, 2H, H*_m_*_′_/H*_m_*_′′_), 7.21 (d, *^3^J* = 8.2 Hz, 2H, H*_m_*_′_/H*_m_*_′′_), 7.49 (d, *^3^J* = 8.7 Hz, 1H, H8), 7.80 (d, *^3^J* = 8.0 Hz, 2H, H*_o_*_′_/H*_o_*_′′_), 7.90 (d, *^3^J* = 8.2 Hz, 2H, H*_o_*_′_/H*_o_*_′′_), 8.04 (m, 3H, Hα, H*_o_*), 8.15 (dd, *^3^J* = 8.7 Hz, *^2^J* = 2.2 Hz, 1H, H7), 8.36 (d, *^2^J* = 2.2 Hz, 1H, H5). ^13^C-NMR (CDCl_3_, 101 MHz): *δ* 12.6 (NCH_2_-*C*H_3_), 15.3 (C12/C14), 21.7 (C12/C14), 38.2 (C2′), 44.5 (N*C*H_2_-CH_3_), 59.7 (OCH_3_), 63.8 (C5′), 74.8 (C3′), 83.8 (C4′), 89.0 (C1′), 110.9 (C*_m_*), 116.7 (C_i_), 118.4 (C8), 118.9 (C*α*), 122.3 (C5), 124.4 (C6), 126.4 (C_i′_/C_i′′_), 126.6 (C_i′_/C_i′′_), 126.8 (C10), 129.3 (C*_m_*_′_/C*_m_*_′′_), 129.7 (C*_o_*_′_/C*_o_*_′′_), 129.8 (C*_o_*_′_/C*_o_*_′′_), 130.2 (C*_o_*), 130.5 (C7), 140.3 (C3), 144.1 (C*_p_*_′_/C*_p_*_′′_), 144.5 (C*_p_*_′_/C*_p_*_′′_), 147.0 (C*β*), 149.5 (C*_p_*), 154.8 (C9), 156.7 (C2), 165.9 (C11/C13), 166.2 (C11/C13), 172.7 (C4).

*1,2-Dideoxy-1-(4-(2-(4-(diethylamino)phenyl)-3-methoxy-4-oxo-4H-chromen-6-yl)-1H-1,2,3-triazol-1-yl)-β-d-ribofuranose* (**AlMF-Nu**): In a 4-mL vial, **9** (21 mg, 30 µmol, 1 eq.) was solubilized in MeOH (0.6 mL) and few drops of DCM. Then, K_2_CO_3_ (20 mg, 0.14 mmol, 5 eq.) was added and the reaction mixture was stirred overnight at 35 °C. The mixture was then quenched by a minimum of acetic acid and the solvents were removed in vacuo. The crude was purified by preparative RP-TLC (C18) eluted with H_2_O/acetonitrile (50:50, *v*/*v*) to give the desired product **AlMF-Nu** as a yellow solid (8 mg, 20 µmol, 56%). R_f_ = 0.11 (DCM/MeOH, 99.8:0.2). ^1^H-NMR (CDCl_3_, 400 MHz): *δ* 1.13 (t, *^3^J* = 7.0 Hz, 6H, NCH_2_-C*H_3_*), 2.47 (ddd, *^3^J* = 13.6 Hz, *^3^J* = 6.0 Hz, *^3^J* = 4.7 Hz, 1H, H2′), 2.70–2.79 (m, 1H, H2′), 3.35–3.44 (m, 4H, NC*H_2_*-CH_3_), 3.58 (dd, *^3^J* = 12.0 Hz, *^3^J* = 5.0 Hz, 1H, H5′), 3.68 (dd, *^3^J* = 12.0 Hz, *^3^J* = 3.9 Hz, 1H, H5′), 3.72 (s, 3H, OCH_3_), 3.97 (m, 1H, H4′), 4.50 (m, 1H, H3′), 6.38 (t, *^3^J* = 6.0 Hz, 1H, H1′), 6.71 (d, *^3^J* = 9.3 Hz, 2H, H*_meta_*), 7.62 (d, *^3^J* = 8.8 Hz, 1H, H8), 8.00 (d, *^3^J* = 9.2 Hz, 2H, H*_ortho_*), 8.12 (dd, *^3^J* = 8.8 Hz, *^3^J* = 2.1 Hz, 1H, H7), 8.44 (d, *^3^J* = 2.1 Hz, 1H, H5), 8.56 (s, 1H, Hα). ^13^C-NMR (CDCl_3_, 101 MHz): *δ* 11.5 (NCH_2_-*C*H_3_), 40.4 (C2′), 44.0 (N*C*H_2_-CH_3_), 58.7 (OCH_3_), 61.9 (C5′), 70.8 (C3′), 88.4 (C4′), 89.0 (C1′), 110,7 (C*_meta_*), 115.8 (C*_i_*), 118.5 (C8), 119.9 (C*α*), 121.3 (C5), 123.9 (C10), 127.3 (C6), 130.1 (C*_ortho_*), 130.6 (C7), 139.5 (C3), 146.2 (C*β*), 150.0 (C*_para_*), 154.8 (C9), 158.0 (C2), 174.6 (C4). HRMS (ESI^+^): *m*/*z* calcd for C_27_H_30_N_4_O_6_H^+^: 507.2238 [M + H]^+^; found: 507.2240.

## 4. Conclusions

In summary, novel fluorogenic derivatives of **dEAF** were synthesized with different kinds of linkers (from neutral to ionic) bearing appropriate functional groups, allowing the development of a toolbox of dyes, easy to conjugate with biomolecules. Compared to the parent chromone, these original fluorophores retained all photophysical properties intact, while improving solubility and photostability in aqueous media. They were then used to post-synthetically label ODNs of different compositions via Cu(I)-mediated click chemistry. The resulting ODN probes showed bright turn-on emission after annealing with the complementary strand. Position 6 of the chromone scaffold was chosen as the site of chemical modification because at this location, the impact on the photophysics of the dye is minimal. The methoxy group was preferred over the hydroxyl group to drastically enhance the photostability of the 3-OH chromone. A variety of tethers in terms of length and functionality were engineered and connected to different sites on the ODN (5′-end, internal C5-dT base, anomeric position). Conjugation of the dye at the 5′-terminus of the single strand generally demonstrated no change in its fluorescence emission upon hybridization due to its constant exposure to bulk water. However, when the dye was placed in the middle of the ODN, the quantum yield increased 13-fold compared to the single-stranded context. More importantly, this result—obtained after selective excitation at 488 nm (matching with the argon laser commonly used on all fluorescence microscopes)—still revealed a prospective Stokes shift of 56 nm. In this regard, our fluorogenic **AlMF** conjugate compares favorably with the TO/JO FIT-probes, which were synthesized to generate a FRET pair and a larger Stokes shift for easier and more sensitive detection [37]. We believe that these dyes will prove very helpful for diagnostic and bioimaging purposes by targeting specific nucleic acid sequences.

## Figures and Tables

**Table 1:** Azide- and alkyne-functionalized ODNs used for post-synthetic fluorescent labeling with engineered dyes.

Sequence	Functionalization (Y, X, Xs, V, Z and W) *
5′-**Y****CAG** TCG CTC GCT GAC-3′	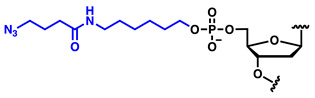
5′-**Y****GCA** AAA TTT AAA ACG-3′	
5′-CAG TCG **C**X**C** GCT GAC-3′	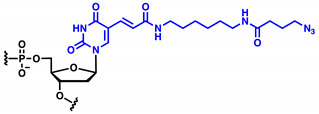
5′-GCA AAA **TXT** AAA ACG-3′	
5′-GCA AAA AAA **AXA** AAA AAA ACG-3′	
5′-GCA AAA **TXsT** AAA ACG-3′	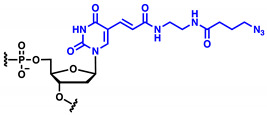
5′-**VGCA** AAA TTT AAA ACG-3′	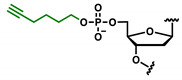
5′-GCA AAA **TZT** AAA ACG-3′	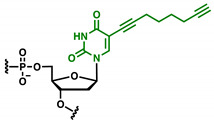
5′-GCA AAA **TWT** AAA ACG-3′	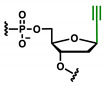
5′-GCA AAA AAA **AWA** AAA AAA ACG-3′	

* Linkers ending in an azido group or a terminal alkyne are depicted in indigo or green, respectively.

**Table 2:** Photophysical features of the considered ODNs labeled with **AlMF** and **AzMF**.

Sequences	*λ*_Abs_ (nm) ^a^		*λ*_Em_ (nm) ^b^		*Φ* (%) ^c^	
	AlMF	AzMF	AlMF	AzMF	AlMF	AzMF
**Y** **CAG**	426	—	542	—	17	—
**Y** **CAG·** *GTC*	427	—	543	—	7	—
**Y** **GCA**	431	—	542	—	12	—
**Y** **GCA·** *CGT*	429	—	544	—	5	—
**C** **X** **C**	424	—	542	—	21	—
**C** **X** **C·** *GAG*	427	—	542	—	15	—
**T** **X** **T**	432	—	542	—	16	—
**T** **X** **T·** *AAA*	449	—	546	—	41	—
**A** **XA**	432	—	550	—	11	—
**A** **XA·** *TAT*	441	—	547	—	34	—
**T** **XsT**	433	—	539	—	24	—
**T** **XsT·** *AAA*	430	—	542	—	22	—
**VGCA**	—	427	—	545	—	12
**VGCA·** *CGT*	—	421	—	545	—	5
**T** **ZT**	—	430	—	545	—	16
**T** **ZT·** *AAA*	—	431	—	545	—	28
**T** **WT**	—	431	—	547	—	15
**T** **WT·** *AAA*	—	437	—	542	—	30
**A** **WA**	—	428	—	552	—	8
**A** **WA·** *TAT*	—	429	—	531	—	34

^a^ Position of the absorption band maximum; ^b^ Position of the emission band maximum; ^c^ Quantum yield determined using *p*-dimethylaminoflavone (dMAF) in EtOH (*λ*_Ex_ = 404 nm, *Φ* = 27%) as a standard [28]. Linkers ending in an azido group or a terminal alkyne are depicted in indigo or green, respectively.

**Table 3:** Spectroscopic properties of **TXT** labeled with **AlMF**.

*λ*_Ex_ (nm) ^a^	TXT	TXT·*AAA*	Absorptivity Ratio ^c^	Fluorescence Intensity Ratio ^d^
	Brightness (L·mol^−1^·cm^−1^) ^b^			
430	6560	15,265	0.78	1.8
450	5350	16,810	1.11	3.7
480	1476	7572	1.84	10.4
490	764	4301	1.98	13.1
500	381	2054	1.96	13.4
510	212	760	1.90	12.9

^a^ Screening of the excitation wavelength; ^b^ Brightness calculation: absorptivity at the excitation wavelength × Quantum Yield; ^c^ Absorbance of ds/Absorbance of ss; ^d^ Fluorescence intensity of ds/Fluorescence intensity of ss.

## Data Availability

Experimental data are available within this research article and in the related Supplementary Materials.

## Supplementary Materials

The following supporting information can be downloaded at: https://www.mdpi.com/article/10.3390/molecules27072267/s1. Schemes S1–S4: Overview of the synthesis schemes; Tables S1–S3 and Figures S1–S9: Photophysical characterization; Tables S4–S10 and Figures S10–S21: Spectroscopic studies of model odns; Figures S22–S38: NMR spectra. Reference [38] are cited in the supplementary materials.

## Sample Availability

Samples of the compounds are not available from the authors.

## References

[1] Michel B.Y., Dziuba D., Benhida R., Demchenko A.P., Burger A. (2020). Probing of Nucleic Acid Structures, Dynamics, and Interactions With Environment-Sensitive Fluorescent Labels. Front. Chem..

[2] Braselmann E., Rathbun C., Richards E.M., Palmer A.E. (2020). Illuminating RNA Biology: Tools for Imaging RNA in Live Mammalian Cells. Cell Chem. Biol..

[3] Tomoike F., Abe H. (2019). RNA imaging by chemical probes. Adv. Drug Deliv. Rev..

[4] Jullien L., Gautier A. (2015). Fluorogen-based reporters for fluorescence imaging: A review. Methods Appl. Fluoresc..

[5] Klymchenko A.S. (2017). Solvatochromic and Fluorogenic Dyes as Environment-Sensitive Probes: Design and Biological Applications. Acc. Chem. Res..

[6] Suseela Y.V., Narayanaswamy N., Pratihar S., Govindaraju T. (2018). Far-red fluorescent probes for canonical and non-canonical nucleic acid structures: Current progress and future implications. Chem. Soc. Rev..

[7] Lakowicz J.R. (2006). Principles of Fluorescence Spectroscopy.

[8] Wang Y.-N., Xu B., Qiu L.-H., Sun R., Xu Y.-J., Ge J.-F. (2021). Viscosity sensitive fluorescent dyes with excellent photostability based on hemicyanine dyes for targeting cell membrane. Sens. Actuators B.

[9] Cao J., Hu C., Liu F., Sun W., Fan J., Song F., Sun S., Peng X. (2013). Mechanism and Nature of the Different Viscosity Sensitivities of Hemicyanine Dyes with Various Heterocycles. ChemPhysChem.

[10] Köhler O., Jarikote D.V., Singh I., Parmar V.S., Weinhold E., Seitz O. (2005). Forced intercalation as a tool in gene diagnostics and in studying DNA–protein interactions. Pure Appl. Chem..

[11] Hövelmann F., Gaspar I., Chamiolo J., Kasper M., Steffen J., Ephrussi A., Seitz O. (2016). LNA-enhanced DNA FIT-probes for multicolour RNA imaging. Chem. Sci..

[12] Hövelmann F., Seitz O. (2016). DNA Stains as Surrogate Nucleobases in Fluorogenic Hybridization Probes. Acc. Chem. Res..

[13] Okamoto A. (2011). ECHO probes: A concept of fluorescence control for practical nucleic acid sensing. Chem. Soc. Rev..

[14] Chou P.-T., Martinez M.L., Clements J.H. (1993). The observation of solvent-dependent proton-transfer/charge-transfer lasers from 4’-diethylamino-3-hydroxyflavone. Chem. Phys. Lett..

[15] Klymchenko A.S., Ozturk T., Demchenko A.P. (2002). Synthesis of furanochromones: A new step in improvement of fluorescence properties. Tetrahedron Lett..

[16] Howard J.J., Lynch G.C., Pettitt B.M. (2011). Ion and solvent density distributions around canonical B-DNA from integral equations. J. Phys. Chem. B.

[17] Barthes N.P.F., Gavvala K., Dziuba D., Bonhomme D., Karpenko I.A., Dabert-Gay A.S., Debayle D., Demchenko A.P., Benhida R., Michel B.Y. (2016). Dual emissive analogue of deoxyuridine as a sensitive hydration-reporting probe for discriminating mismatched from matched DNA and DNA/DNA from DNA/RNA duplexes. J. Mater. Chem. C.

[18] Kucherak O.A., Richert L., Mély Y., Klymchenko A.S. (2012). Dipolar 3-methoxychromones as bright and highly solvatochromic fluorescent dyes. Phys. Chem. Chem. Phys..

[19] Fantoni N.Z., El-Sagheer A.H., Brown T. (2021). A Hitchhiker’s Guide to Click-Chemistry with Nucleic Acids. Chem. Rev..

[20] Amblard F., Cho J.H., Schinazi R.F. (2009). Cu(l)-catalyzed huisgen azide-alkyne 1,3-dipolar cycloaddition reaction in nucleoside, nucleotide, and oligonucleotide chemistry. Chem. Rev..

[21] Le H.-N., Zilio C., Barnoin G., Barthes N.P.F., Guigonis J.-M., Martinet N., Michel B.Y., Burger A. (2019). Rational design, synthesis, and photophysics of dual-emissive deoxyadenosine analogs. Dyes Pigments.

[22] Dziuba D., Benhida R., Burger A. (2011). A Mild and Efficient Protocol for the Protection of 3-Hydroxychromones Under Phase-Transfer Catalysis. Synthesis.

[23] Kolb H.C., Finn M.G., Sharpless K.B. (2001). Click Chemistry: Diverse Chemical Function from a Few Good Reactions. Angew. Chem. Int. Ed..

[24] Dean F.M., Podimuang V. (1965). 737. The course of the Algar–Flynn–Oyamada (A.F.O.) reaction. J. Chem. Soc..

[25] Nemkovich N.A., Baumann W., Pivovarenko V.G. (2002). Dipole moments of 4′-aminoflavonols determined using electro-optical absorption measurements of molecular Stark-effect spectroscopy. J. Photochem. Photobiol. A.

[26] Klymchenko A.S., Demchenko A.P. (2002). Electrochromic Modulation of Excited-State Intramolecular Proton Transfer: The New Principle in Design of Fluorescence Sensors. J. Am. Chem. Soc..

[27] Ivancová I., Leone D.-L., Hocek M. (2019). Reactive modifications of DNA nucleobases for labelling, bioconjugations, and cross-linking. Curr. Opin. Chem. Biol..

[28] Ormson S.M., Brown R.G., Vollmer F., Rettig W. (1994). Switching between charge-and proton-transfer emission in the excited state of a substituted 3-hydroxyflavone. J. Photochem. Photobiol. A.

[29] Dziuba D., Didier P., Ciaco S., Barth A., Seidel C.A.M., Mély Y. (2021). Fundamental photophysics of isomorphic and expanded fluorescent nucleoside analogues. Chem. Soc. Rev..

[30] Hainke S., Seitz O. (2009). Binaphthyl-DNA: Stacking and Fluorescence of a Nonplanar Aromatic Base Surrogate in DNA. Angew. Chem. Int. Ed..

[31] Armarego W.L.F., Chai C.L.L. (2017). Purification of Laboratory Chemicals.

[32] Still W.C., Kahn M., Mitra A. (1978). Rapid chromatographic technique for preparative separations with moderate resolution. J. Org. Chem..

[33] Fulmer G.R., Miller A.J.M., Sherden N.H., Gottlieb H.E., Nudelman A., Stoltz B.M., Bercaw J.E., Goldberg K.I. (2010). NMR Chemical Shifts of Trace Impurities: Common Laboratory Solvents, Organics, and Gases in Deuterated Solvents Relevant to the Organometallic Chemist. Organometallics.

[34] Wulff G., Akelah A. (1978). Enzyme-analogue built polymers, 6. Synthesis of 5-vinylsalicylaldehyde and a simplified synthesis of some divinyl derivatives. Die Makromol. Chem..

[35] Kolganova N.A., Florentiev V.L., Chudinov A.V., Zasedatelev A.S., Timofeev E.N. (2011). Simple and stereoselective preparation of an 4-(aminomethyl)-1,2,3-triazolyl nucleoside phosphoramidite. Chem. Biodivers..

[36] Štimac A., Kobe J. (2000). Stereoselective synthesis of 1,2-cis- and 2-deoxyglycofuranosyl azides from glycosyl halides. Carbohydr. Res..

[37] Hövelmann F., Gaspar I., Ephrussi A., Seitz O. (2013). Brightness Enhanced DNA FIT-Probes for Wash-Free RNA Imaging in Tissue. J. Am. Chem. Soc..

[38] Reichardt C. (1994). Solvatochromic dyes as solvent polarity indicators. Chem. Rev..

