# Supplementary material for: An Expeditious Approach towards the Synthesis and Application of Water-Soluble and Photostable Fluorogenic Chromones for DNA Detection
Source: Molecules. 2022 Mar 31;27(7):2267. doi: 10.3390/molecules27072267 (PMC9000371; doi:10.3390/molecules27072267)
Supplement: Supplementary file 1 [file molecules-27-02267-s001.zip › molecules-1642082-supplementary.pdf]

Supporting Information to:

An expeditious approach towards the synthesis and application of water-soluble and photostable fluorogenic chromones for DNA detection

Steve Vincent, Suman Mallick, Guillaume Barnoin, Hoang-Ngoan Le, Benoît Y. Michel* and Alain Burger*

Université Côte d'Azur (UCA), Institut de Chimie de Nice, UMR 7272, CNRS, Parc Valrose, 06108 Nice cedex 2 (France)

*Correspondence:

benoit.michel@univ-cotedazur.fr, *alain.burger@univ-cotedazur.fr*

Table of contents

EXPERIMENTAL SECTION **3**

1. OVERVIEW OF THE SYNTHESIS SCHEMES **3**
   1.1 Preparation of the 3-OMe chromone labels **3**
   1.2 Preparation of the ***AlMF****-based model nucleoside* **4**

2. PHOTOPHYSICAL CHARACTERIZATION **4**
   2.1 Absorptivity determination **4**
   2.2 Steady-state fluorescence measurements of neutral labels **4**
   2.3 pK_A study **5**
   2.4 Photobleaching studies **5**
   2.5 Hydration study **6**
   2.6 Steady-state fluorescence measurements of charged labels **7**
   2.7 Absorption & emission spectra of charged labels **8**

3. SPECTROSCOPIC STUDIES OF MODEL ODNs **9**
   3.1 ODN synthesis and purification **9**
   3.2 HRMS analysis of labeled ODNs **11**
   3.3 Temperature-induced denaturation studies **11**
   3.4 Steady-state fluorescence measurements **13**
   3.5 Absorbance & fluorescence spectra **14**

NMR SPECTRA **20**

Experimental Section

1. OVERVIEW OF THE SYNTHESIS SCHEMES

1.1 Preparation of the 3-OMe chromone labels

Scheme S1. Synthetic access to the alkyne derivative AlMF.

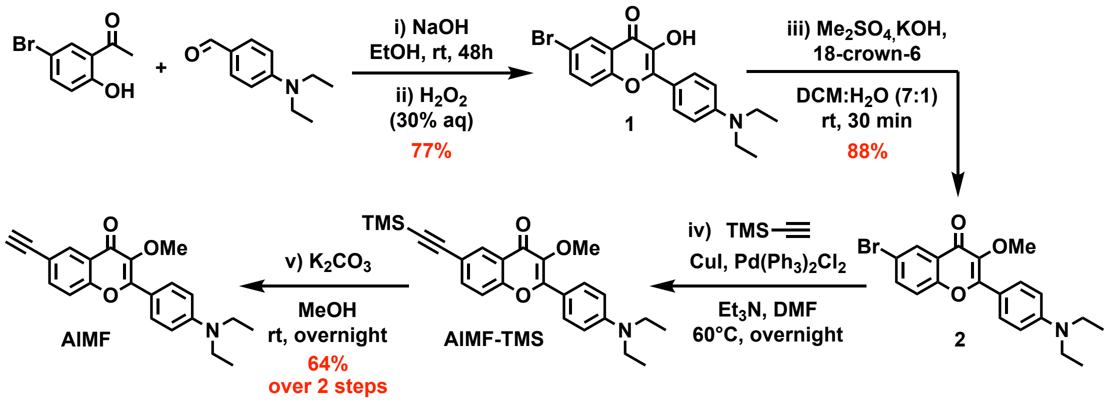

Scheme S2. Synthetic access to the azide derivative AzMF.

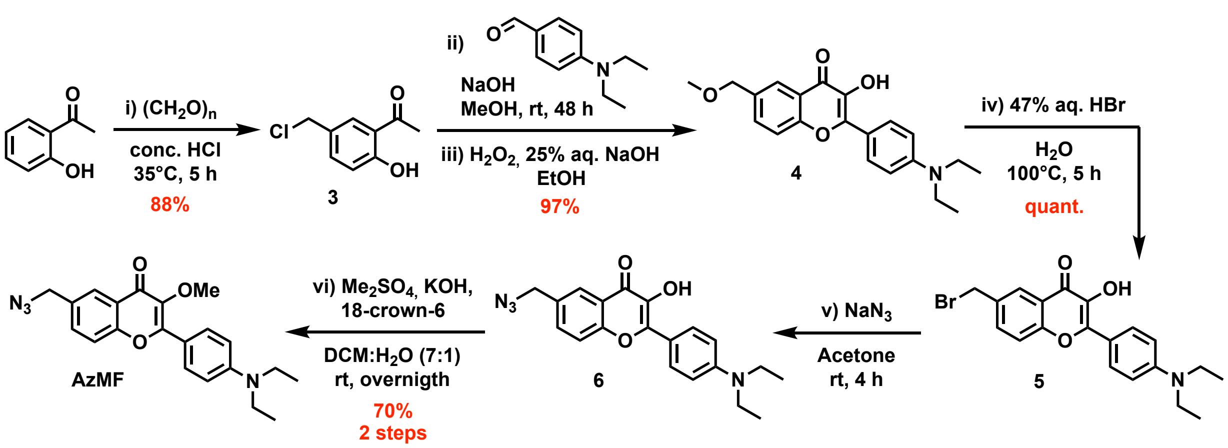

Scheme S3. Synthetic access to the charged dyes AlMF−, AlMF+−, and AzMF+.

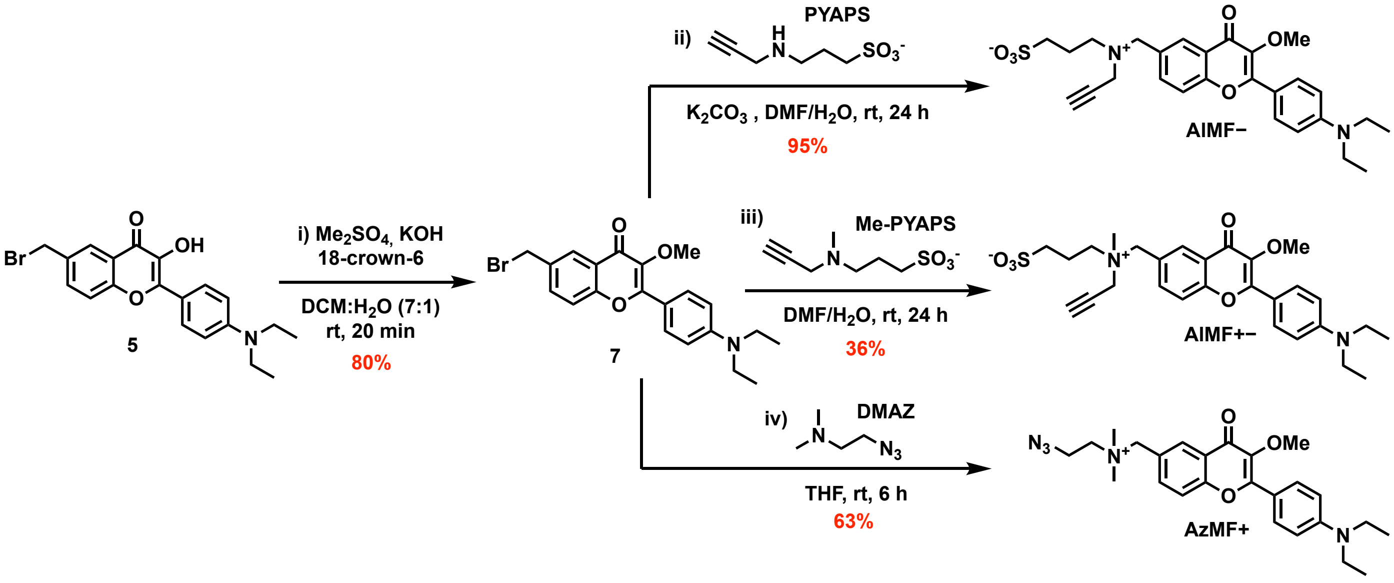

1.2 Preparation of the AlMF-based model nucleoside

Scheme S4. Synthetic access to AlMF-Nu.

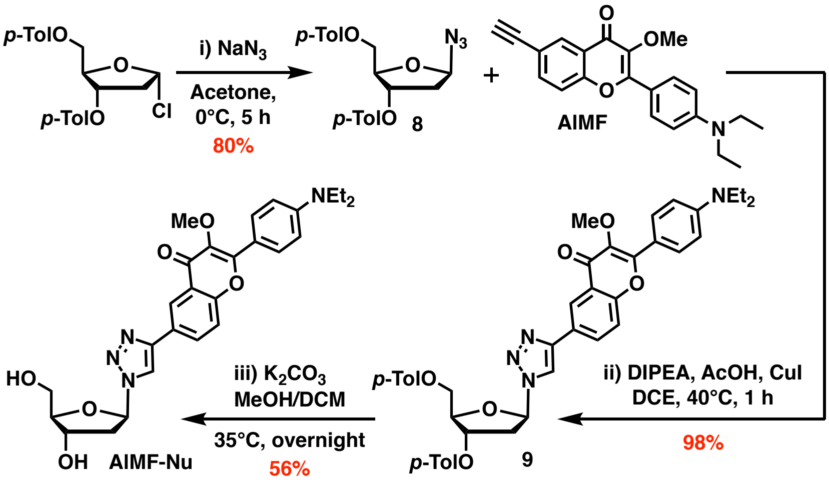

2. PHOTOPHYSICAL CHARACTERIZATION

2.1 Absorptivity determination

Due to a small amount of the reference **AlMF-Nu**, its molar extinction coefficient was not determined by the conventional weighting method, but by the NMR method, which is known to be more accurate. Thus, using vanillin as a reference, the concentration of the stock solution was accurately defined from the median area of several clearly resolved peaks. A series of dilutions in cascade allowed to calculate the corresponding absorptivity by UV–Vis spectroscopy (for all the considered dyes: $\varepsilon_{max} \simeq 41{,}000\ M^{-1}\cdot cm^{-1}$).

2.2 Steady-state fluorescence measurements of neutral labels

Table S1. Spectroscopic properties of AzMF, AlMF and its derived nucleoside analog (AlMF-Nu).

Solvent	$E_T(30)^a$	$\lambda_{abs}{}^b$			$\lambda_{em}{}^c$			Φ (%)^d		
		AlMF-Nu	AlMF	AzMF	AlMF-Nu	AlMF	AzMF	AlMF-Nu	AlMF	AzMF
**H_2O** [e]	63.1	417	410	413	550	550	550	0.4	0.4	0.3
**MeOH**	55.4	407	410	405	529	533	530	5	4.1	5
**EtOH**	51.9	406	408	404	522	524	520	38	30	38
**BuOH**	49.7	406	408	403	514	517	514	64	64	77
**CH_3CN**	45.6	395	397	394	504	509	509	68	62	64
**DMF**	43.2	nc.	402	397	nc.	505	501	nc.	73	86
**DMSO**	45.1	406	408	nc.	516	517	nc.	72	80	nc.
**CH_2Cl_2**	40.7	nc.	402	398	nc.	485	480	nc.	55	69
**EtOAc**	38.1	389	392	388	474	474	469	68	60	70
**THF**	36.2	391	395	390	466	479	471	75	81	86
**Toluene**	33.9	391	393	390	448	448	447	40	56	61
**Cyclohexane**	30.9	ns.	386	383	ns.	422	423	ns.	24	32

Footnotes: a) Normalized Reichardt's empirical solvent polarity index;[1] b) position of the absorption band maximum; c) position of the emission band maximum; d) quantum yield determined using *p*-dimethylaminoflavone (dMAF) in EtOH ($\Phi = 0.27$) as a reference[2]; e) due to the lack of solubility of the considered fluorophores, potential H-aggregates are likely to have formed in water.

[1] C. Reichardt, *Chem. Rev.* **1994**, 94, 2319–2358.
[2] S. M. Ormson, R. G. Brown, F. Vollmer and W. Rettig, *J. Photochem. Photobiol. A* **1994**, *81*, 65–72.

2.3 pK$_A$ study

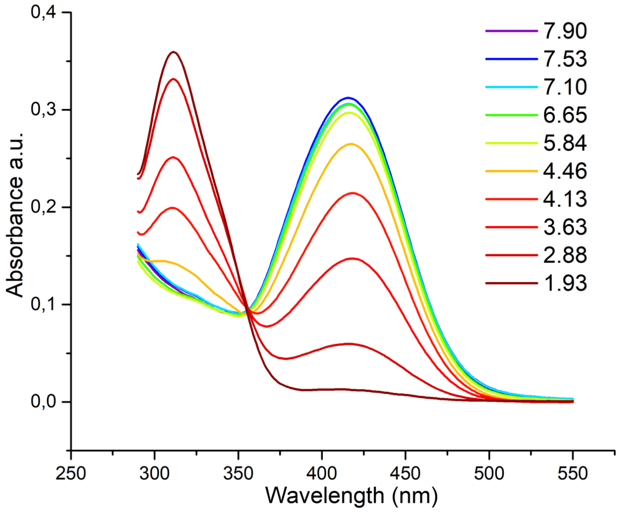

Figure S1. pK$_A$ study of AlMF.

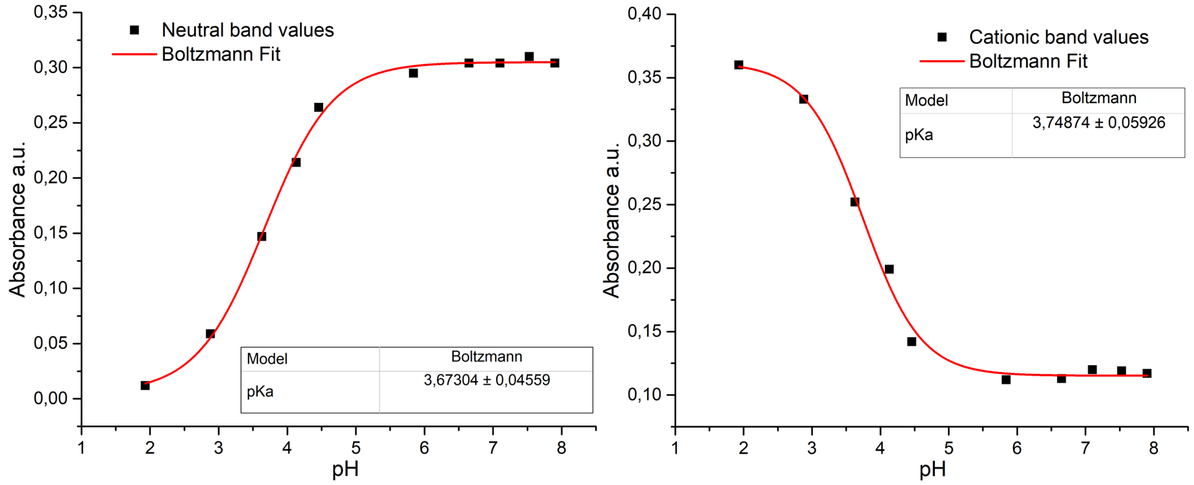

Figure S2. pK$_A$ titration curves of AlMF.

2.4 Photobleaching studies

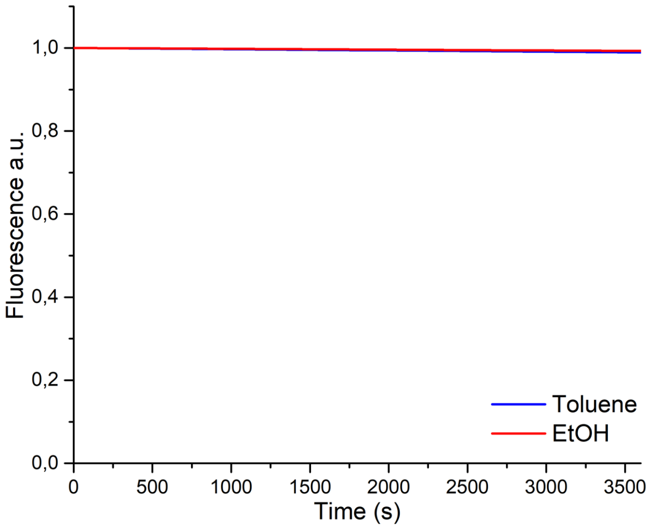

Figure S3. Photobleaching decays of AlMF.

Footnotes: To a 2μM solution of dye in a cuvette, with an 8x8 aperture slit, the evolution of fluorescence intensity was monitored over 1 hour. The fluorescence is recorded at its maximum intensity (448 nm in toluene, 522 nm in EtOH) with an excitation wavelength corresponding to the absorption maximum in the considered solvent (391 nm in toluene, 406 nm in EtOH).

2.5 Hydration study

Table S2. Hydration study of the neutral and charged fluorophores.

H_2O (%) in THF	λ_{abs}[a]				λ_{em}[b]				Φ (%)[c]			
	AlMF	AlMF −	AzMF +	AlMF +−	AlMF	AlMF −	AzMF +	AlMF +−	AlMF	AlMF −	AzMF +	AlMF +−
**0**	390	388	396	396	463	473	484	482	78	49	59	41
**1**	391	/	/	/	479	/	/	/	84	/	/	/
**2**	392	389	401	399	487	487	507	504	91	59	53	63
**4**	394	390	405	400	497	495	519	513	85	57	41	69
**6**	395	/	/	/	503	/	/	/	74	/	/	/
**8**	397	391	408	403	508	501	525	520	65	46	20	29
**10**	398	/	/	/	510	/	/	/	55	/	/	/
**15**	400	394	410	405	514	508	530	526	42	31	11	15
**20**	401	/	/	/	516	/	/	/	36	/	/	/
**30**	404	/	/	/	520	/	/	/	23	/	/	/
**40**	407	401	415	412	524	517	536	531	18	14	5	6
**50**	410	/	/	/	527	/	/	/	13	/	/	/
**60**	414	408	420	417	529	524	542	538	9	7	3	4
**80**	ns	416	428	426	ns	533	552	548	ns	2	2	2
**90**	ns	417	426	425	ns	538	551	553	ns	1	1	1
**100**	ns	413<br>421[d]	422<br>428[d]	421<br>432[d]	ns	556	564	563	ns	>1	>1	>1

Footnotes: a) Position of the absorption band maximum; b) position of the emission band maximum; c) quantum yield determined using *p*-dimethylaminoflavone (dMAF) in EtOH (Φ = 0.27) as a reference;[2] d) value extracted from the excitation spectrum at the maximum absorption wavelength. *ns stands for not soluble.*

Figure S4. Hydration study of AlMF−: normalized absorption (left) and fluorescence (right) spectra.

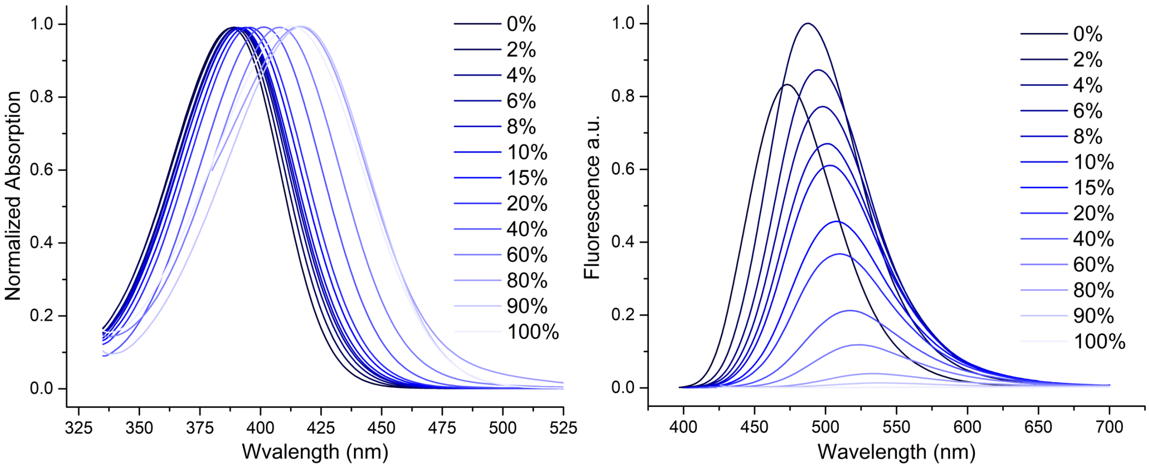

Footnotes: Titration performed by adding water to a THF solution containing the dye. A different solution was done for each percentage of water to keep the same concentration for each experiment (2 μM).

Figure S5. Hydration study of AzMF+: normalized absorption (left) and fluorescence (right) spectra.

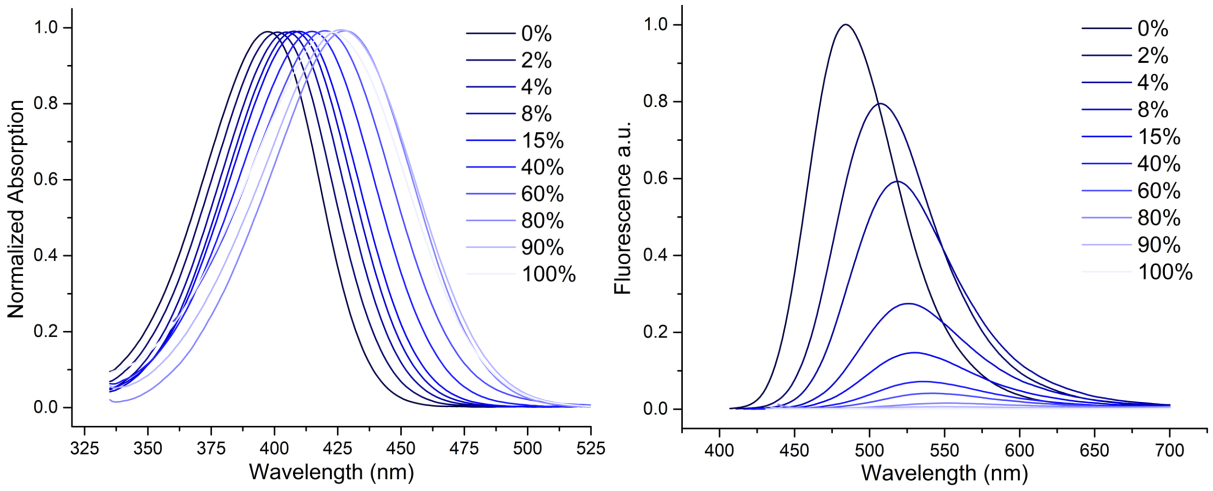

Figure S6. Hydration study of AlMF+−: normalized absorption (left) and fluorescence (right) spectra.

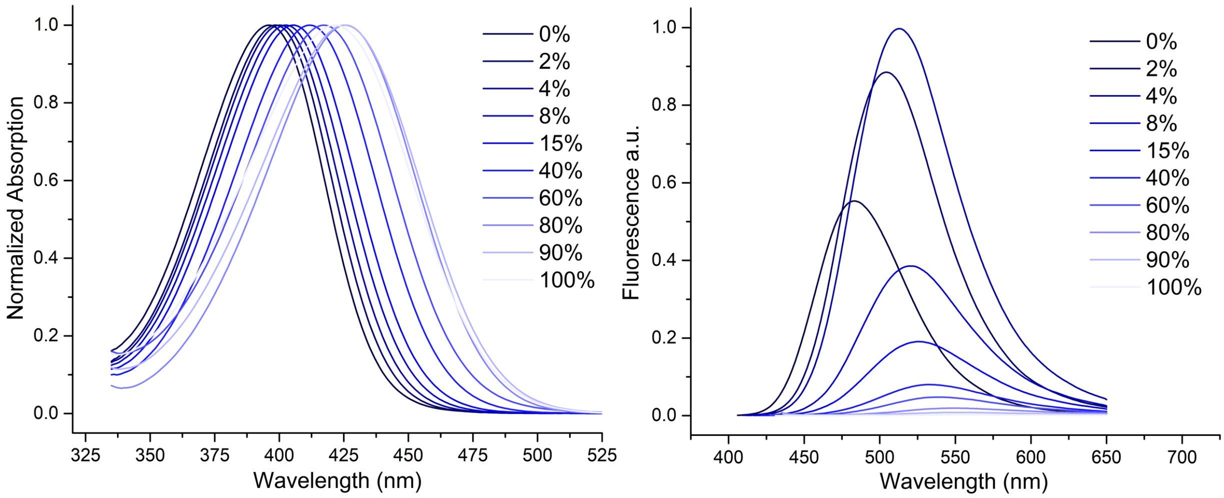

2.6 Steady-state fluorescence measurements of charged labels

Table S3. Spectroscopic properties of the charged analogs AlMF−, AzMF+ and AlMF+−.

Solvent	$E_T(30)$[a]	λ_{abs}[b]			λ_{em}[c]			Φ (%)[d]		
		AlMF −	AzMF +	AlMF +−	AlMF −	AzMF +	AlMF +−	AlMF −	AzMF +	AlMF +−
**H_2O**	63.1	421 ↑[e]	428 ↑	432 ↑	556 =	564 ↑	563 ↑	>1 =	>1 =	>1 =
**MeOH**	55.4	402 =	414 ↑	412 ↑	524 =	543 ↑	539 ↑	10 ↑	2 ↓	3 ↓
**BuOH**	49.7	400 =	417 ↑	412 ↑	505 ↓	533 ↑	529 ↑	72 =	30 ↓↓	43 ↓↓
**CH_3CN**	45.6	392 =	401 ↑	400 ↑	494 ↓	516 ↑	515 ↑	63 =	58 ↓	59 ↓
**DMSO**	45.1	400 ↓	409 =	407 =	510 ↓	527 ↑	525 ↑	91 ↑↑	61 ↓	59 ↓
**THF**	36.2	389 =	397 ↑	397 ↑	475 ↑	487 ↑	487 ↑	55 ↓↓	54 ↓	45 ↓↓
**Toluene**	33.9	396 =	402 ↑	404 ↑	459 ↑	464 ↑	462 ↑	41 =	40 =	7 ↓↓

Footnotes: a) Reichardt's empirical solvent polarity index;[1] b) position of the absorption band maximum; c) position of the emission band maximum; d) quantum yield determined using *p*-dimethylaminoflavone (dMAF) in EtOH (Φ = 0.27) as a reference;[2] e) arrows and equals refer to an increase, decrease or similarity in the value of the neutral dyes **AlMF** or **AzMF**.

2.7 Absorption & emission spectra of charged labels

Figure S7. Absorbance (left) & fluorescence (right) spectra of AlMF−.

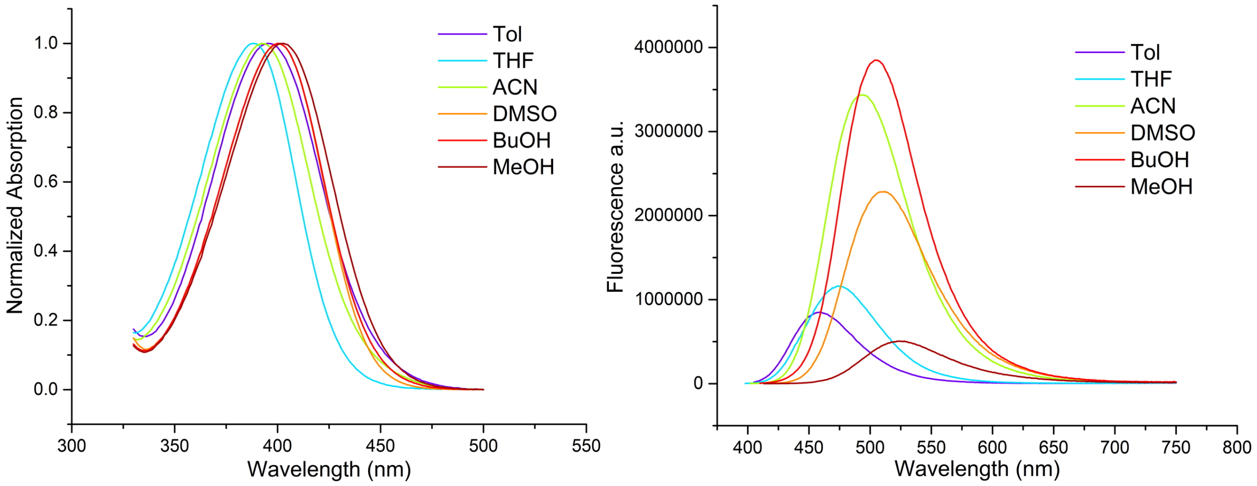

Figure S8. Absorbance (left) & fluorescence (right) spectra of AzMF+.

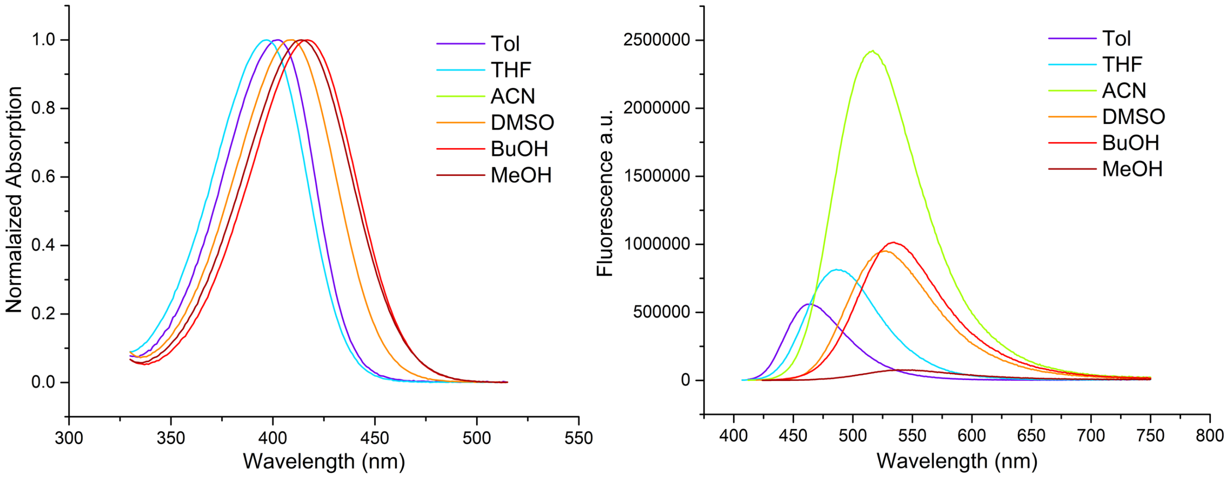

Figure S9. Absorbance (left) & fluorescence (right) spectra of AlMF+−.

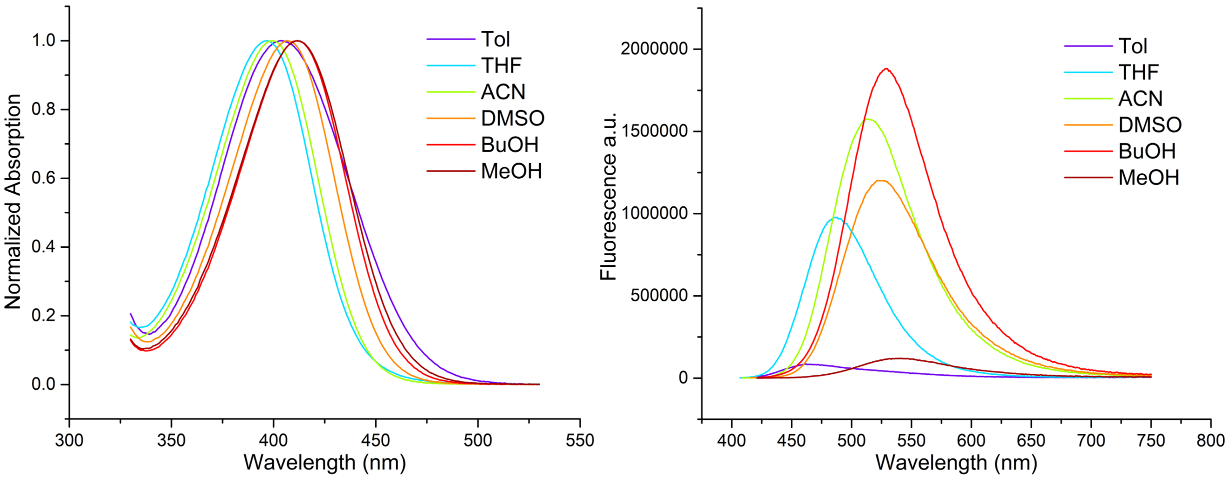

3. SPECTROSCOPIC STUDIES OF MODEL ODNs

3.1 ODN synthesis and purification

General method:

Wild-type and clickable ODNs were purchased from Microsynth AG. ODNs were ordered purified and ready to use.

Typical labeling procedure:

First, a 5mM aq. solution of $CuSO_4 \cdot 5H_2O$ and the BTTES ligand is prepared.

In a 200-µL vial, were sequentially added the ODN sequence (0.2mM aq. solution, 50 μL, 10 nmol, 1 eq.), DMSO (20 μL), dye (5 mM in DMSO, 10 μL, 50 nmol, 5 eq.), sodium ascorbate (5mM aq. solution, 10 μL, 50 nmol, 5 eq.), and finally the $CuSO_4$/BTTES mixture (5mM aq. solution, 10 μL, 50 nmol, 5 eq.). The mixture was vortexed overnight at rt. The solution was then recovered, and the vial was washed with minimal of H_2O and DMSO. Thus, the whole mixture was then purified by RP-HPLC.
ODNs were analyzed (0.5 mL/min) and purified (2.0 mL/min) by RP-HPLC (HPLC apparatus: Waters™ 600 Controller with Waters™ 996 Photodiode Array Detector. Columns: analytical, 300 × 4.60 mm, 5μm particle size, Clarity® 100Å, Phenomenex®; semi-preparative, Clarity® 5u Oligo-RP column 250 x 10 mm Phenomenex®).

Figure S10. Representative 2D (top) and 3D (bottom) HPLC profiles of a crude mixture after post-synthetic click labeling reaction at two different wavelengths (260 & 430 nm).

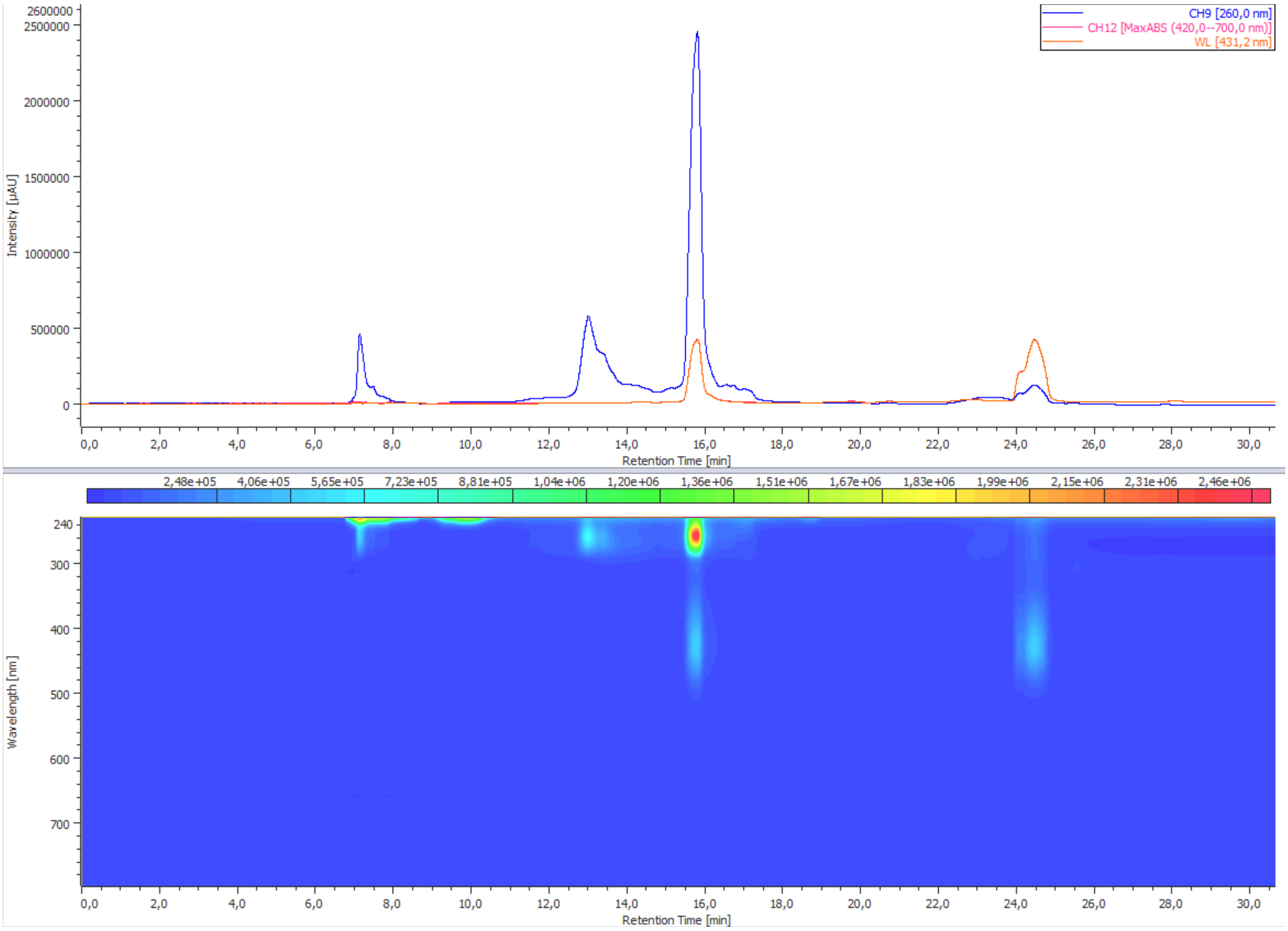

Gradient: 100% A for 2 min → 40% A: 60% B during 10 min then keep it for 2 min, →10% A: 90 % B during 2 min then keep it for 14 min. A = Buffer pH 7.0 (90% TEAB buffer 100 mM:10% CH_3CN) and B = 90% CH_3CN:10% Buffer A. Peak at 8 min: starting clickable ODN; peak at 15 min: clicked ODN; peak at 24 min: starting clickable label.

Figure S11. Representative 2D HPLC profiles of purified ODN sequences: TXT labeled with AlMF (left) and AWA labeled with AzMF (right)

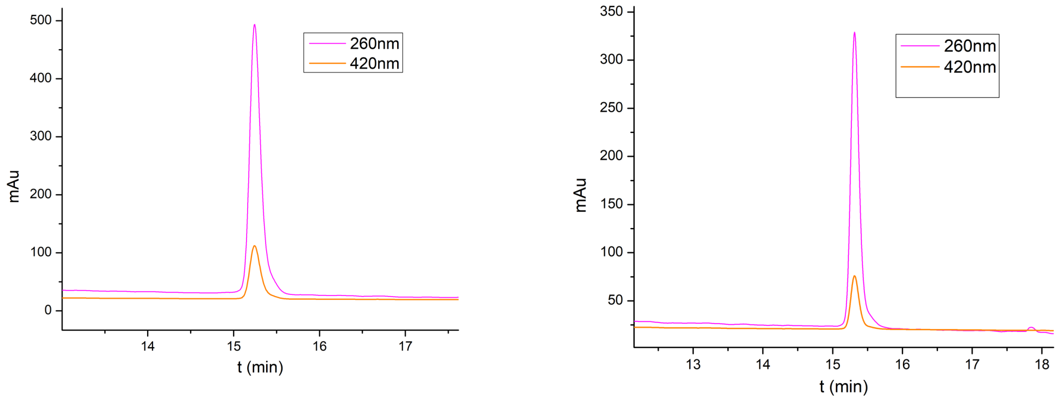

Table S4. Nature, absorptivity, and linker chemical structure of the ODNs to be labeled.

Sequence	Extinction coefficient (L·mol^{-1}·cm^{-1})	Linker
5′-**YCAG** TCG CTC GCT GAC-3′	133,100	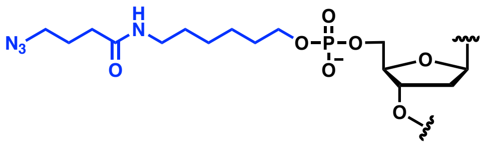
5′-**YGCA** AAA TTT AAA ACG-3′	158,100	
5′-CAG TCG **CXC** GCT GAC-3′	133,100	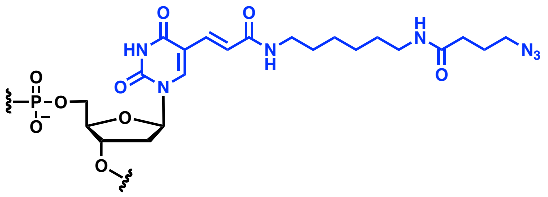
5′-GCA AAA **TXT** AAA ACG-3′	157,400	
5′-GCA AAA AAA **AXA** AAA AAA ACG-3′	238,600	
5′-GCA AAA **TXsT** AAA ACG-3′	157,400	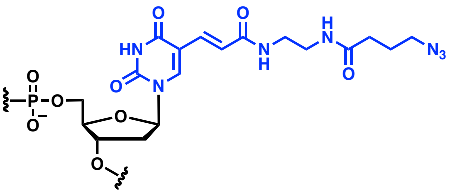
5′-**VGCA** AAA TTT AAA ACG-3′	158,100	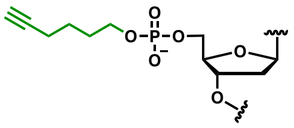
5′-GCA AAA **TZT** AAA ACG-3′	157,400	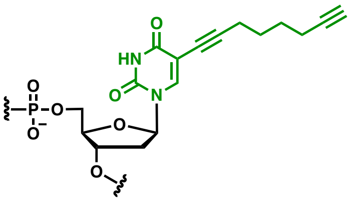
5′-GCA AAA **TWT** AAA ACG-3′	149,600	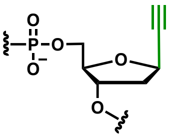
5′-GCA AAA AAA **AWA** AAA AAA ACG-3′	255,800	

3.2 HRMS analysis of labeled ODNs

Table S5. Mass of the single-stranded DNA tagged with AlMF.

ODN	Sequence	HRMS found (calc.) [M+H]$^+$
		Labeled with **AlMF**
**YCAG**	5´-**YCAG** TCG CTC GCT GAC-3´	5165.0580 (5165.0597)
**YGCA**	5´-**YGCA** AAA TTT AAA ACG-3´	5229.1165 (5229.1148)
**CXC**	5´-CAG TCG **CXC** GCT GAC-3´	5140.1009 (5140.0992)
**TXT**	5´-GCA AAA **TXT** AAA ACG-3´	5204.1559 (5204.1543)
**AXA**	5´-GCA AAA AAA **AXA** AAA AAA ACG-3´	6753.3779 (6753.3751)
**TXsT**	5´-GCA AAA **TXsT** AAA ACG-3´	5148.0899 (5148.0917)

Table S6. Mass of the single-stranded DNA tagged with AzMF.

ODN	Sequence	HRMS found (calc.) [M+H]$^+$
		Labeled with **AzMF**
**VGCA**	5´-**VGCA** AAA TTT AAA ACG-3´	5131.1129 (5131.1152)
**TZT**	5´-GCA AAA **TZT** AAA ACG-3´	5061.1361 (5061.1333)
**TWT**	5´-GCA AAA **TWT** AAA ACG-3´	4871.0616 (4871.0590)
**AWA**	5´-GCA AAA AAA **AWA** AAA AAA ACG-3´	6767.4341 (6767.4359)

3.3 Temperature-induced denaturation studies

Preparation of the ODN duplex solution: In a 500-μL cuvette, were sequentially added the clicked ss-ODN probe solution (4μM aq. solution, 250 μL) and its complementary wild-type ss-ODN solution (200μM aq. solution, 5 μL), and PBS solution (250 μL, [Na] = 300 mM, [P] = 25 mM). Melting curves were monitored by following the temperature-dependence of the absorbance changes at 260 nm of the sample (2μM concentration of each strand). Absorption spectra were recorded in a Peltier-thermostatted cell holder on a Cary 100 Bio UV–Vis spectrophotometer (Varian/Agilent) using Suprasil® quartz cuvettes with 1-cm path length. The temperature range for denaturation measurement was 20–75 °C. Speed of heating was 0.3 °C/min.

Table S7. Melting temperatures of duplexes labeled with AlMF.

Duplex	T_m (°C)		
	**AlMF**	*Wild Type*[a]	ΔT_m **AlMF** (°C)[b]
**YCAG**·*GTC*	69.9	65.8 [61.6]	+ 4.1
**YGCA**·*CGT*	47.8	48.1 [45.9]	− 0.3
**CXC**·*GAG*	58.8	65.8 [61.6]	− 7.0
**TXT**·*AAA*	44.6	48.1 [45.9]	− 3.5
**TXsT**·*AAA*	43.2	48.1 [45.9]	− 4.9

[a] T_m of the corresponding duplex formed from unmodified ODNs and its theorical values given in square brackets. [b] ΔT_m refers to the difference of T_m between the labeled and wild type ODNs.

Table S8. Melting temperatures of duplexes labeled with AzMF+, AlMF+−, & AlMF−.

Duplex	T_m (°C)						
	**AzMF+**	**AlMF+−**	**AlMF−**	*Wild Type*[a]	ΔT_m **AzMF+** (°C)[b]	ΔT_m **AlMF+−** (°C)[b]	ΔT_m **AlMF−** (°C)[b]
**TXT**·*AAA*	/	40.3	38.9	48.1	/	− 7.8	− 9.2
**VGCA**·*CGT*	47.0	/	/	48.1	− 1.1	/	/
**TWT**·*AAA*	34.3	/	/	48.1	− 13.8	/	/
**TZT**·*AAA*	41.7	/	/	48.1	− 6.4	/	/

[a] T_m of the corresponding duplex formed from unmodified ODNs and its theorical values given in square brackets. [b] ΔT_m refers to the difference of T_m between the labeled and wild type ODNs.

Figure S12. Melting temperature curves of duplexes tagged with AlMF.

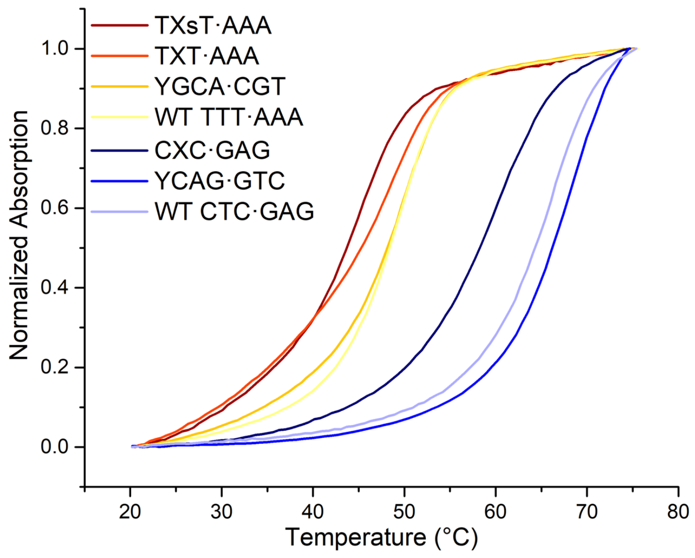

Figure S13. Melting temperature curves of duplexes tagged with AzMF+.

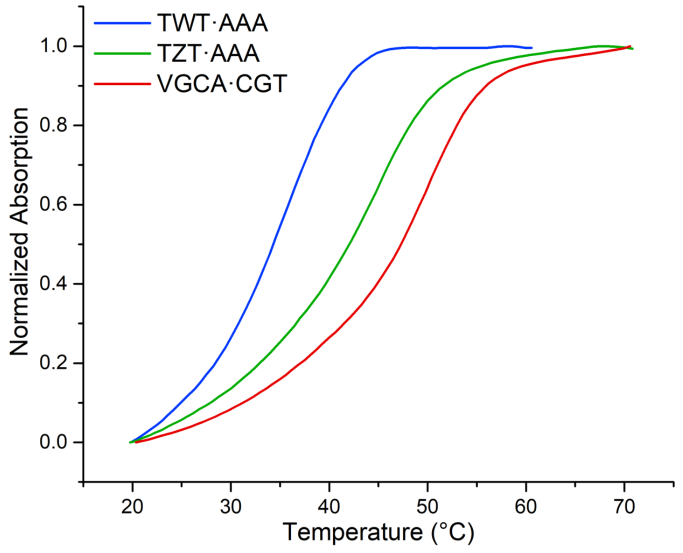

Figure S14. Melting temperature curves of duplexes tagged with AlMF− (left) & AlMF+− (right).

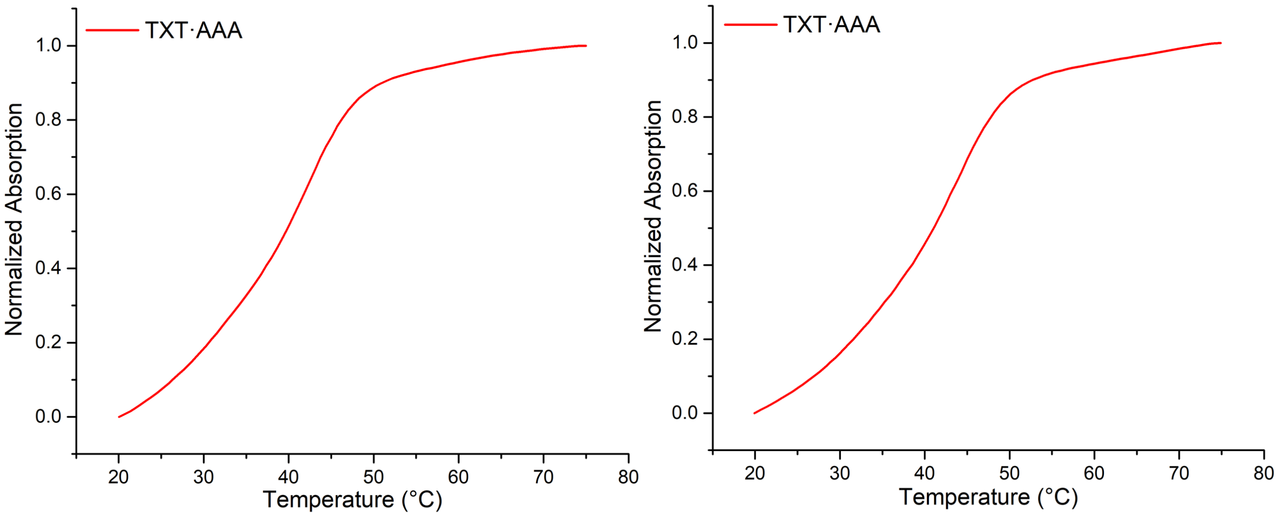

3.4 Steady-state fluorescence measurements

Absorption and fluorescence experiments were performed in duplicate in pH 7.0 phosphate-buffered saline (12 mM PBS, 120 mM NaCl). Absorption spectra were recorded at 25°C on a Cary 100 Bio UV–Vis spectrophotometer (Varian/Agilent) using Suprasil® quartz cuvettes with 1-cm path length. Fluorescence spectra were recorded on a FluoroMax 4.0 spectrofluorometer (Jobin Yvon, Horiba) with a 2x2 aperture slit and were corrected at excitation and emission. Measured solutions were prepared with absorbance of about 0.05 at 25 °C at the excitation wavelength mentioned in the corresponding experiments. Quantum yields were corrected according to the variation of the refractive index of the different solvents and were determined using *p*-dimethylaminoflavone (dMAF) in EtOH (λ_{ex} = 404 nm, Φ = 0.27) as a standard reference.[2]

Table S9. Spectroscopic properties of ODNs labeled with AzMF+, AlMF+−, & AlMF−.

Sequence	λ_{abs} (nm)[a]			λ_{em} (nm)[b]			Φ (%)[c]		
	AzMF+	AlMF+−	AlMF−	AzMF+	AlMF+−	AlMF−	AzMF+	AlMF+−	AlMF−
TXT	/	436	430	/	550	539	/	11	11
TXT·*AAA*	/	439	438	/	551	540	/	19	18
YGCA	/	442	431	/	551	541	/	9	10
YGCA·*CGT*	/	437	428	/	549	543	/	5	7
TWT	441	/	/	555	/	/	7	/	/
TWT·*AAA*	442	/	/	543	/	/	16	/	/
VGCA	436	/	/	550	/	/	9	/	/
VGCA·*CGT*	431	/	/	550	/	/	5	/	/

[a] Position of the absorption band maximum. [b] Position of the emission band maximum. [c] Quantum yield determined using *p*-dimethylaminoflavone (dMAF) in EtOH (Φ = 0.27).[2]

Table S10. Spectroscopic properties of AXA and AXA·TAT labeled with AlMF.

λ_{ex} (nm)[a]	AXA	AXA · *TAT*	Absorptivity ratio[c]	Fluorescence intensity ratio[d]
	Brightness (L.mol^{-1}.cm^{-1})[b]			
440	4510	13940	1.08	3.4
450	3583	13263	1.20	3.8
460	2730	11430	1.35	4.3
470	1814	8777	1.57	5.0
488	651	4237	2.1	6.3

[a] Screening of the excitation wavelength. [b] Brightness calculation: absorptivity at the excitation wavelength*Quantum Yield; [c] Absorbance of ds/Absorbance of ss; [d] Fluorescence intensity of ds/Fluorescence intensity of ss.

3.5 Absorbance & fluorescence spectra

Figure S15. Absorption (left) and emission (right) spectra of ss- and ds-ODNs labeled with AzMF+.

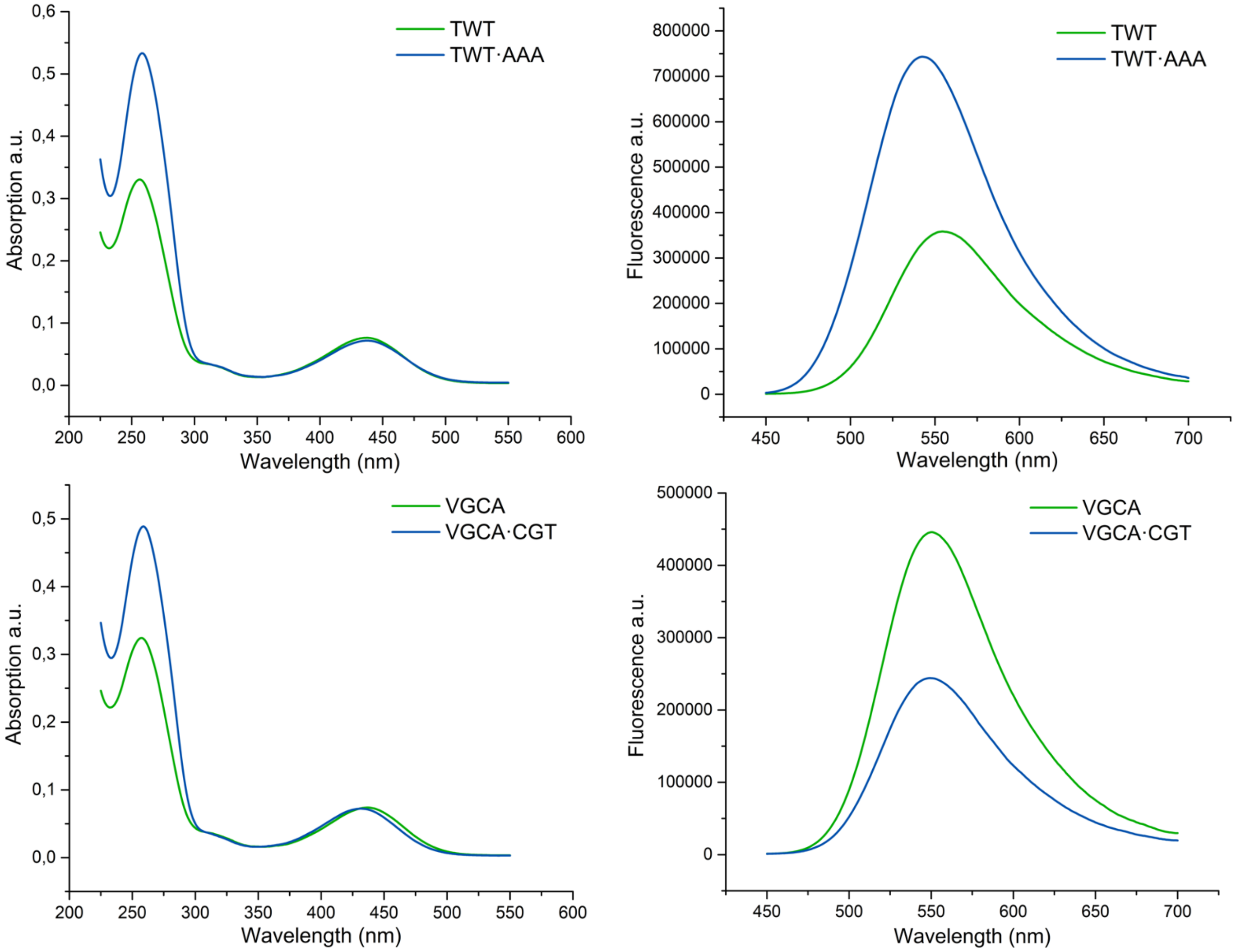

Figure S16. Absorption (left) and emission (right) spectra of ss- and ds-ODNs labeled with AlMF+−.

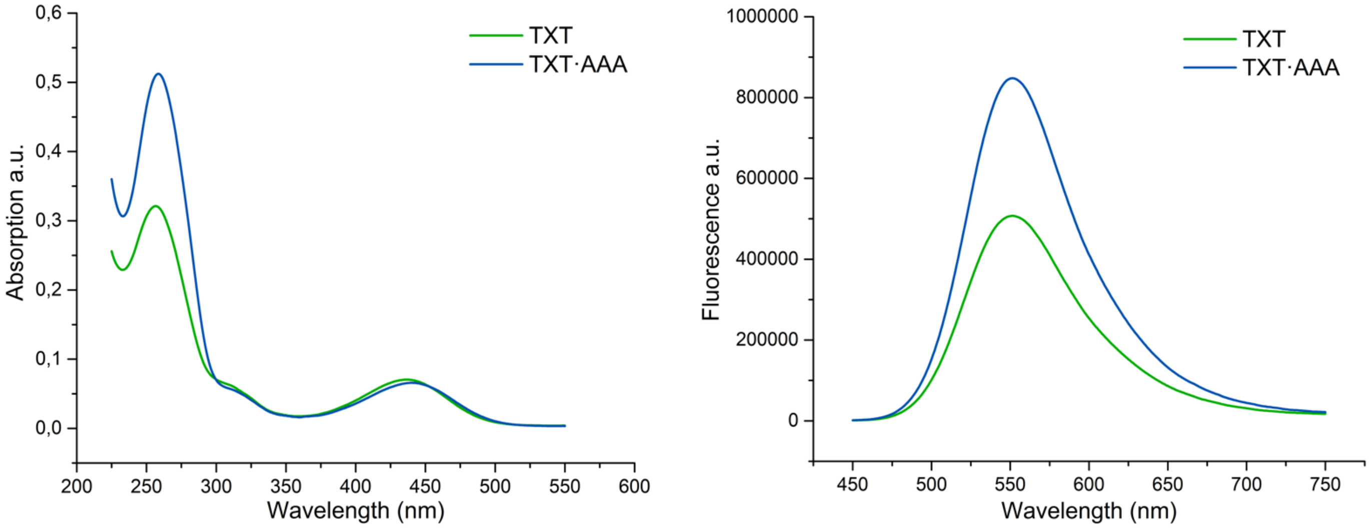

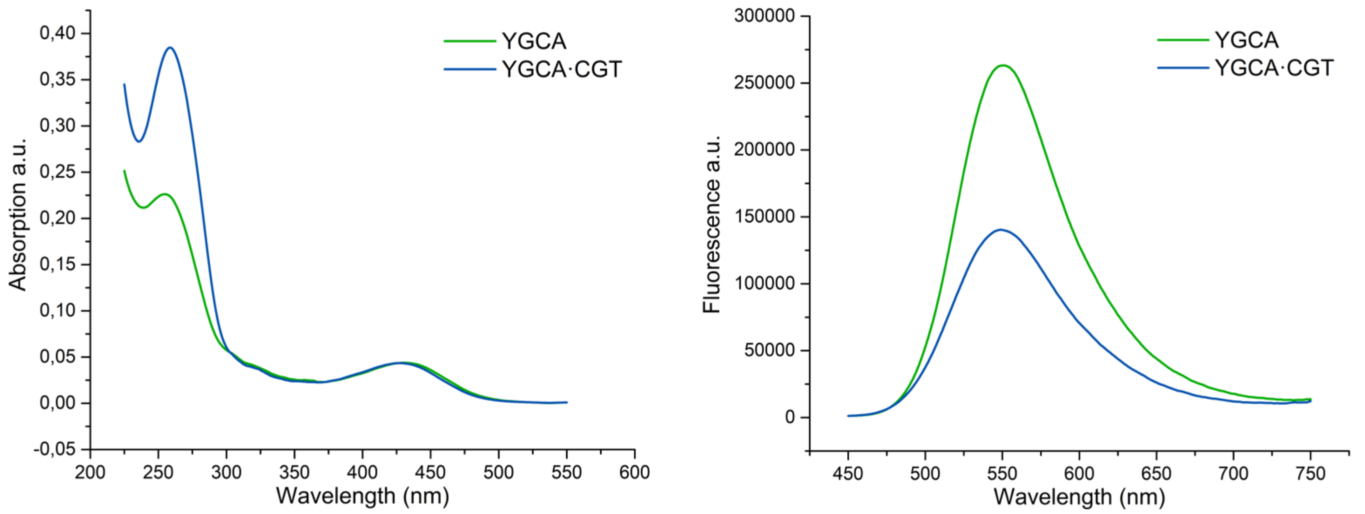

Figure S17. Absorption (left) and emission (right) spectra of ss- and ds-ODNs labeled with AlMF−.

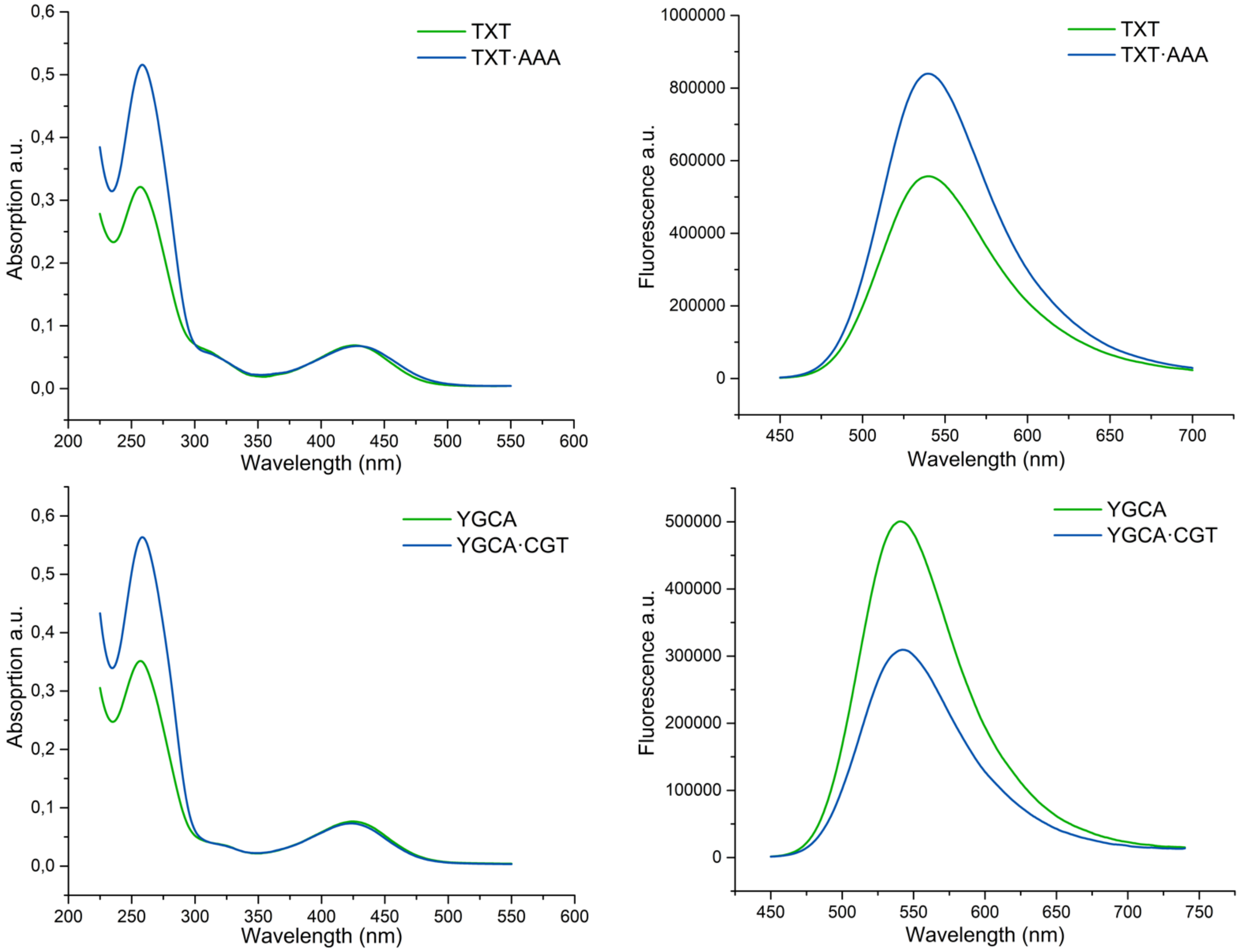

Figure S18. Absorption (left) and emission (right) spectra of ss- and ds-ODNs labeled with AlMF.

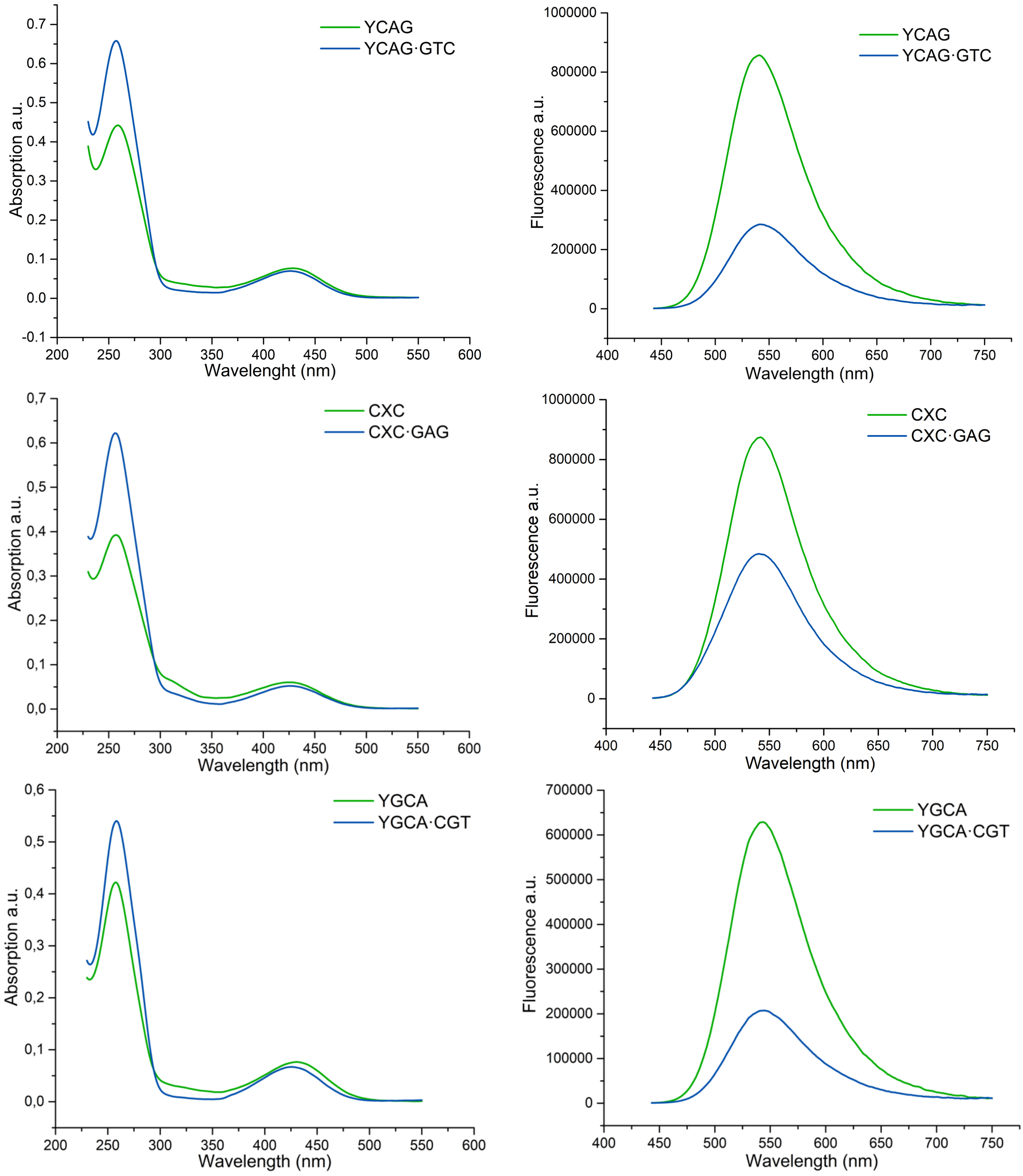

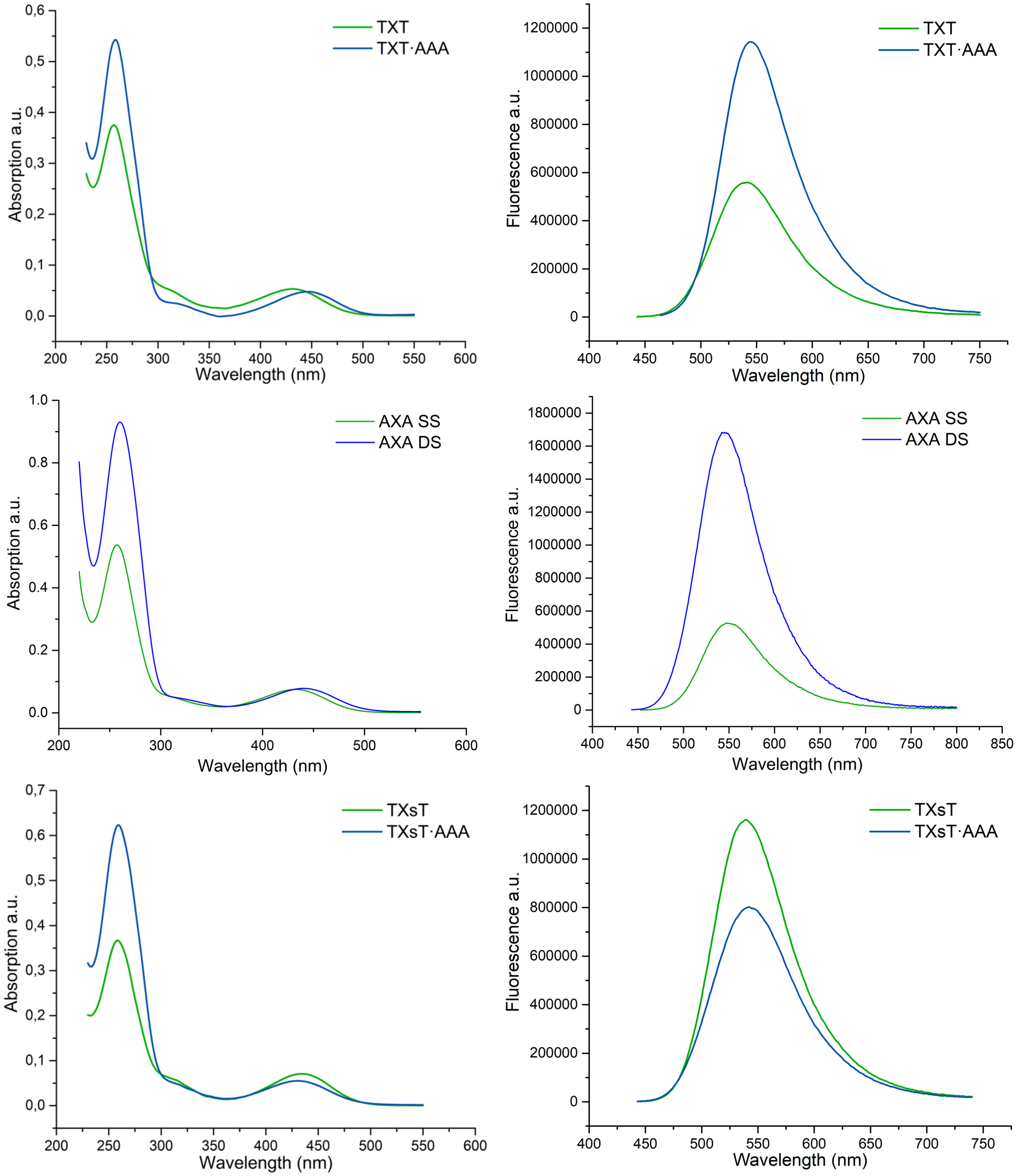

TXT
TXT·AAA
Absorption a.u.
Wavelength (nm)
Fluorescence a.u.
AXA SS
AXA DS
TXsT
TXsT·AAA

Figure S19. Absorption (left) and emission (right) spectra of ss- and ds-ODNs labeled with AzMF.

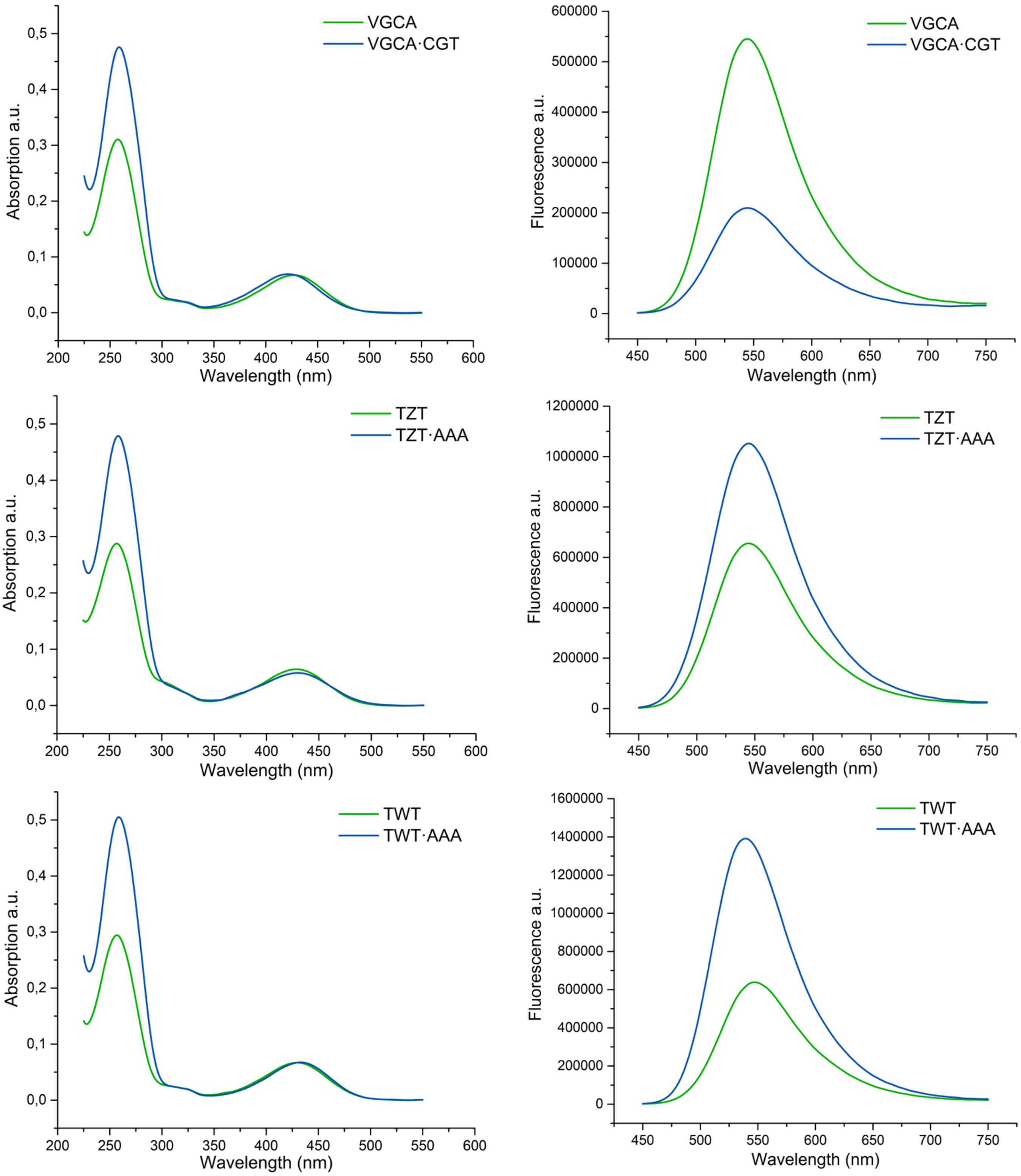

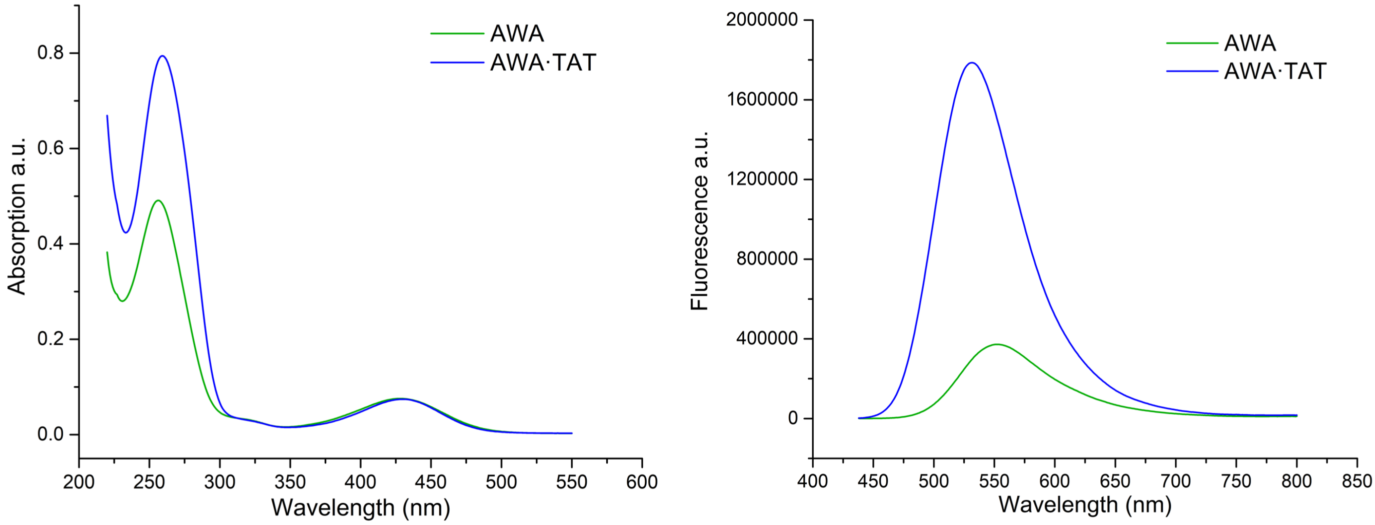

Figure S20. Fluorescence spectra of TXT and TXT·AAA labeled with AlMF at different excitation wavelengths.

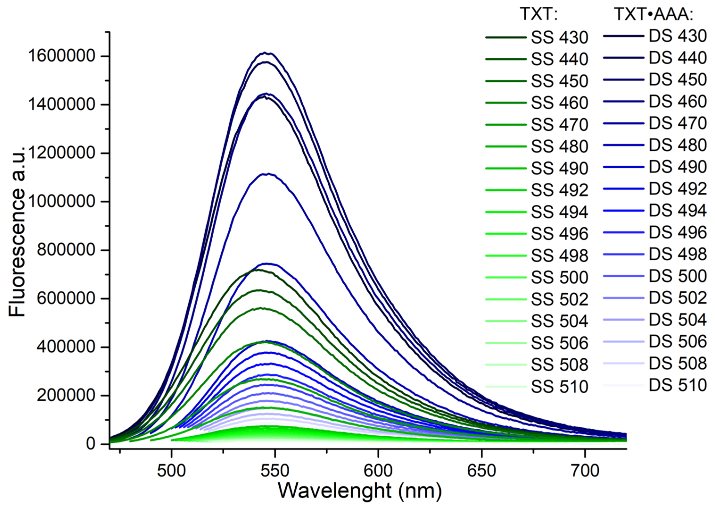

Footnotes: Fluorescence spectra obtained by screening the excitation wavelength *(values reported in Table 3)*.

Figure S21. Fluorescence spectra of AXA and AXA·TAT labeled with AlMF at different excitation wavelengths.

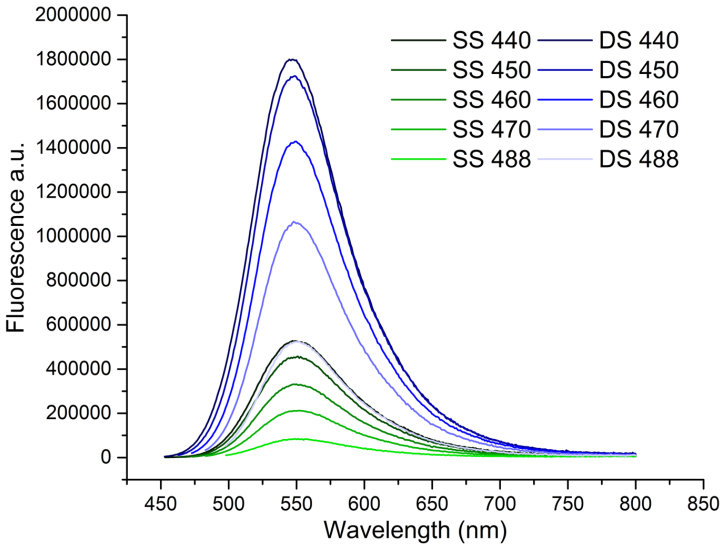

Footnotes: Fluorescence spectra obtained by screening the excitation wavelength *(values reported in Table S10)*.

NMR Spectra

Figure S22. ^{1}H- & ^{13}C-NMR spectra of 1: 6-bromo-2-(4-(diethylamino)phenyl)-3-hydroxy-4H-chromen-4-one.

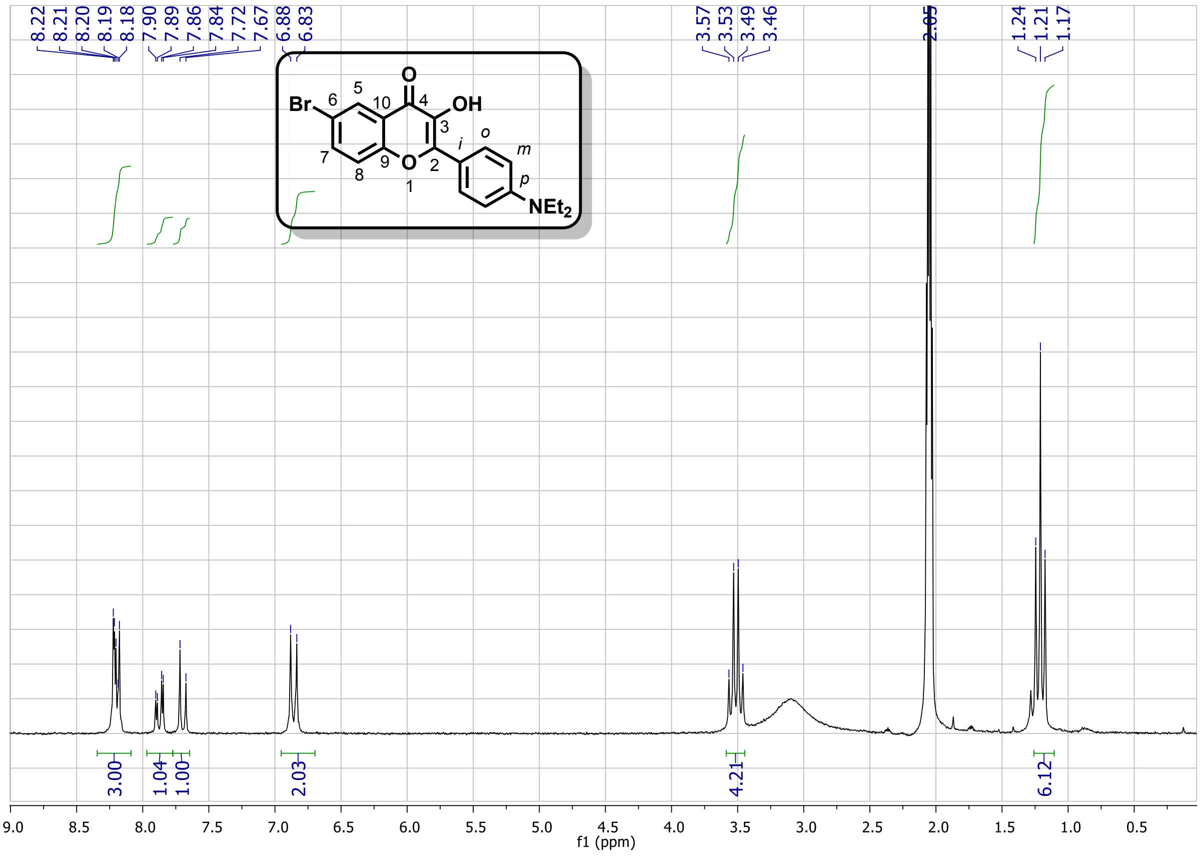

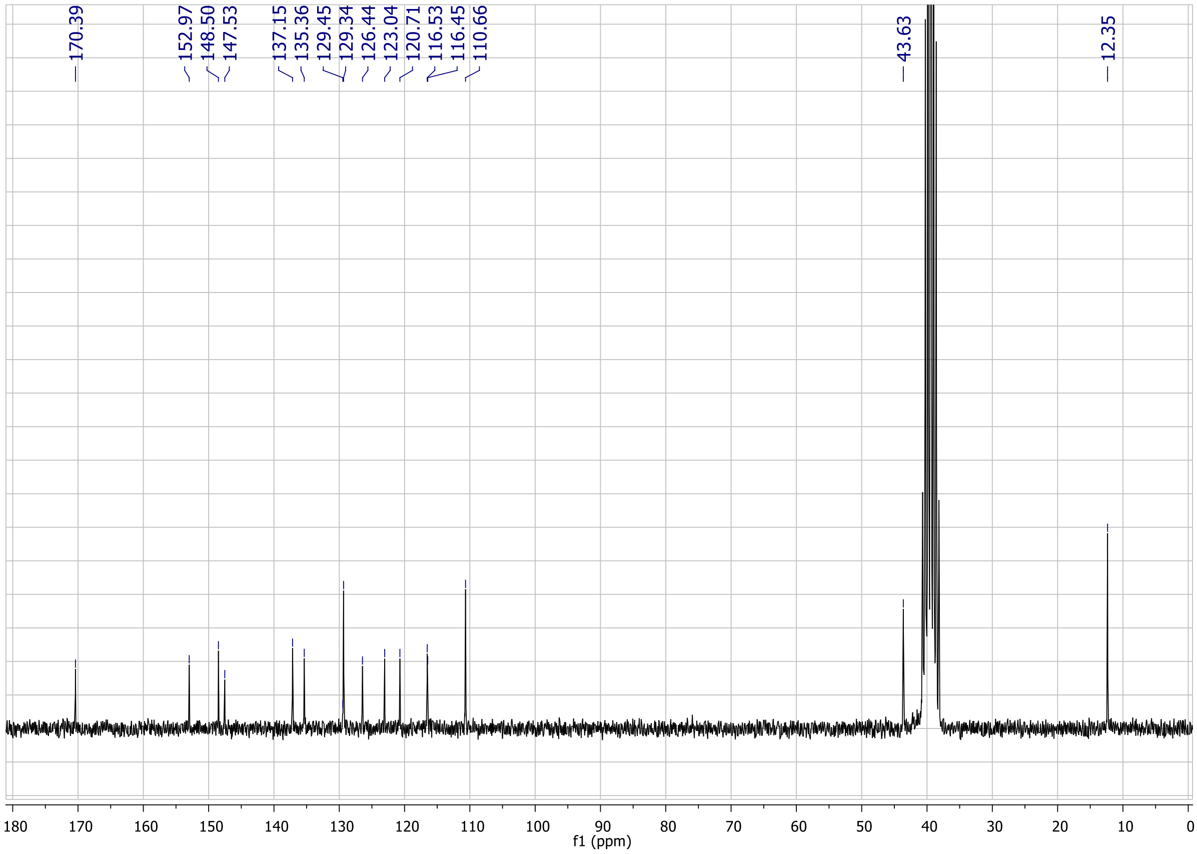

Figure S23. ***^{1}H- & ^{13}C-NMR spectra of 2: 6-bromo-2-(4-(diethylamino)phenyl)-3-methoxy-4H-chromen-4-one.***

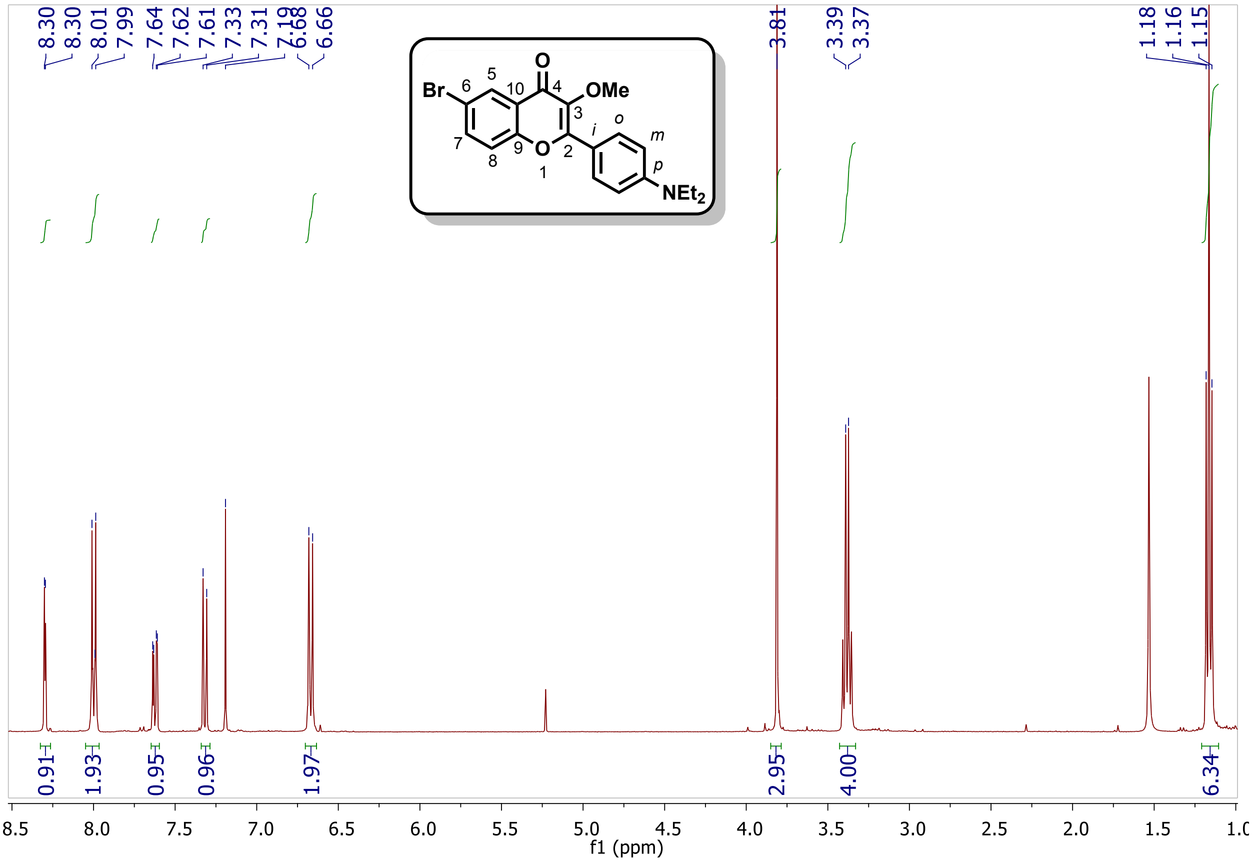

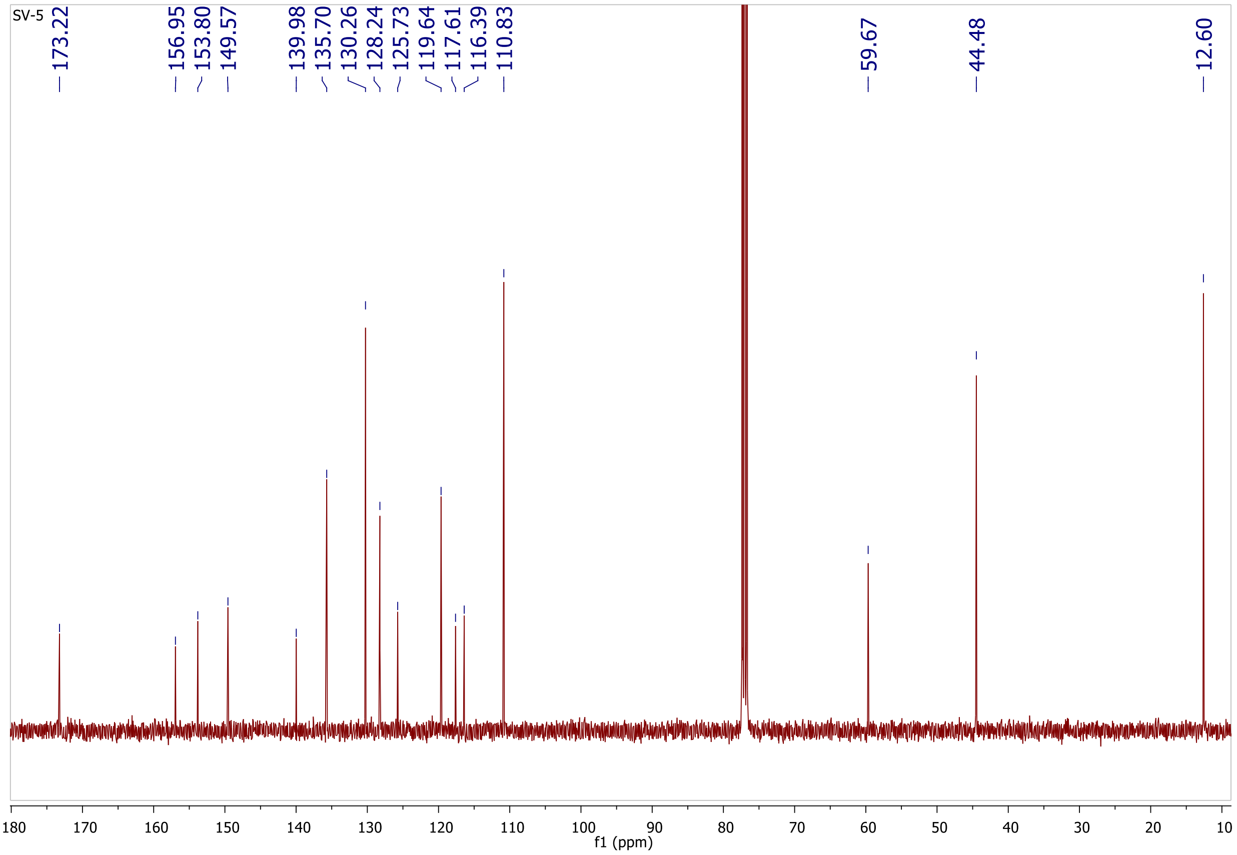

***Figure S24.** ^{1}H- & ^{13}C-NMR spectra of AlMF: 2-(4-(diethylamino)phenyl)-6-ethynyl-3-methoxy-4H-chromen-4-one.*

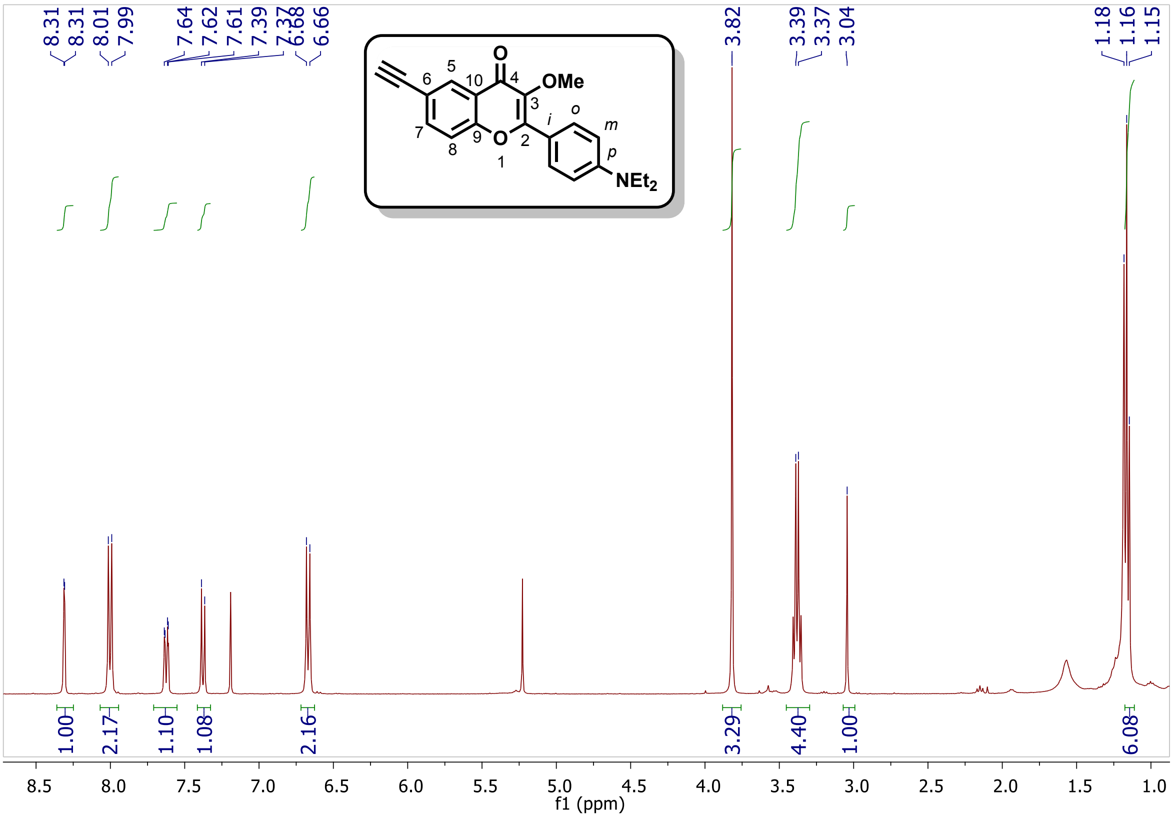

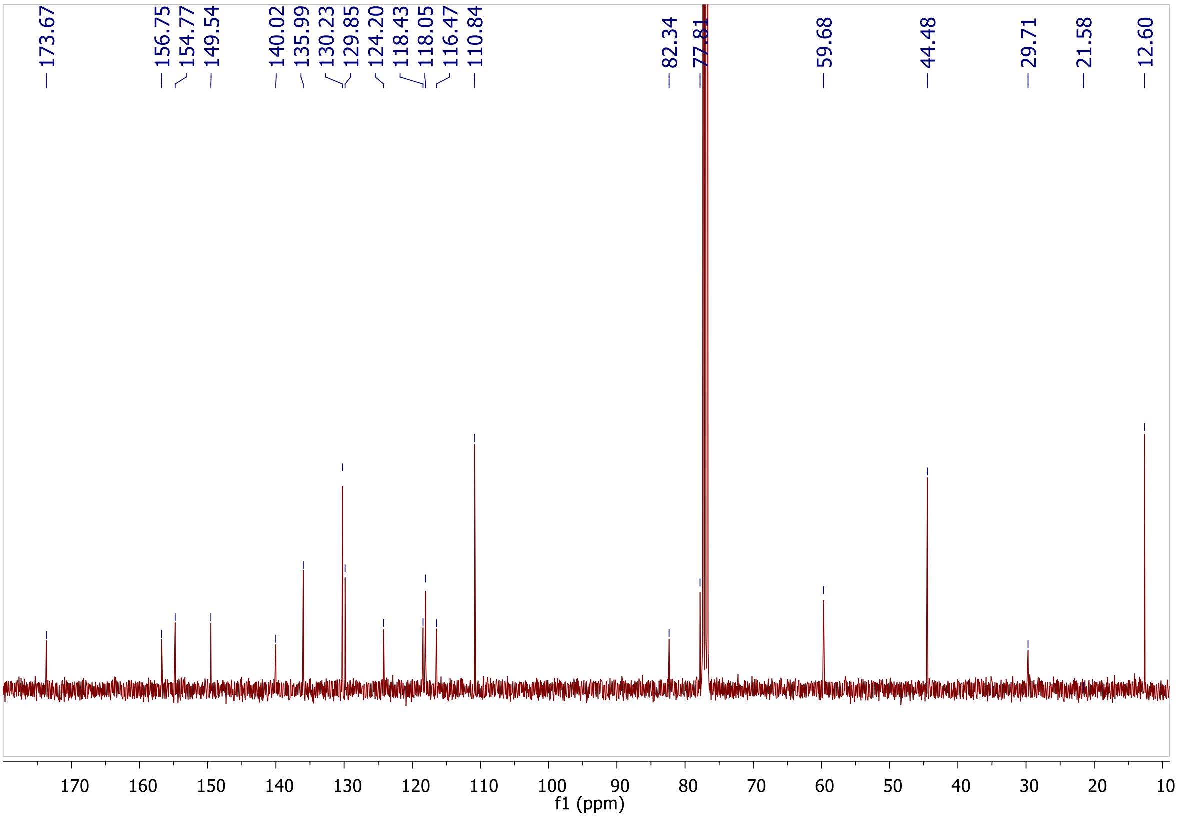

Figure S25. ^{1}H- & ^{13}C-NMR spectra of 3: 1-(5-(chloromethyl)-2-hydroxyphenyl)ethan-1-one.

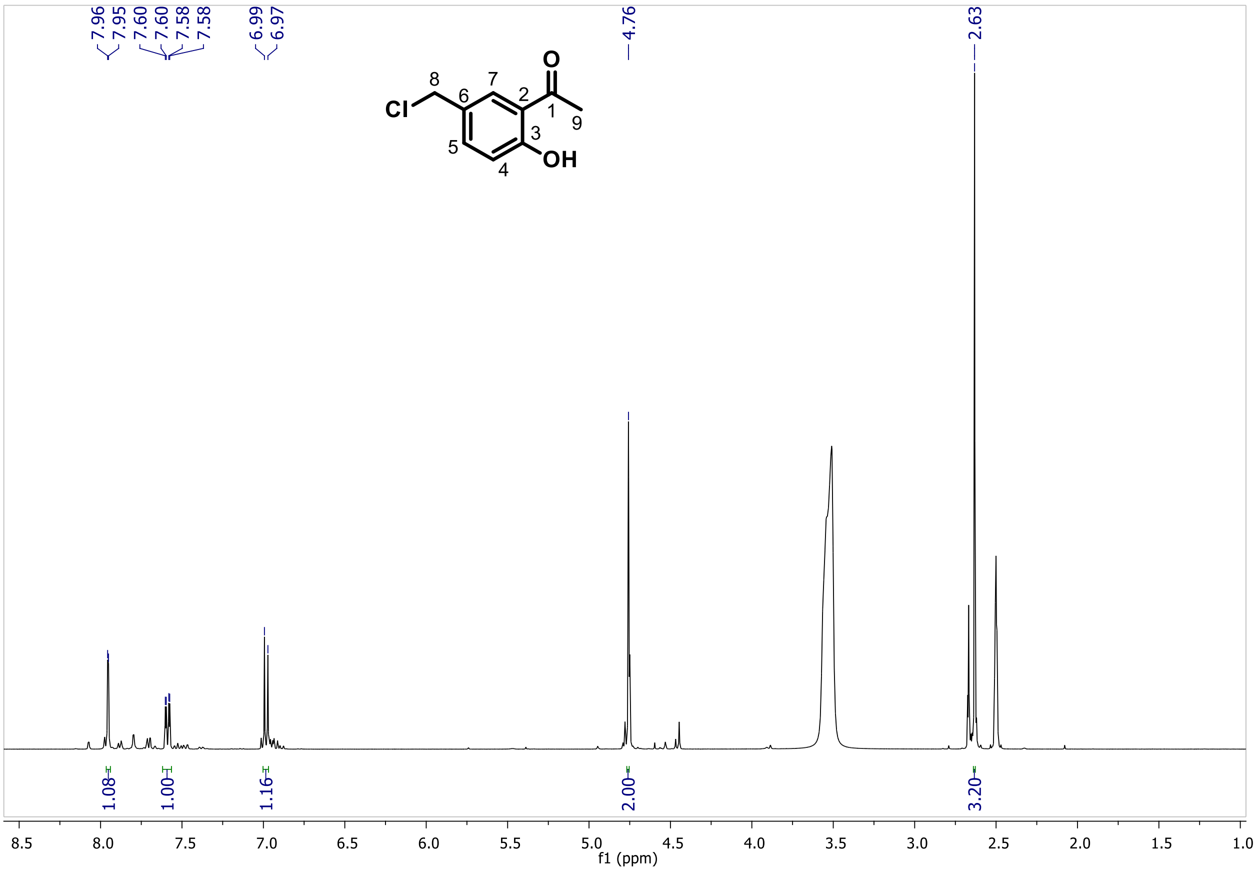

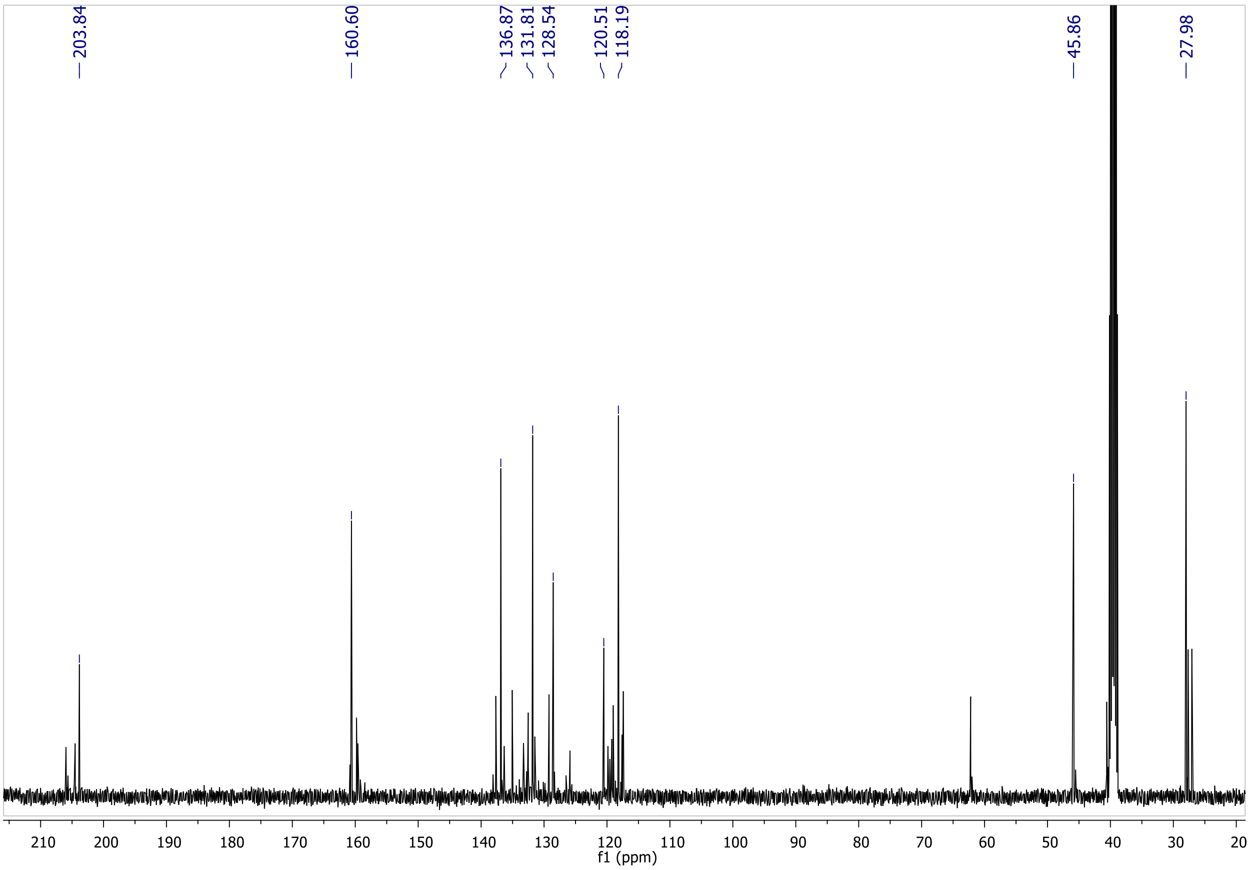

Figure S26. ***^{1}H-NMR spectrum of 4: 2-(4-(diethylamino)phenyl)-3-hydroxy-6-(methoxymethyl)-4H-chromen-4-one.***

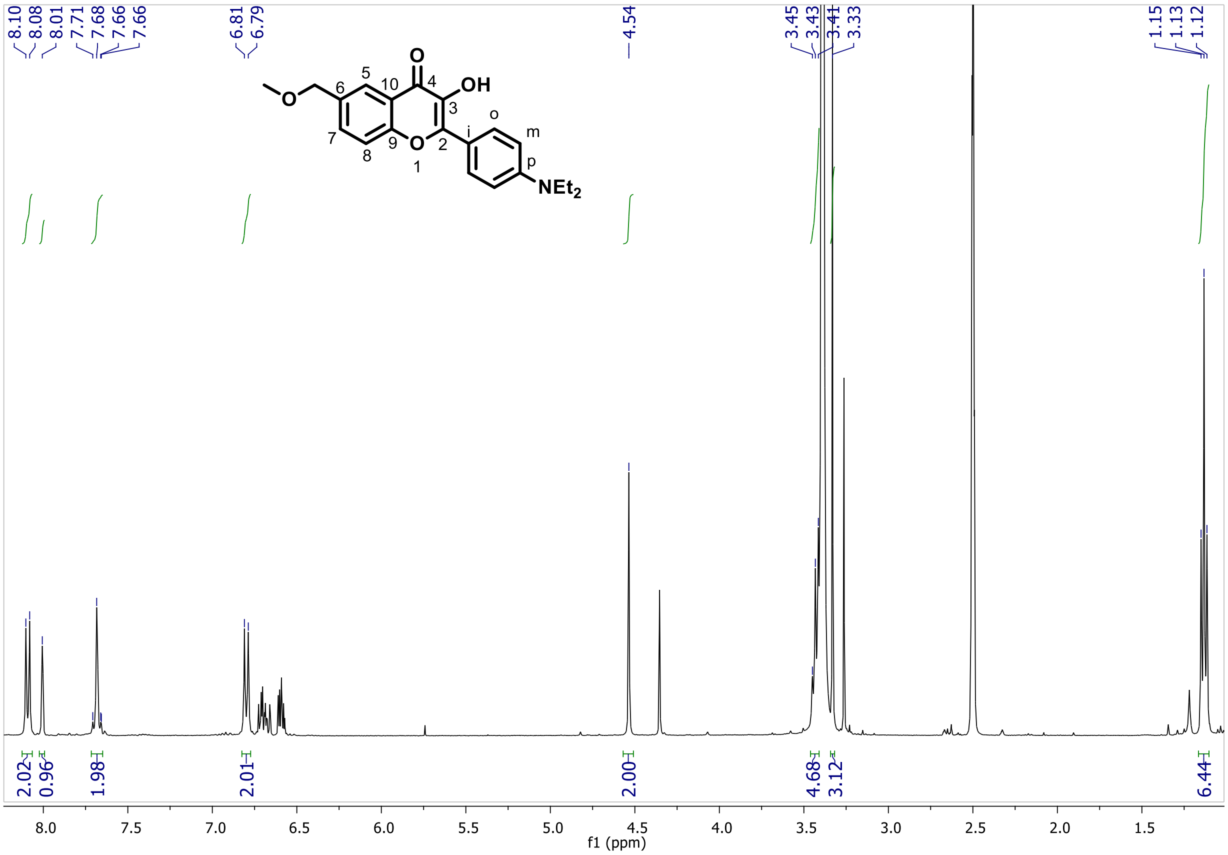

Figure S27. ***^{1}H-NMR spectrum of 5: 6-(bromomethyl)-2-(4-(diethylamino)phenyl)-3-hydroxy-4H-chromen-4-one.***

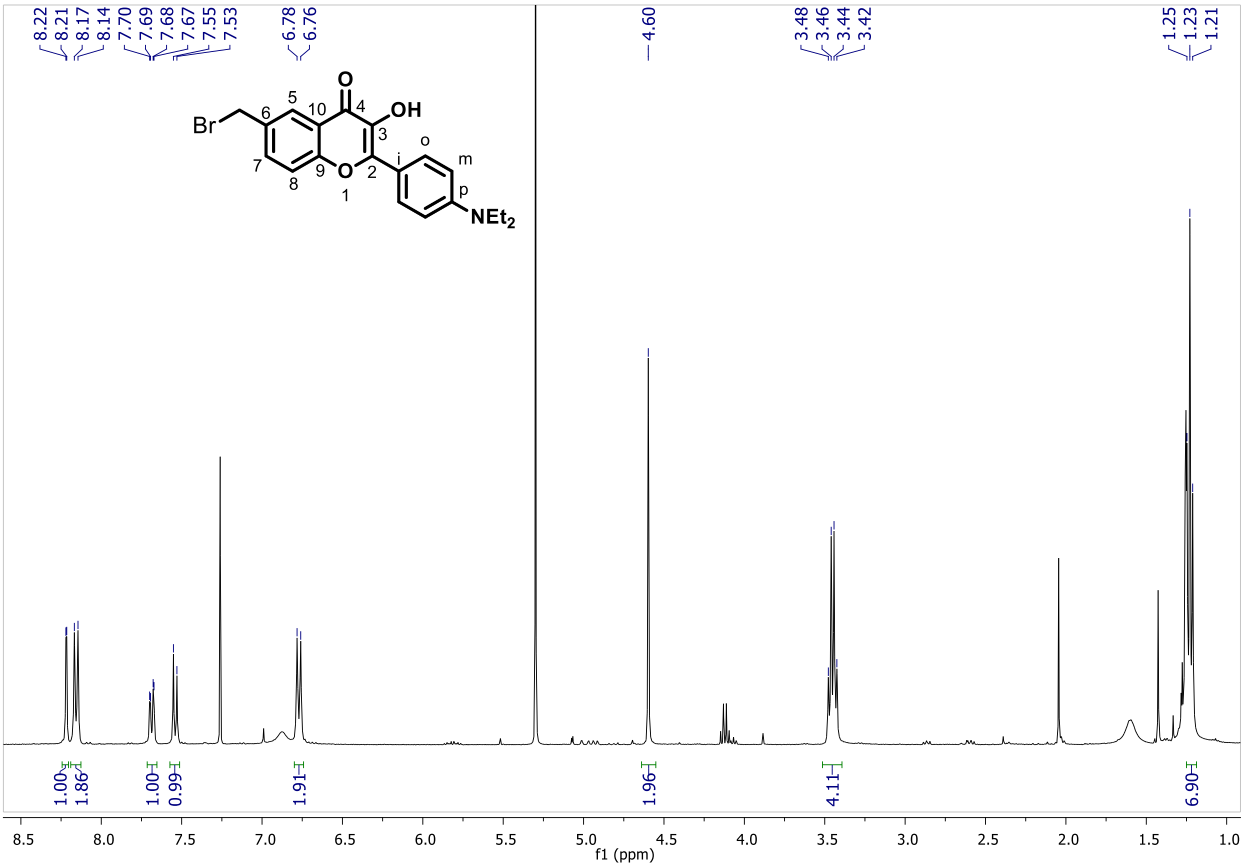

Figure S28. ^{1}H-, ^{13}C-, COSY-, & HSQC-NMR spectra of AzMF: 6-(azidomethyl)-2-(4-(diethylamino)phenyl)-3-methoxy-4H-chromen-4-one.

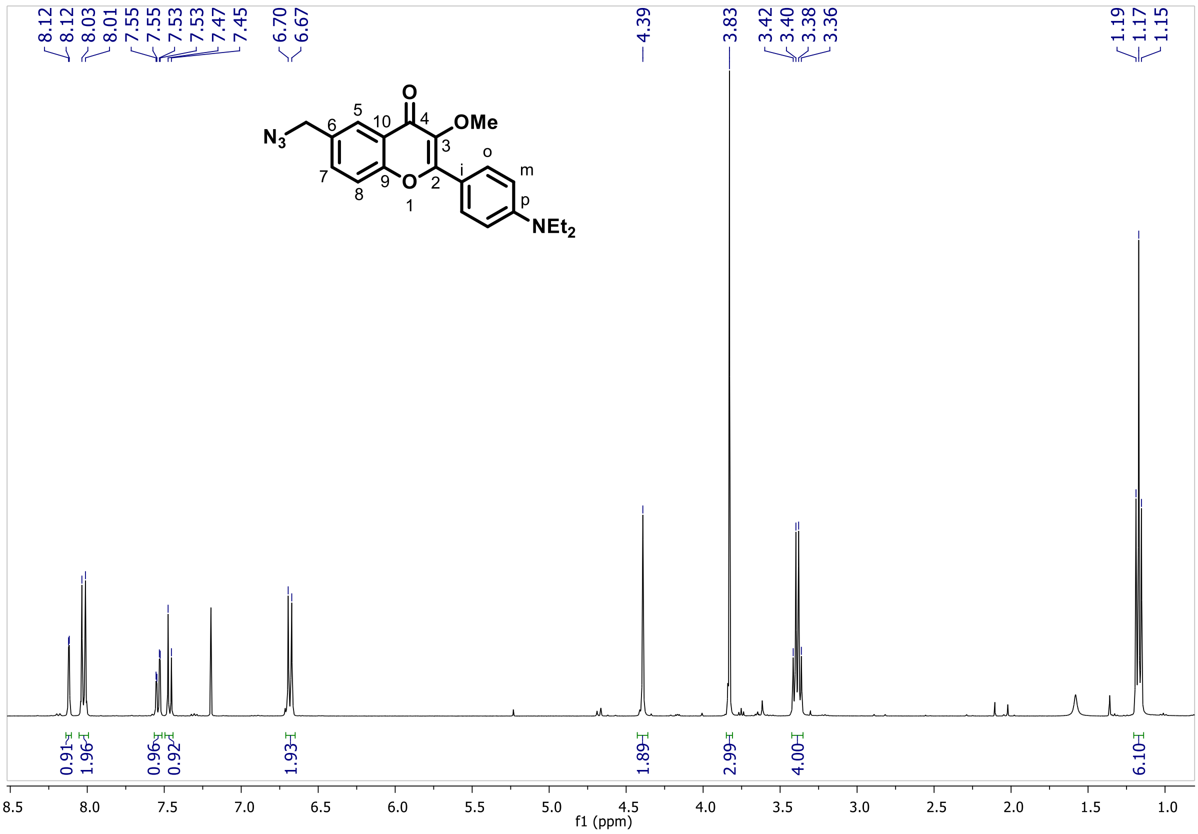

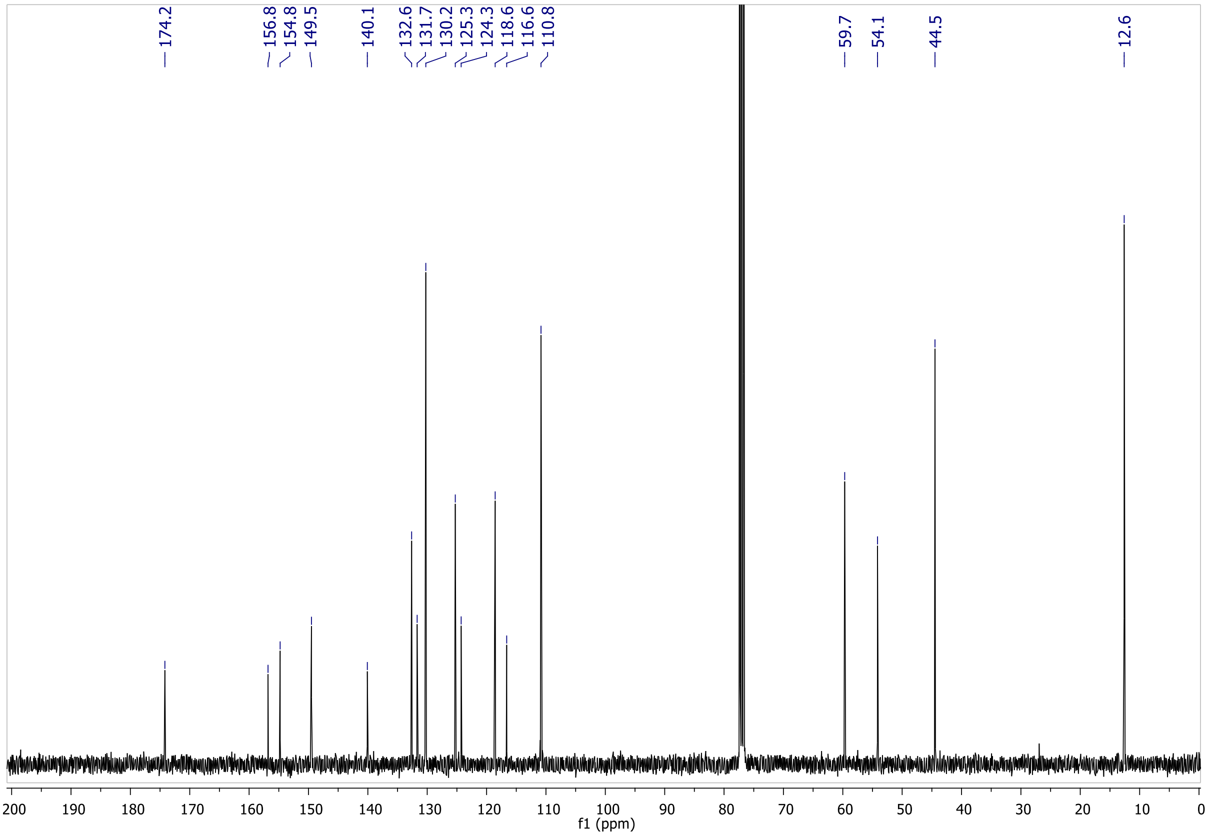

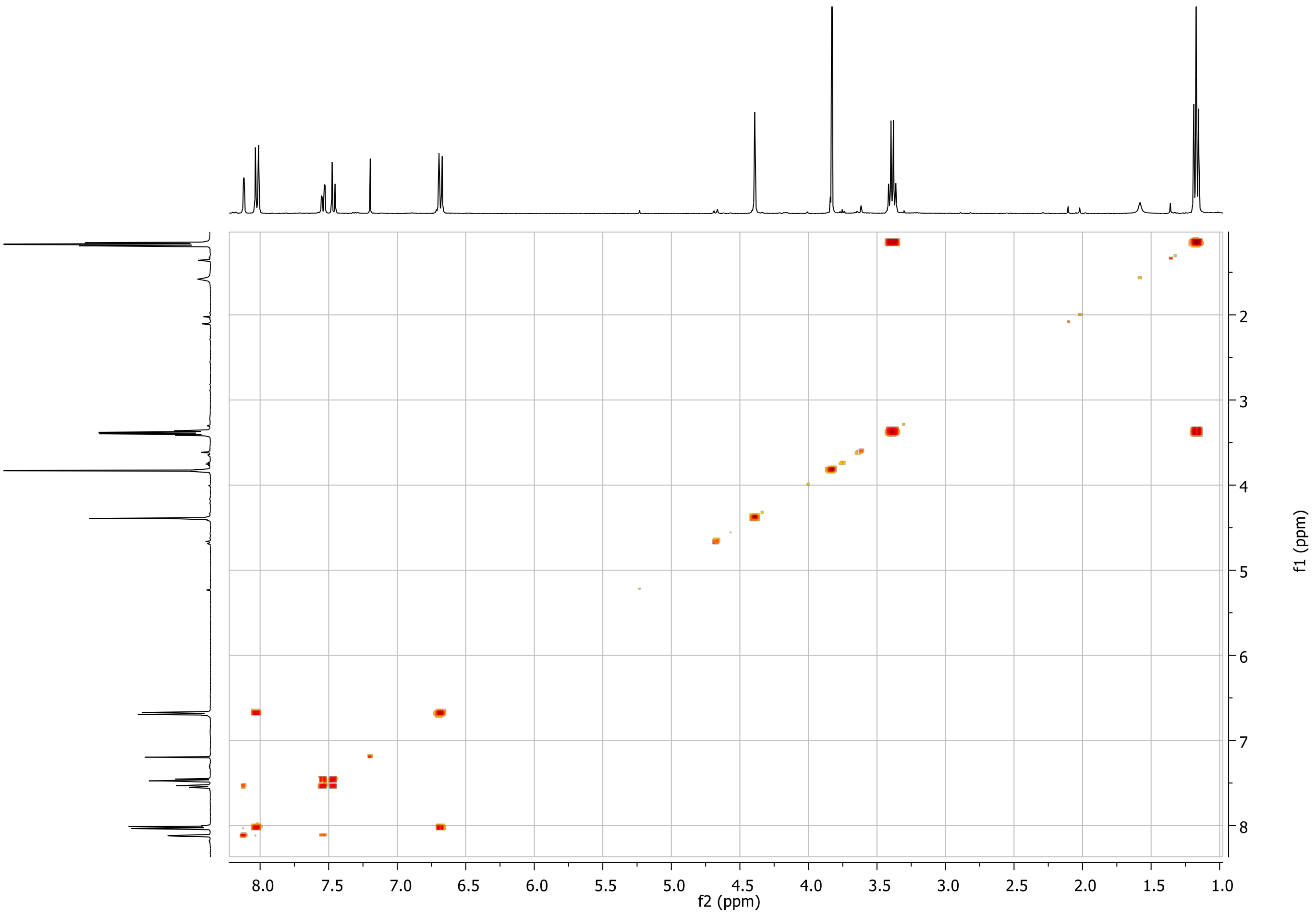
8.0
7.5
7.0
6.5
6.0
5.5
5.0
4.5
4.0
3.5
3.0
2.5
2.0
1.5
1.0
f2 (ppm)
2
3
4
5
6
7
8
f1 (ppm)

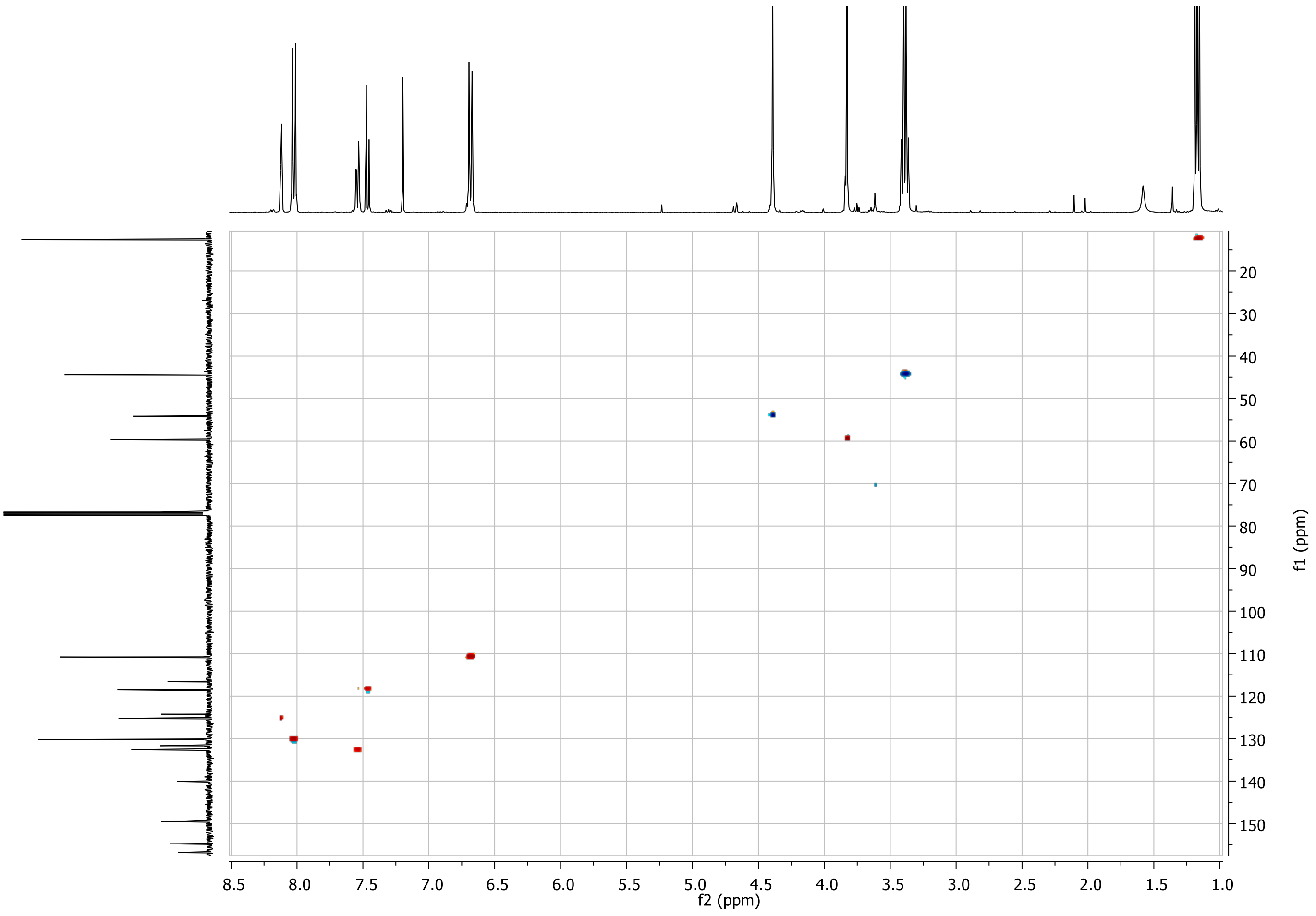
8.5
8.0
7.5
7.0
6.5
6.0
5.5
5.0
4.5
4.0
3.5
3.0
2.5
2.0
1.5
1.0
f2 (ppm)
20
30
40
50
60
70
80
90
100
110
120
130
140
150
f1 (ppm)

Figure S29. ^{1}H-, ^{13}C-, & COSY-NMR spectra of 7: 6-(bromomethyl)-2-(4-(diethylamino)phenyl)-3-methoxy-4H-chromen-4-one.

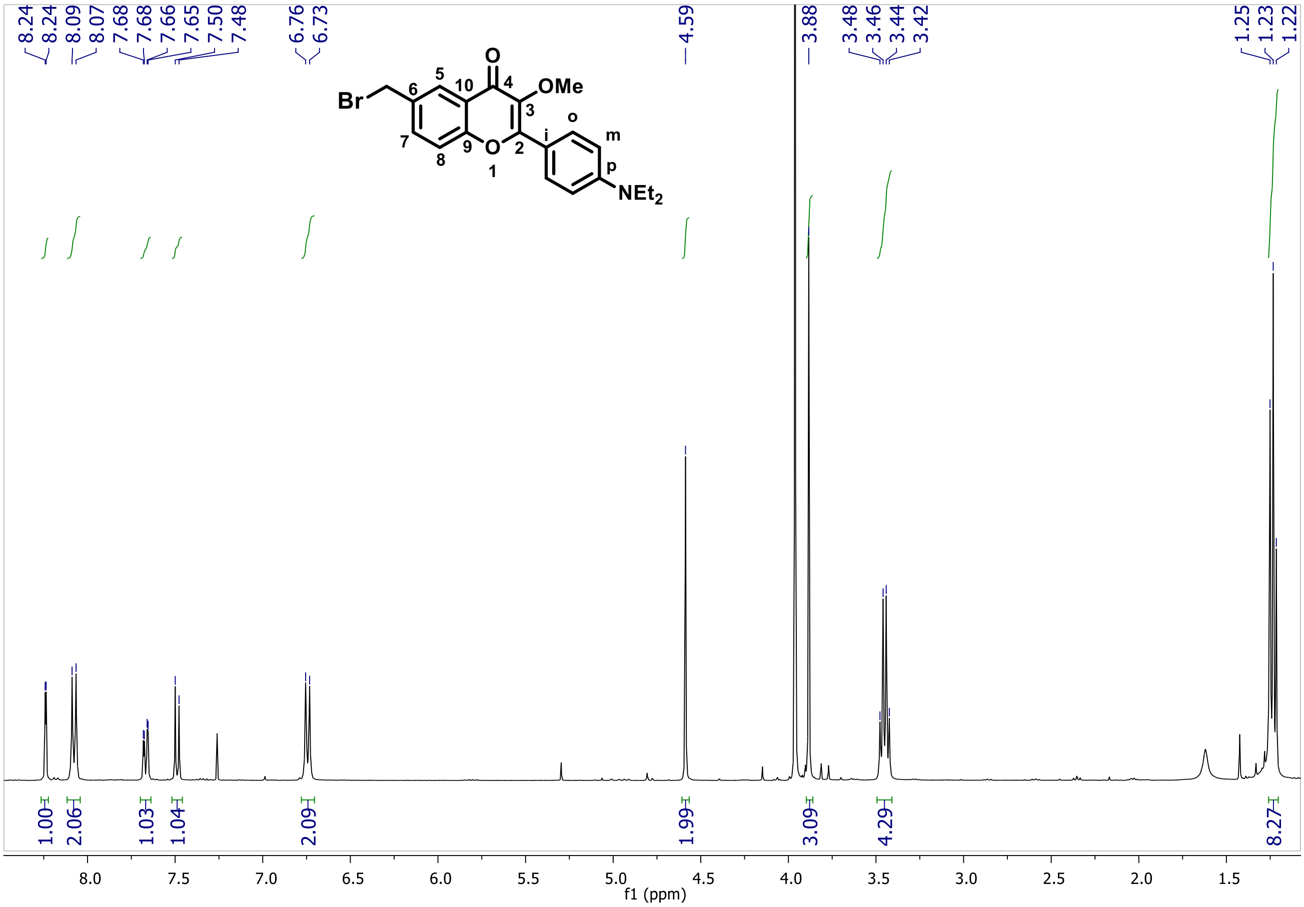

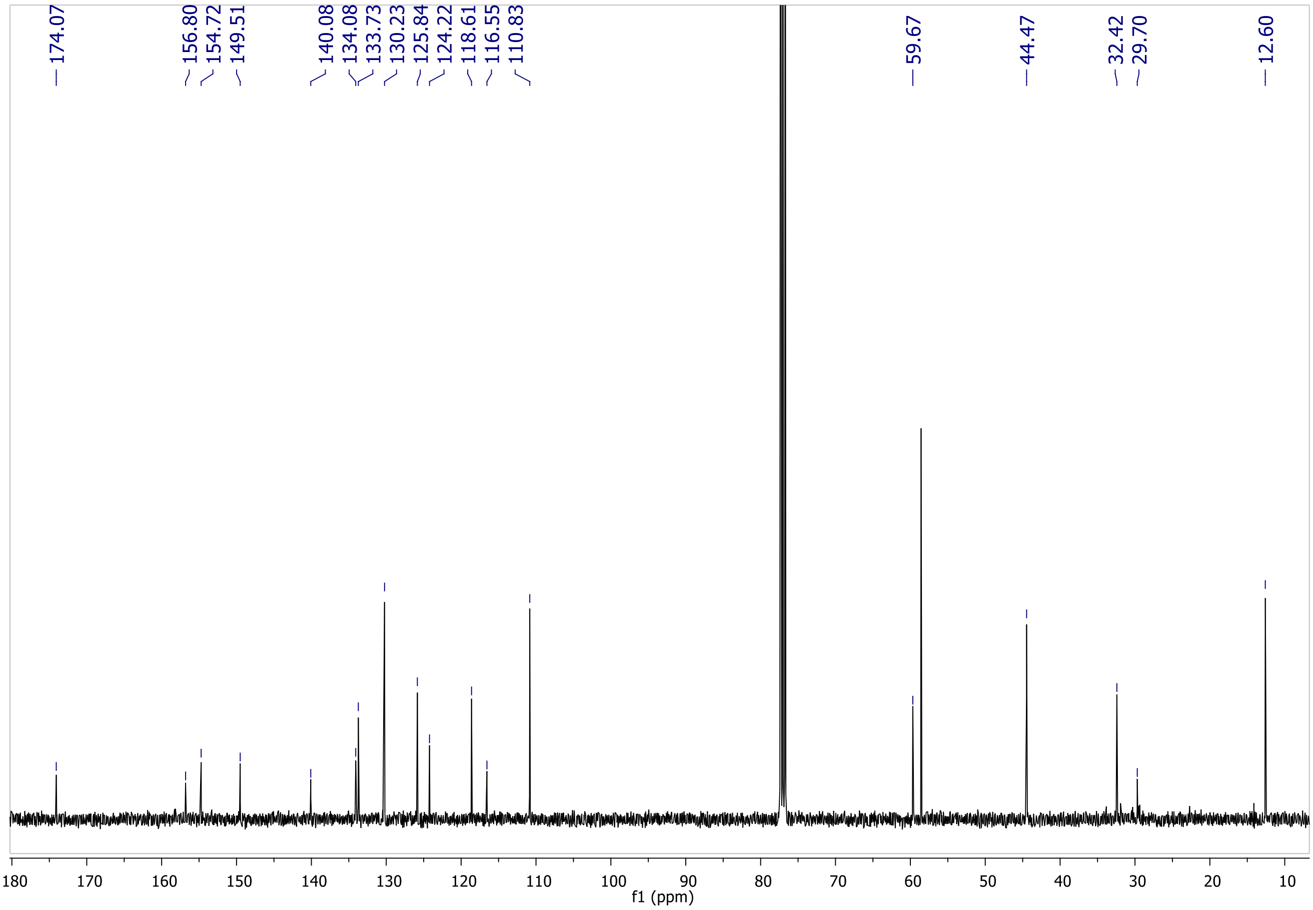

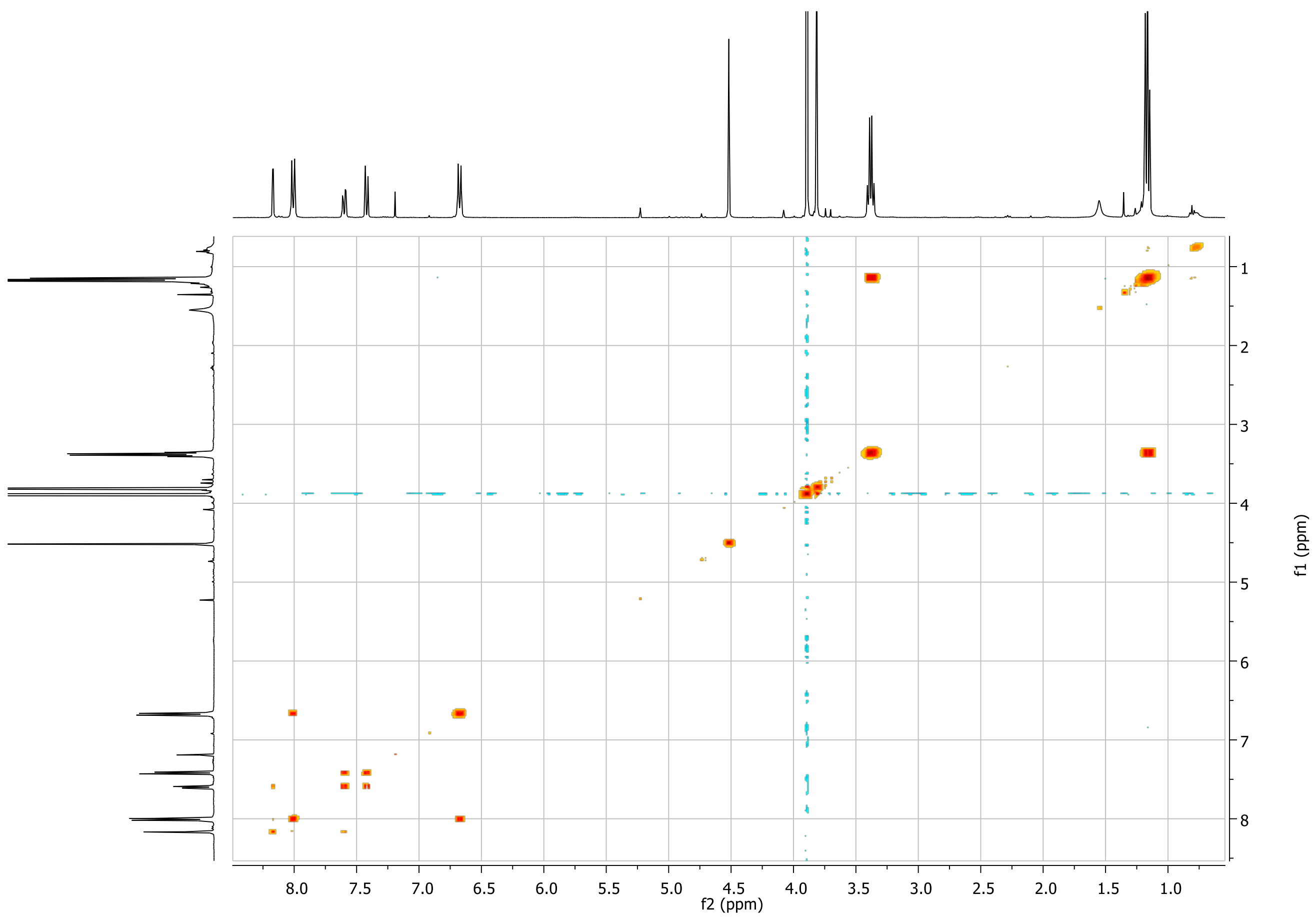

Figure S30. ^{1}H- & ^{13}C-NMR spectra of PYAPS: Acid 3-(prop-2-yn-1-ylamino)propane-1-sulfonic.

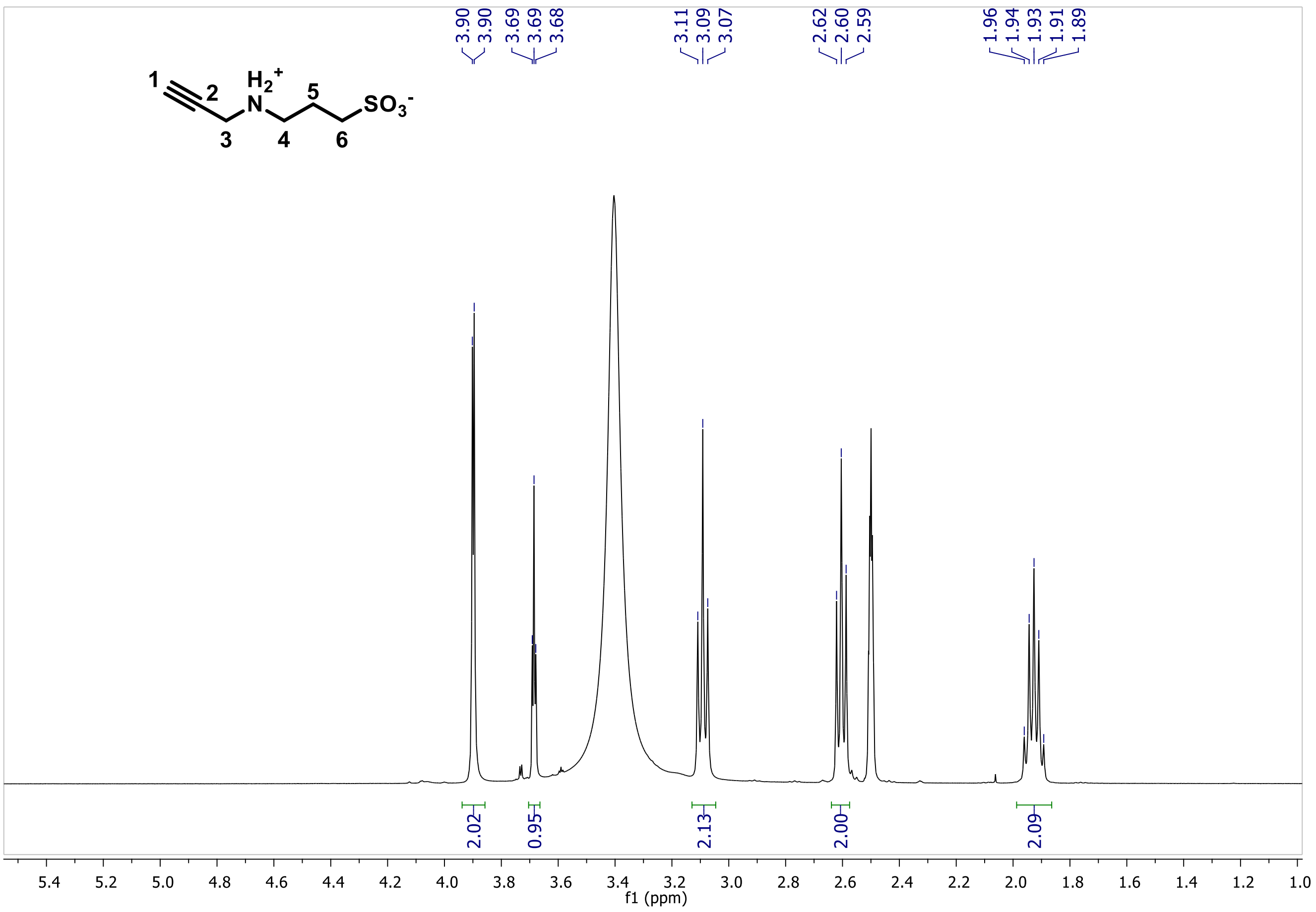

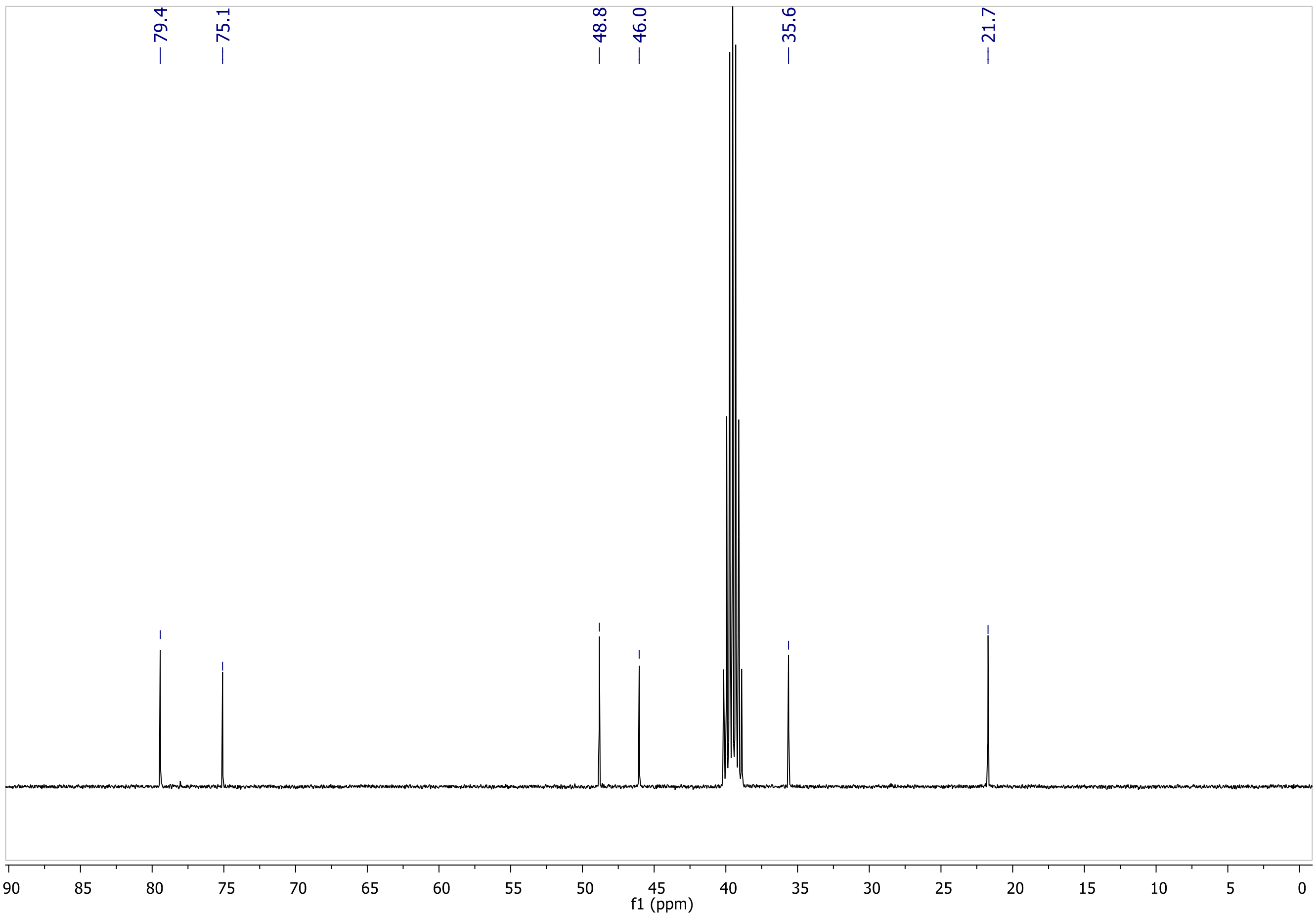

***Figure S31.** ^{1}H- & ^{13}C-NMR spectra of Me-PYAPS: 3-(methyl(prop-2-yn-1-yl)amino)propane-1-sulfonate.*

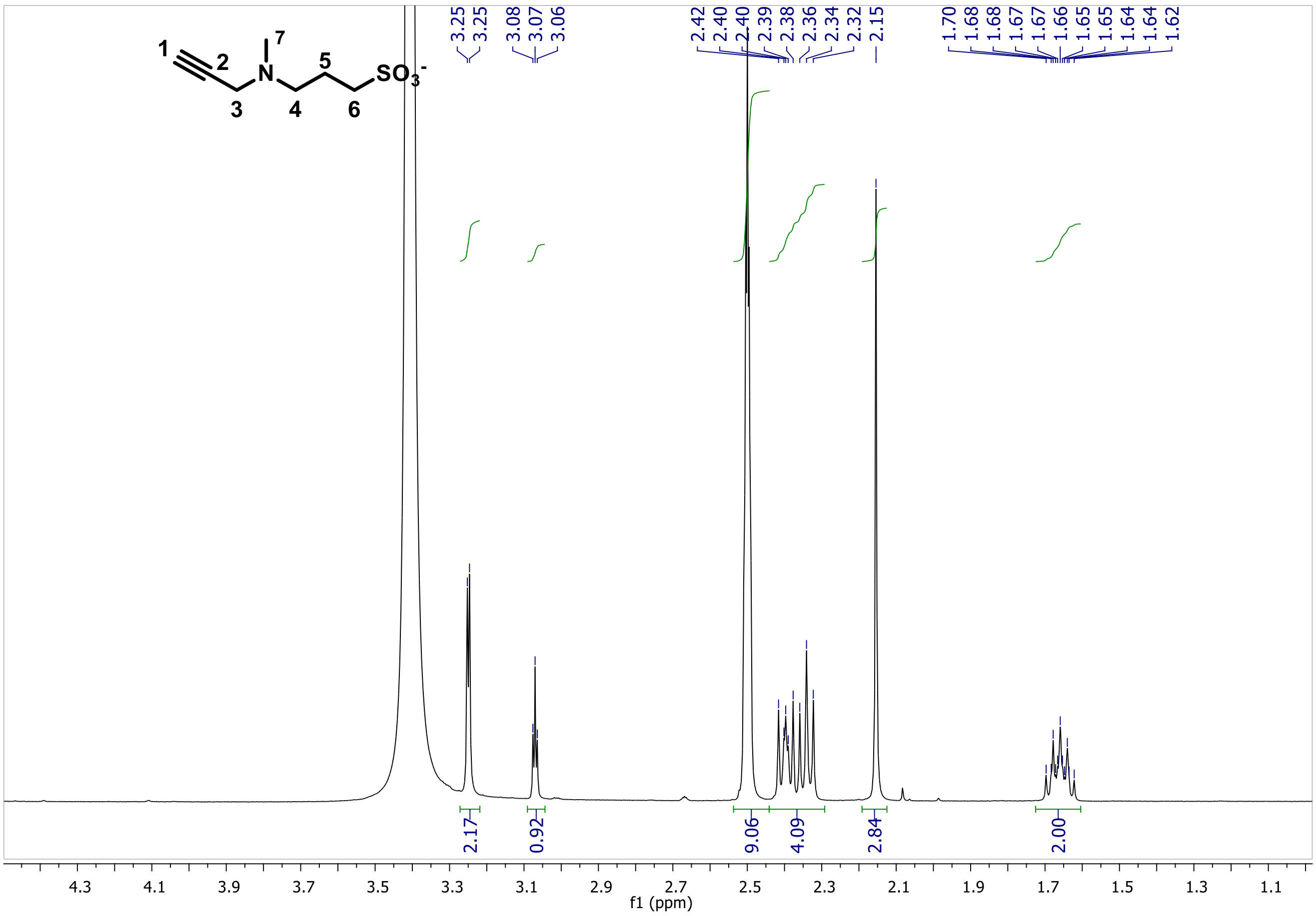

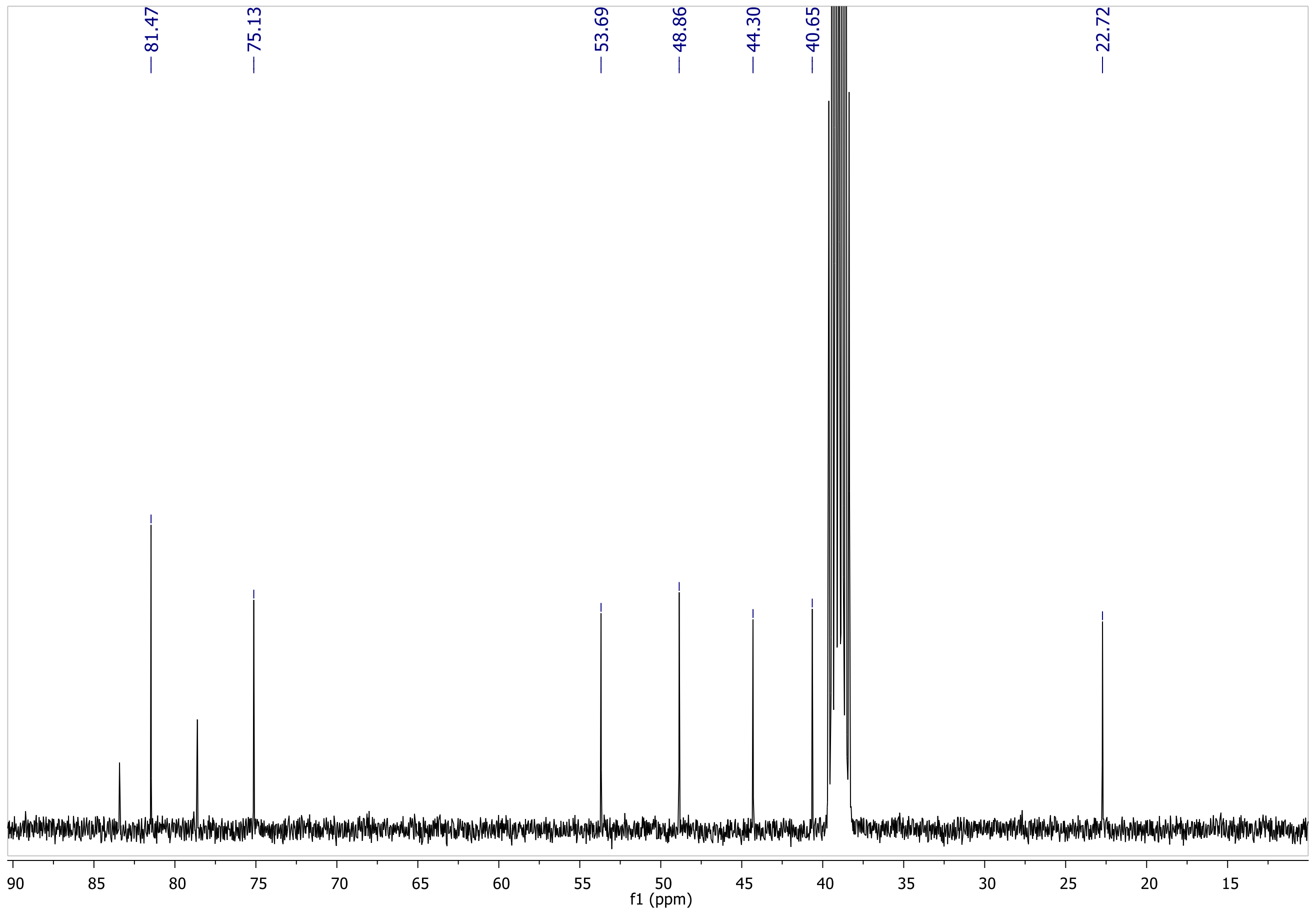

Figure S32. ^{1}H- & ^{13}C-NMR spectra of DMAZ: 2-azido-N,N-dimethylethanamine.

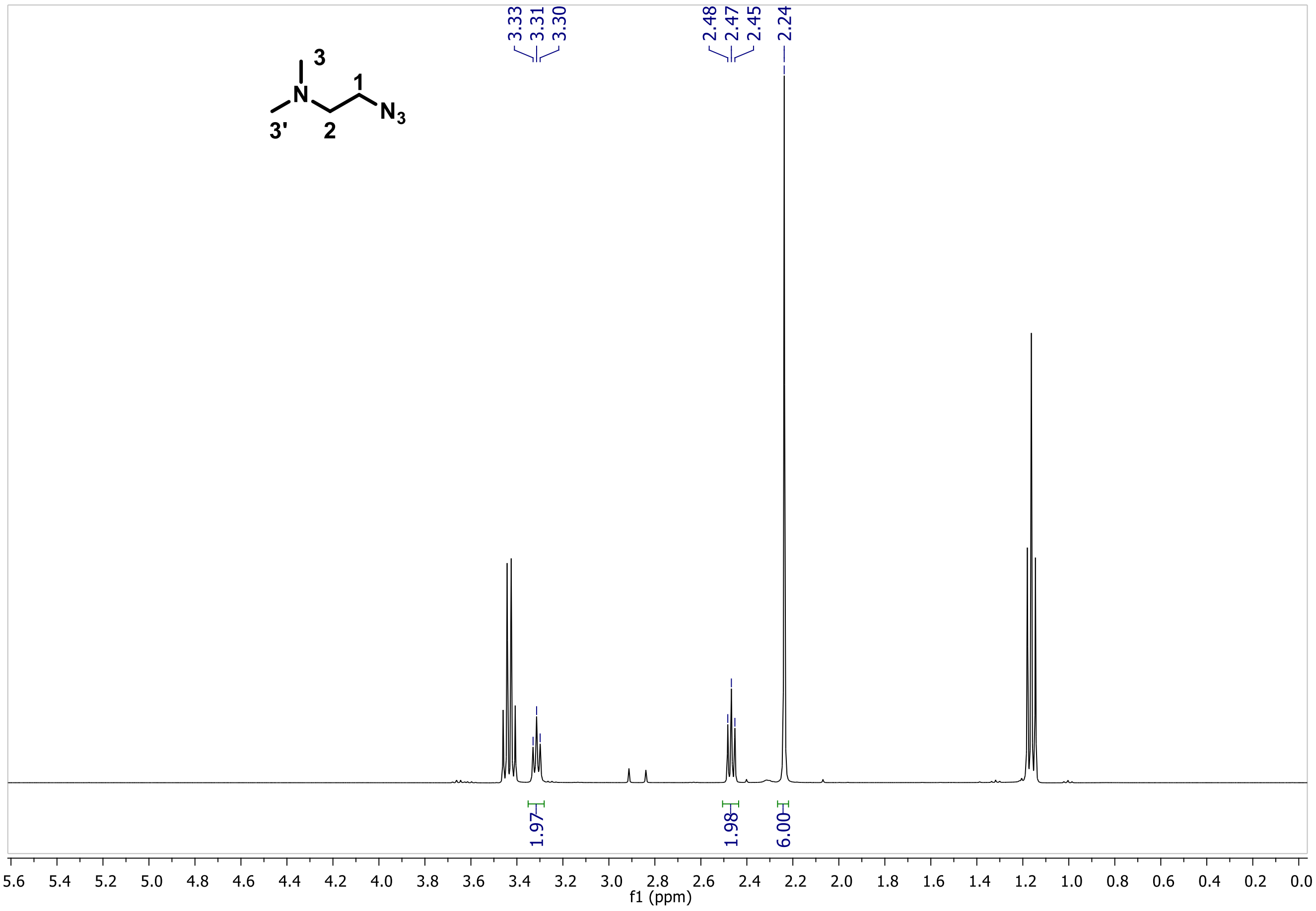

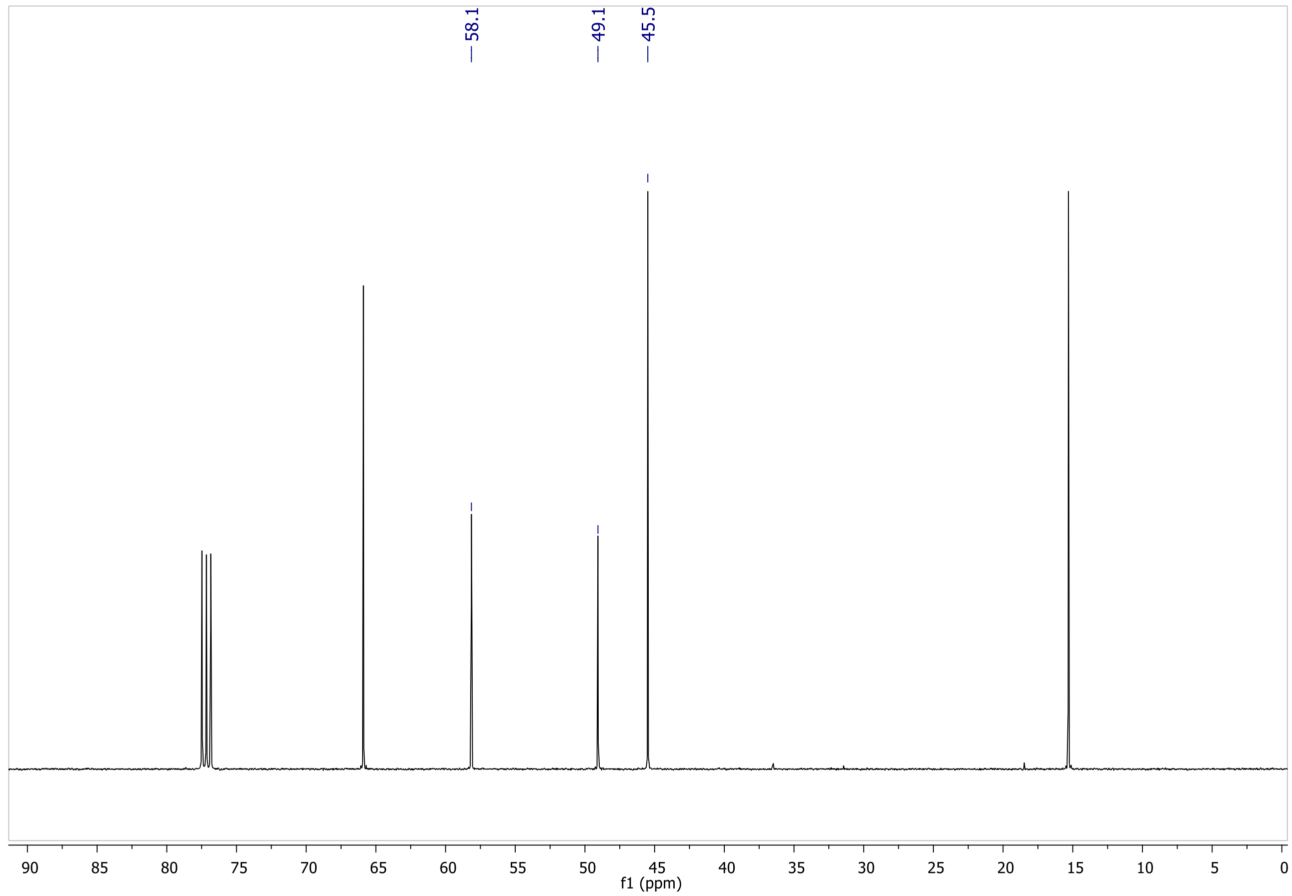

Figure S33.** [1]**H-NMR spectrum of AlMF−: 3-(((2-(4-(diethylamino)phenyl)-3-methoxy-4-oxo-4H-chromen-6-yl)methyl)(prop-2-yn-1-yl)amino)propane-1-sulfonate.

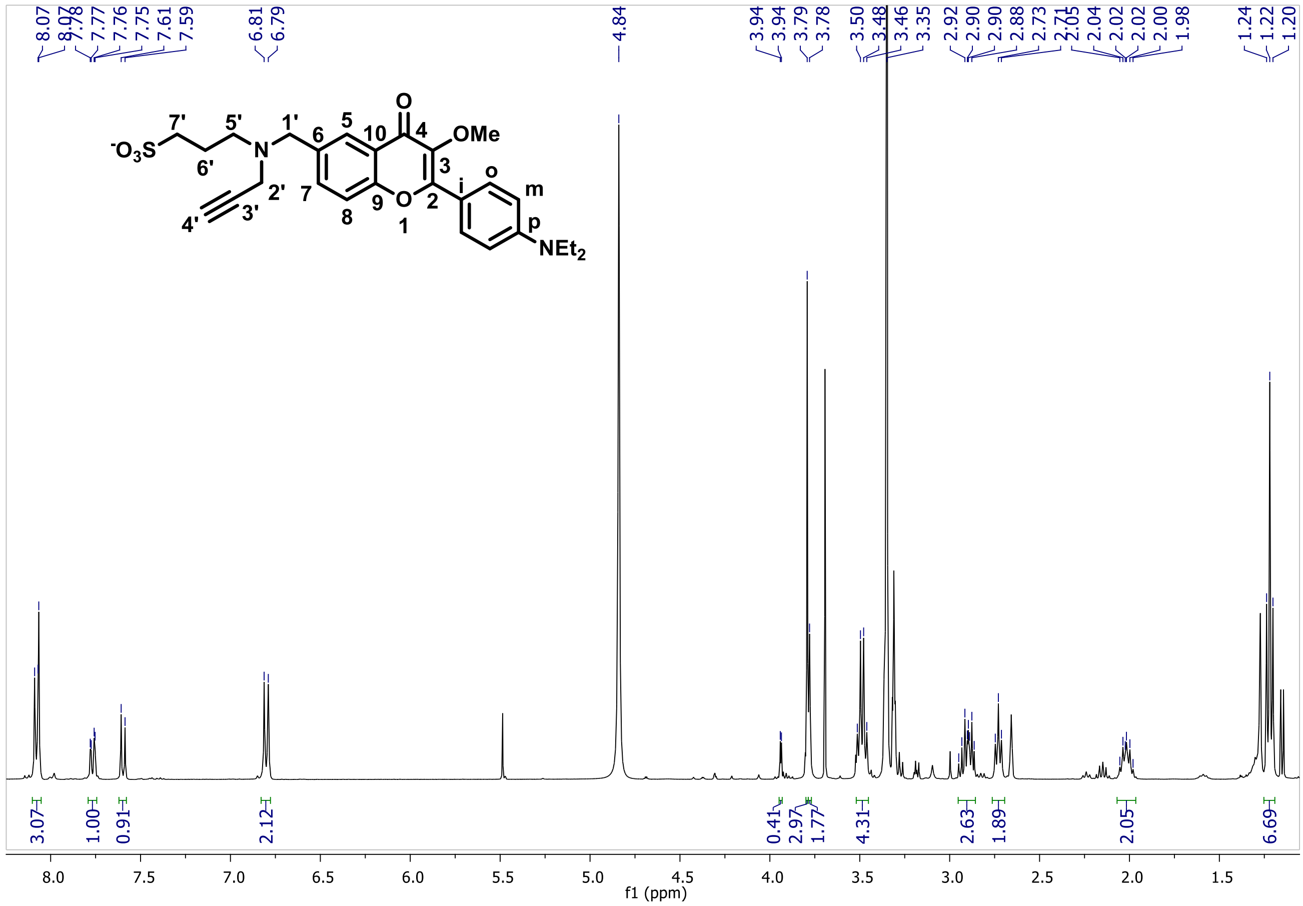

Figure S34. ^{1}H-, ^{13}C-, & COSY-NMR spectra of AlMF+−: 3-(((2-(4-(diethylamino)phenyl)-3-methoxy-4-oxo-4H-chromen-6-yl)methyl)(methyl)(prop-2-yn-1-yl)ammonio)propane-1-sulfonate.

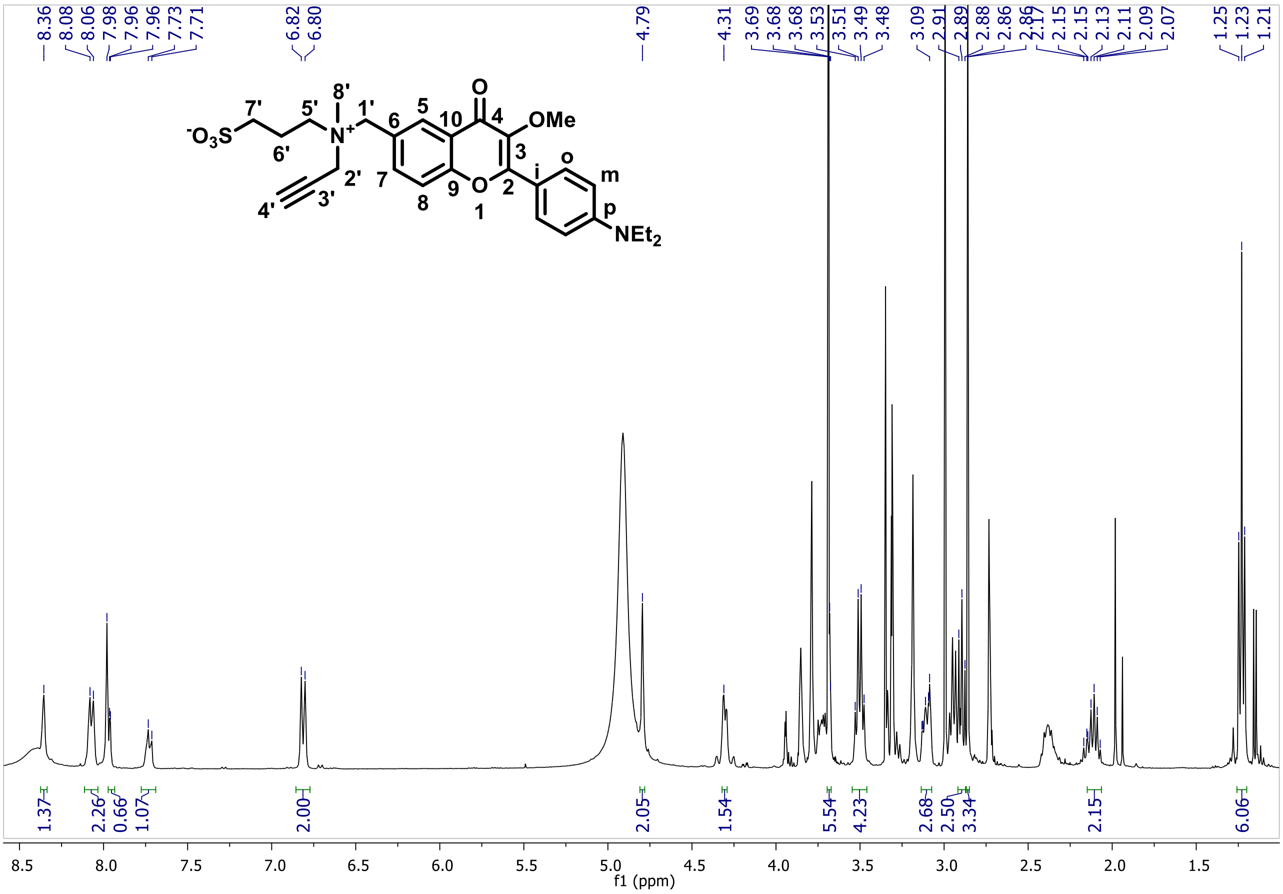

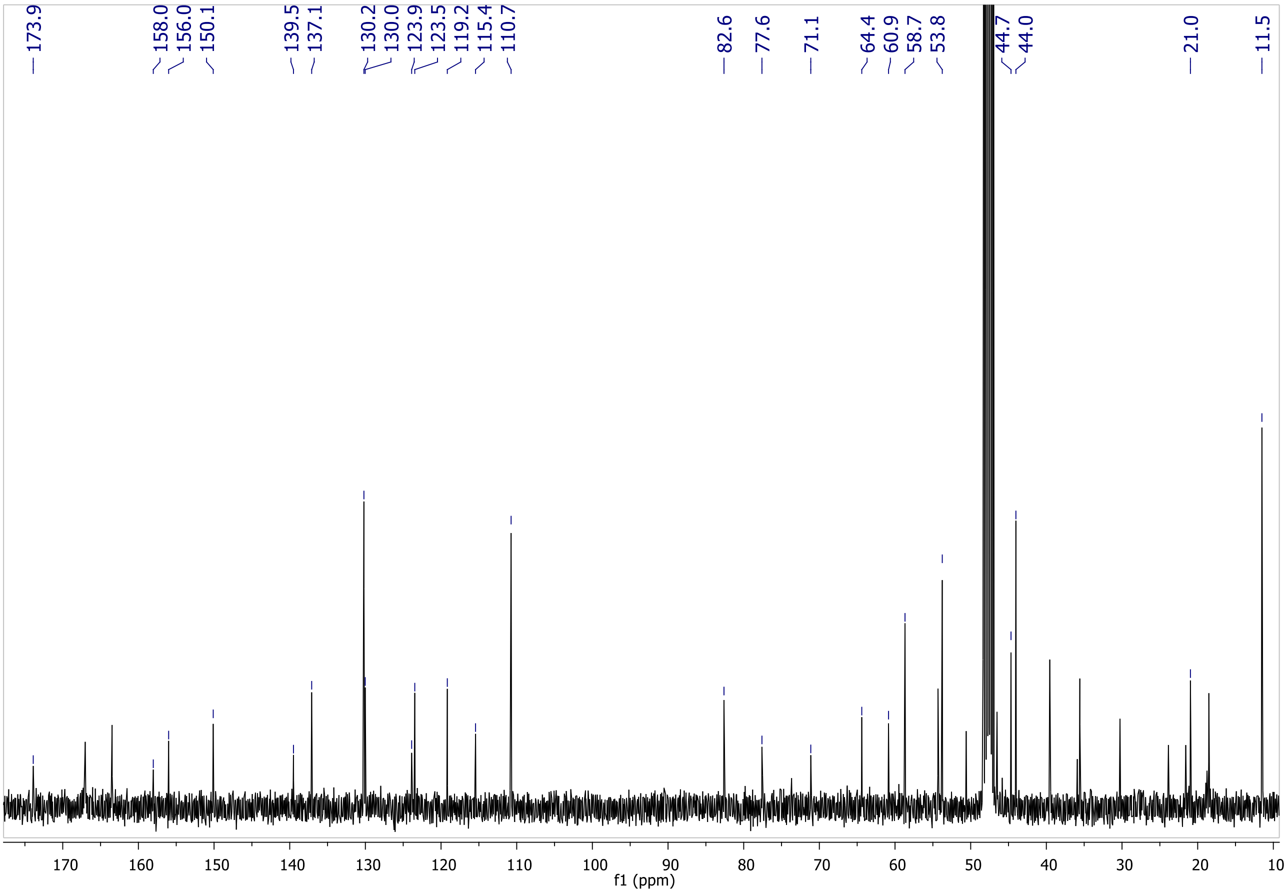

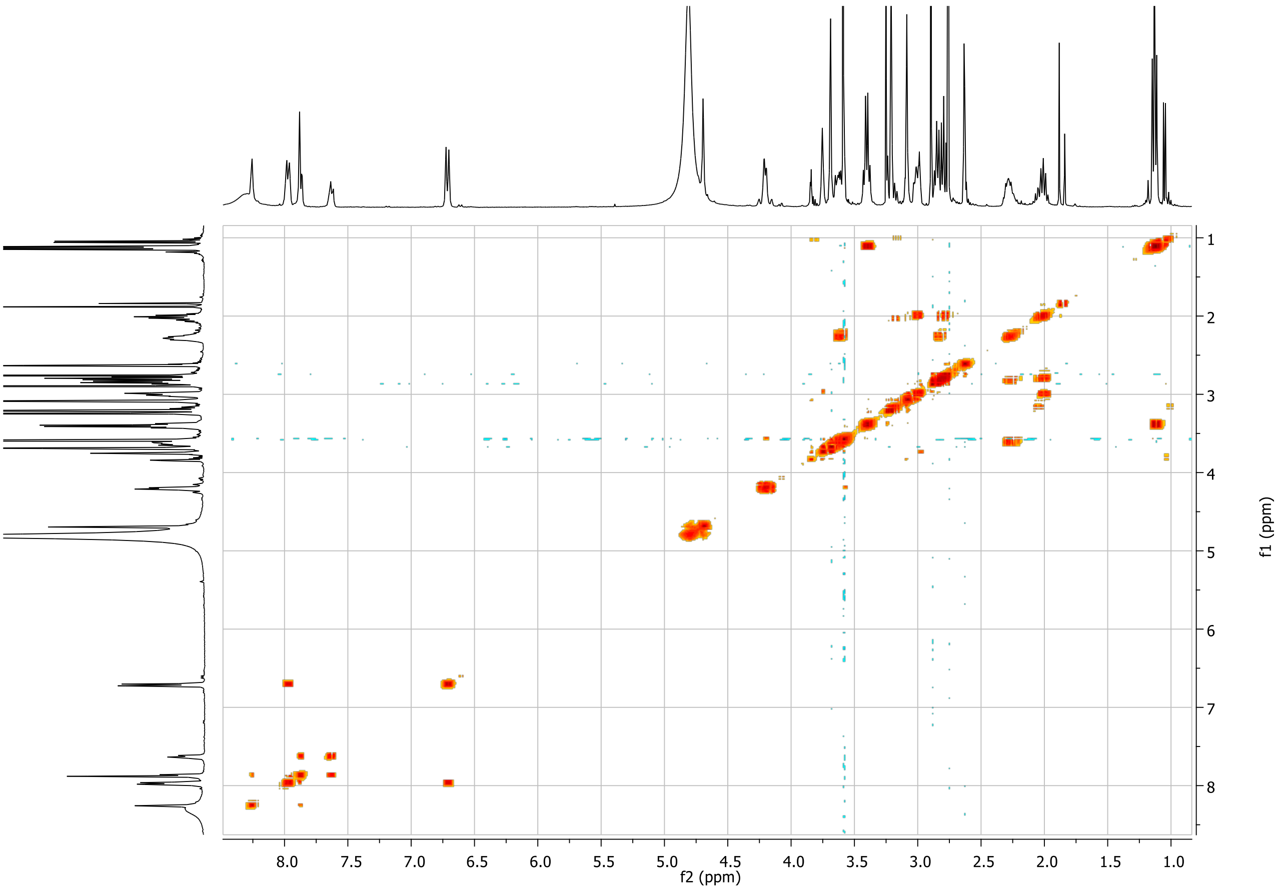

***Figure S35.** ^{1}H-, ^{13}C-, & COSY-NMR spectra of AzMF+: 2-azido-N-((2-(4-(diethylamino)phenyl)-3-methoxy-4-oxo-4H-chromen-6-yl)methyl)-N,N-dimethylethanaminium bromide.*

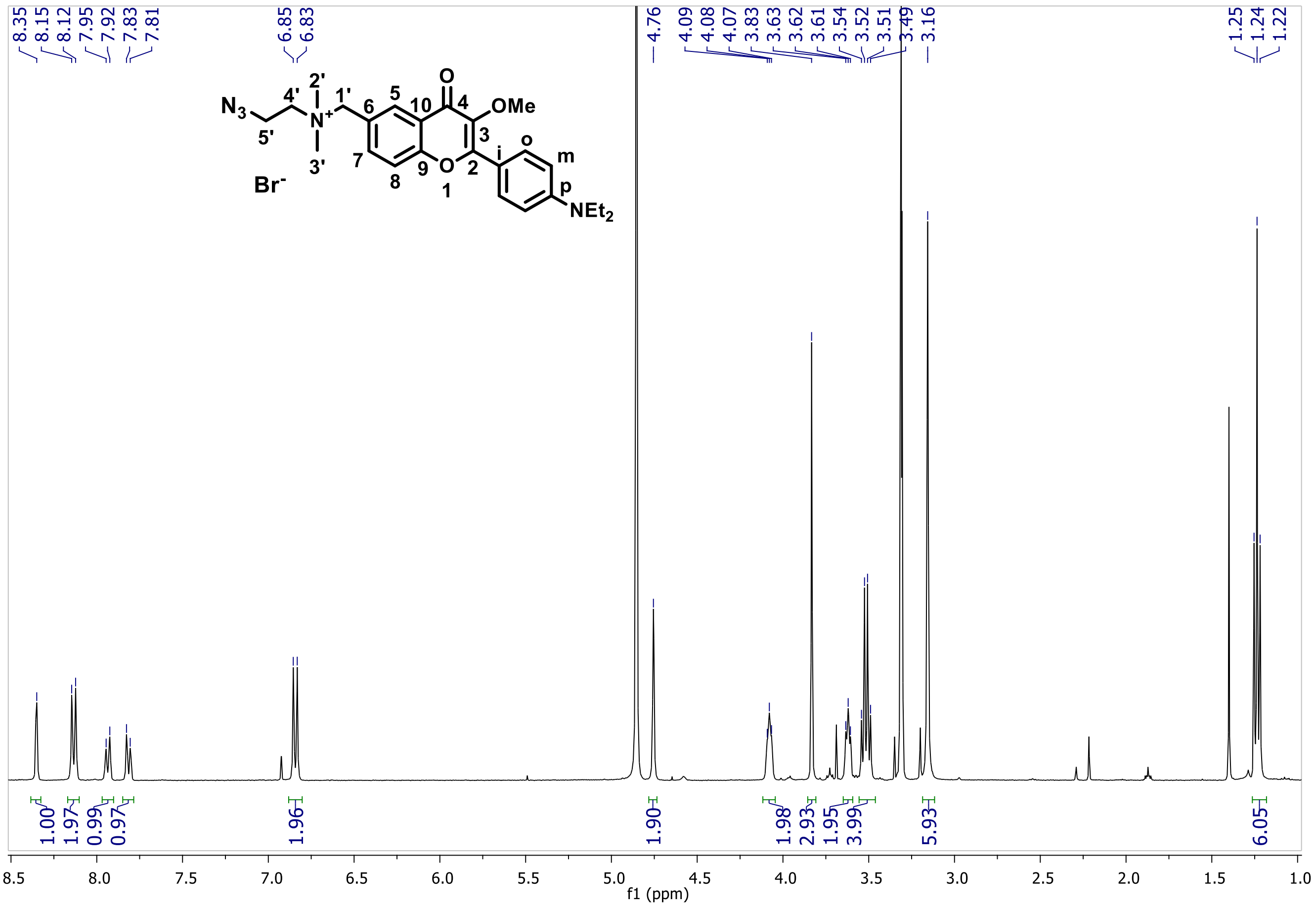

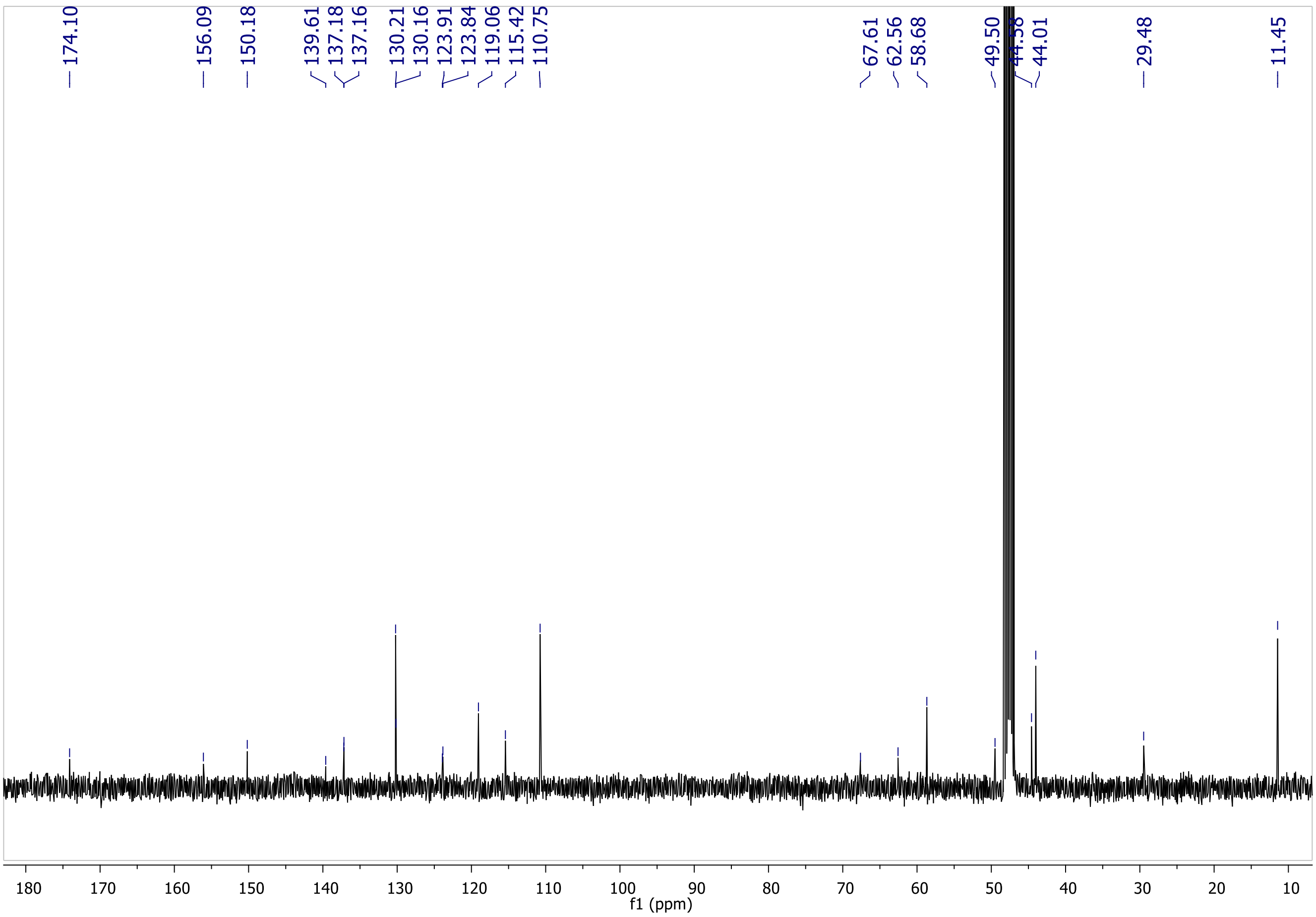
174.10
156.09
150.18
139.61
137.18
137.16
130.21
130.16
123.91
123.84
119.06
115.42
110.75
67.61
62.56
58.68
49.50
44.58
44.01
29.48
11.45
f1 (ppm)

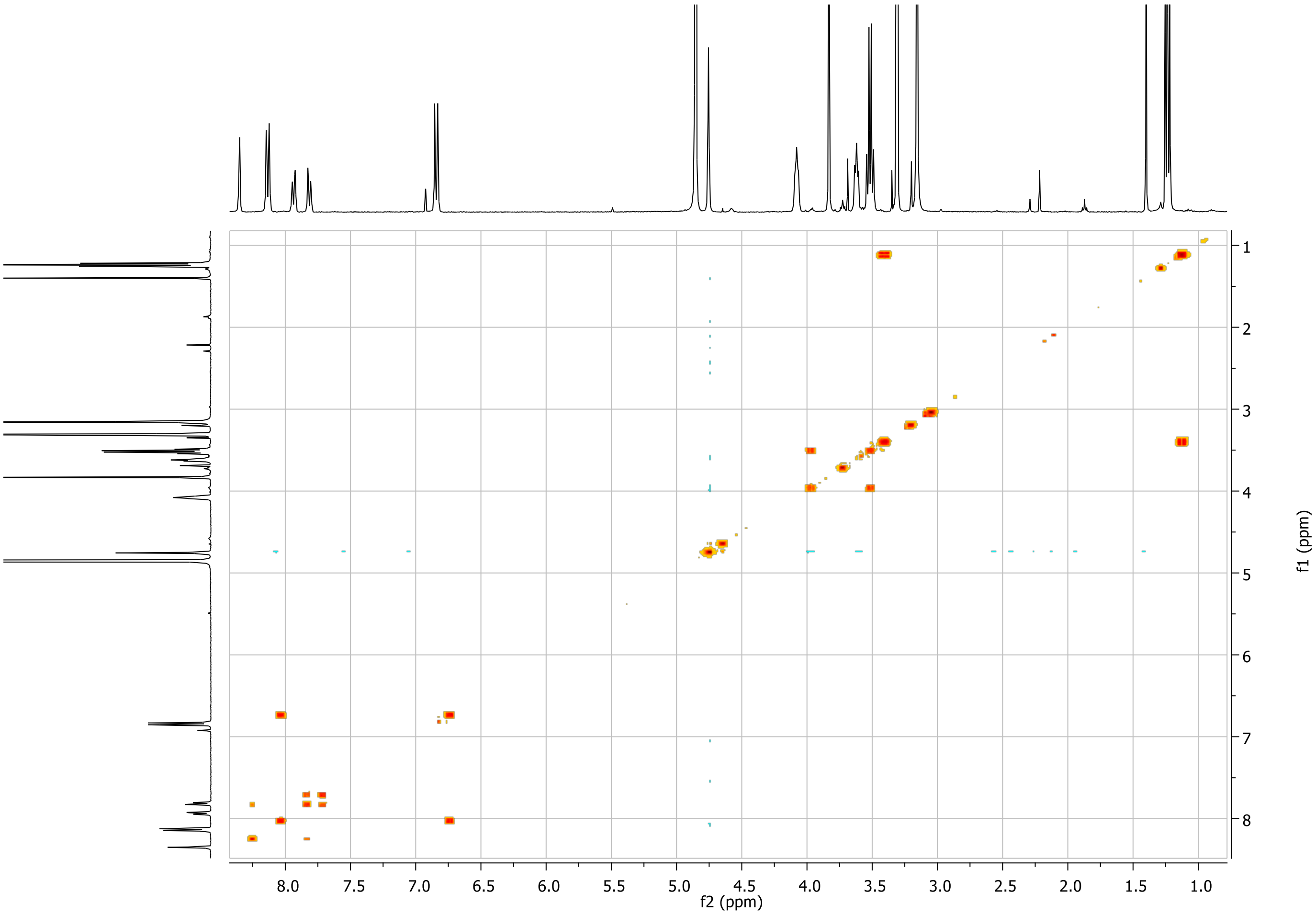
f2 (ppm)
f1 (ppm)

Figure S36. ***^{1}H- & ^{13}C-NMR spectra of 8: (2R,3S,5R)-5-azidotetrahydrofuran-2,3-diyl bis-(4-methylbenzoate).***

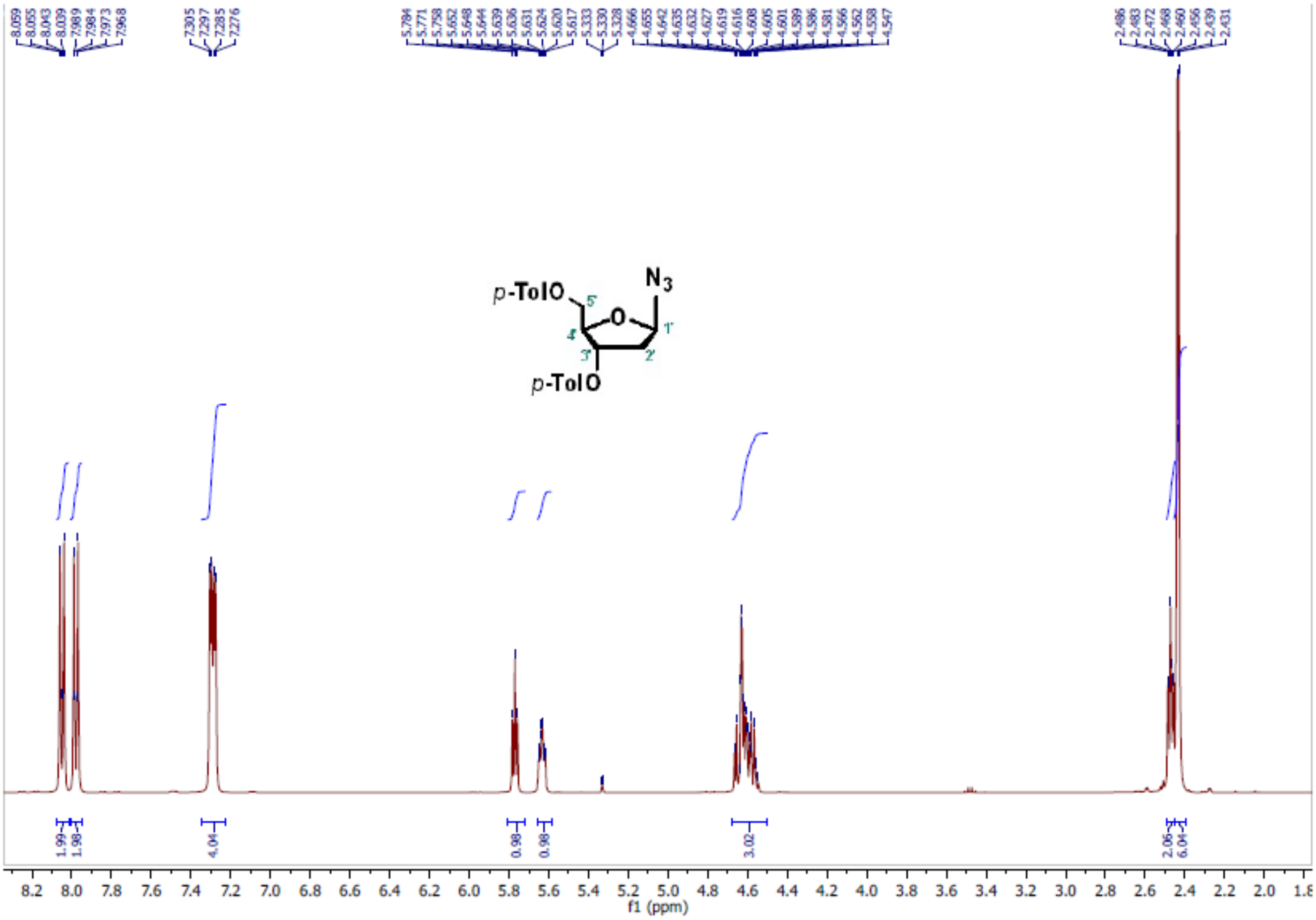

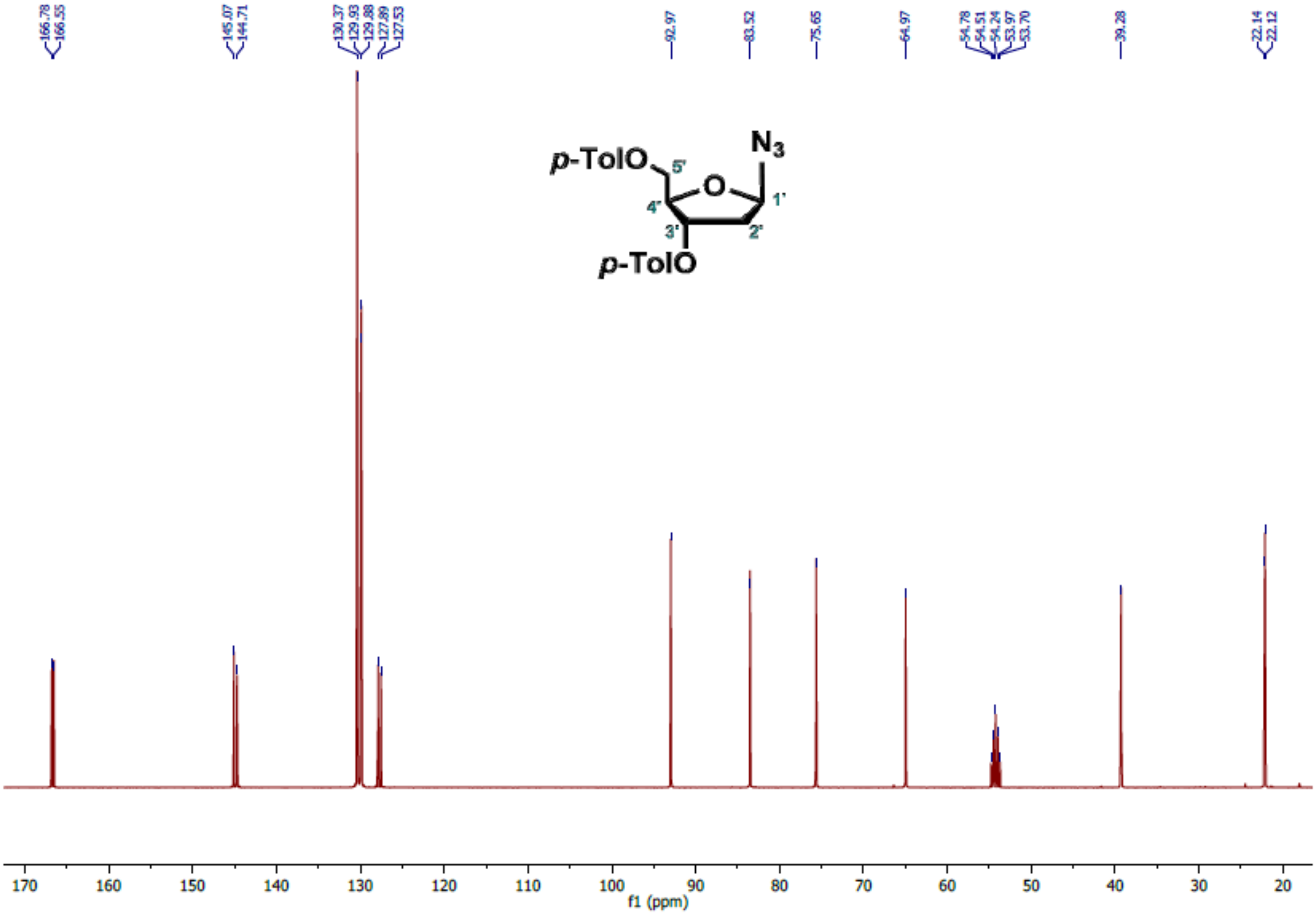

Figure S37. ^{1}H-, ^{13}C-, COSY-, HSQC-, HMBC-NMR spectra of 9: (2R,3S,5S)-5-(4-(2-(4-(diethylamino)phenyl)-3-methoxy-4-oxo-4H-chromen-6-yl)-1H-1,2,3-triazol-1-yl)-2-(((4-methylbenzoyl)oxy)methyl)tetrahydrofuran-3-yl 4-methylbenzoate.

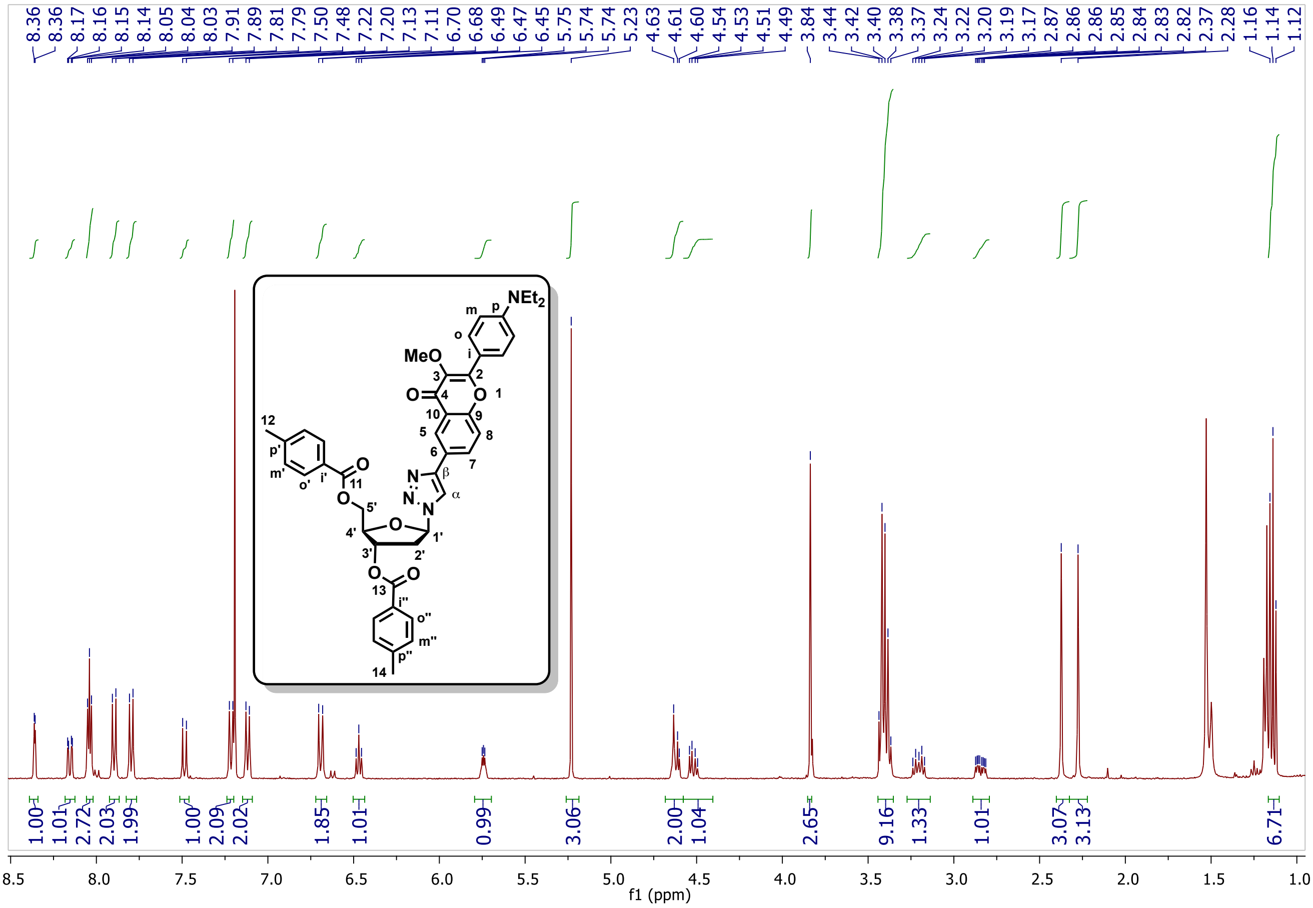

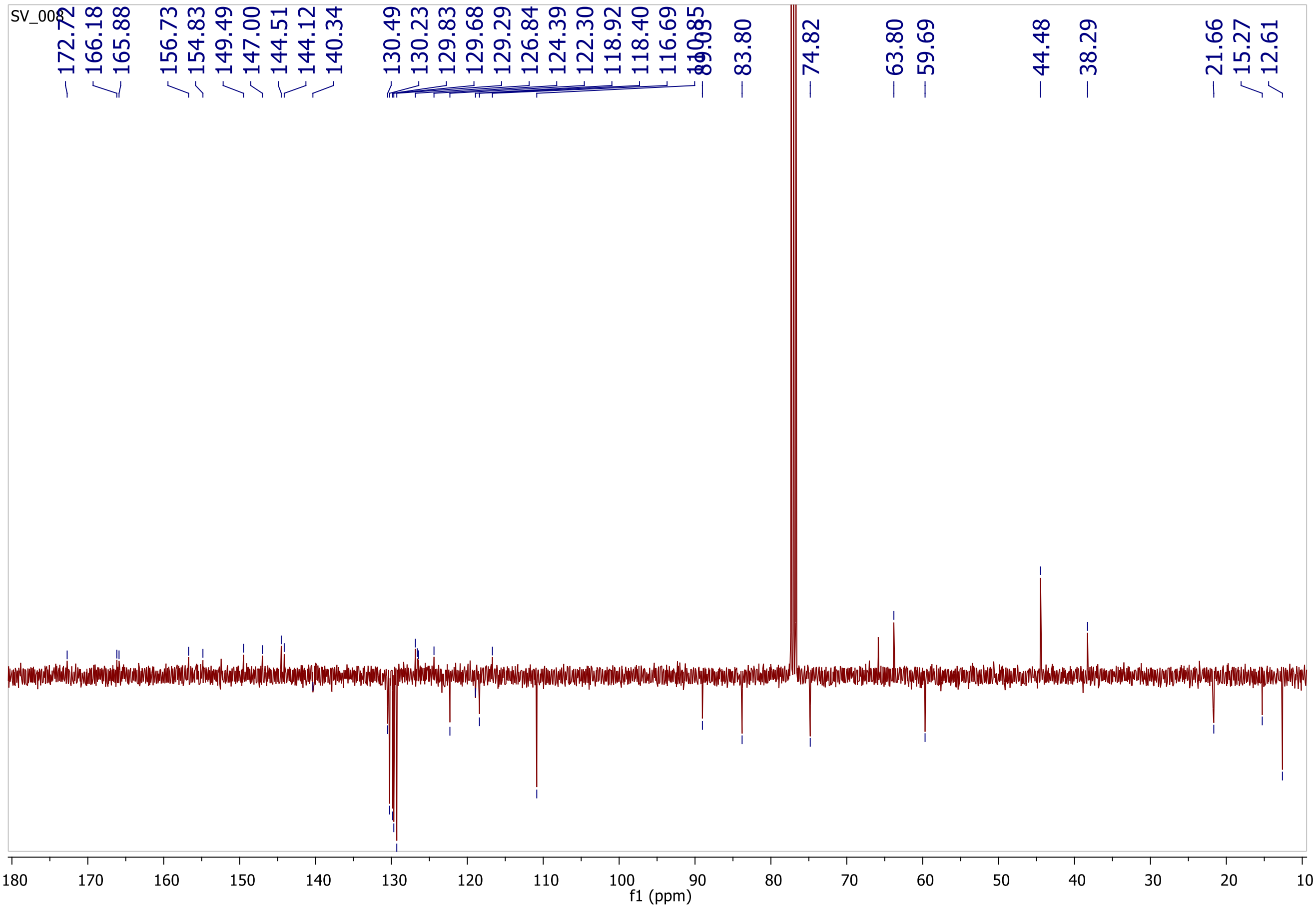

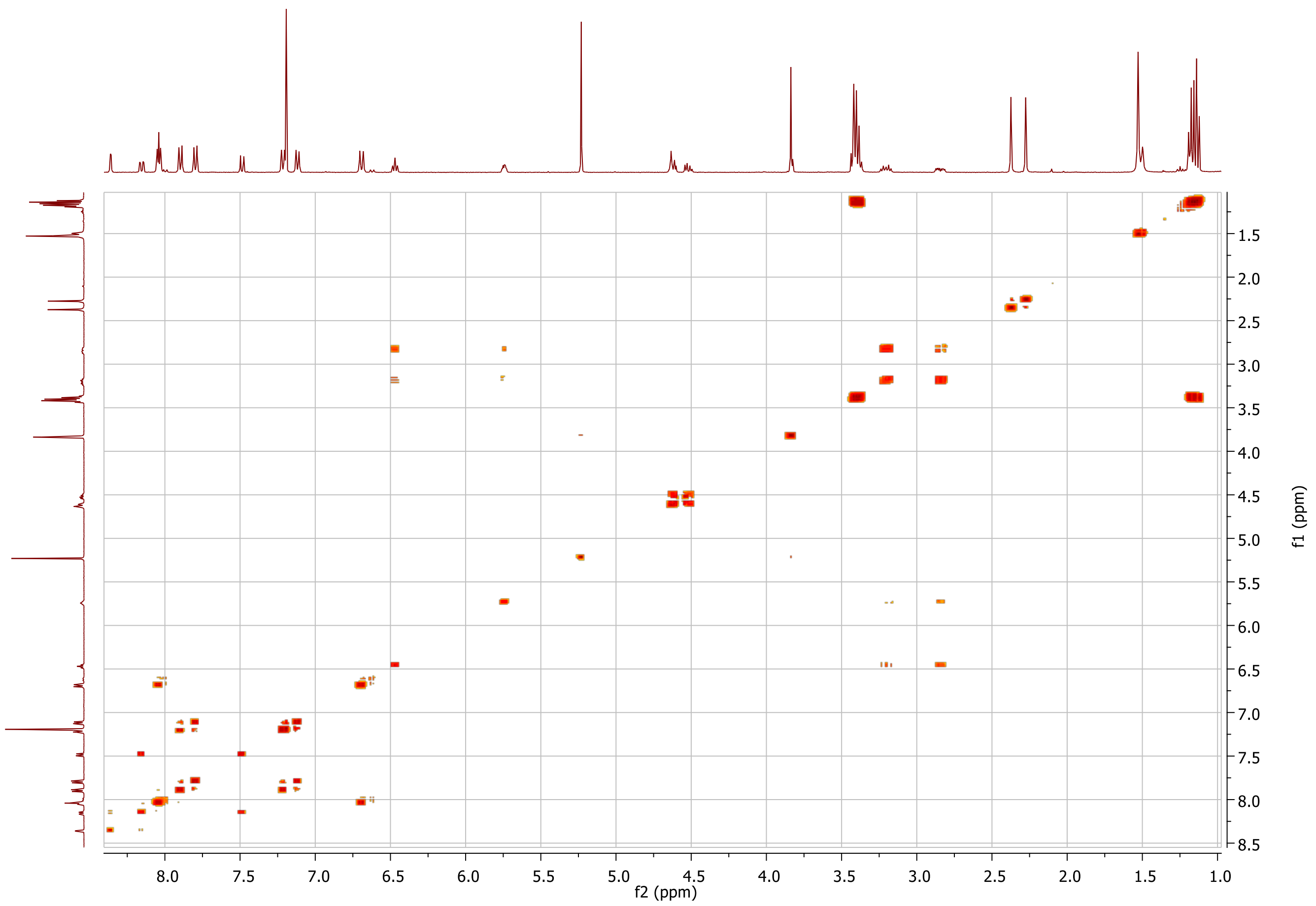
f2 (ppm)
f1 (ppm)

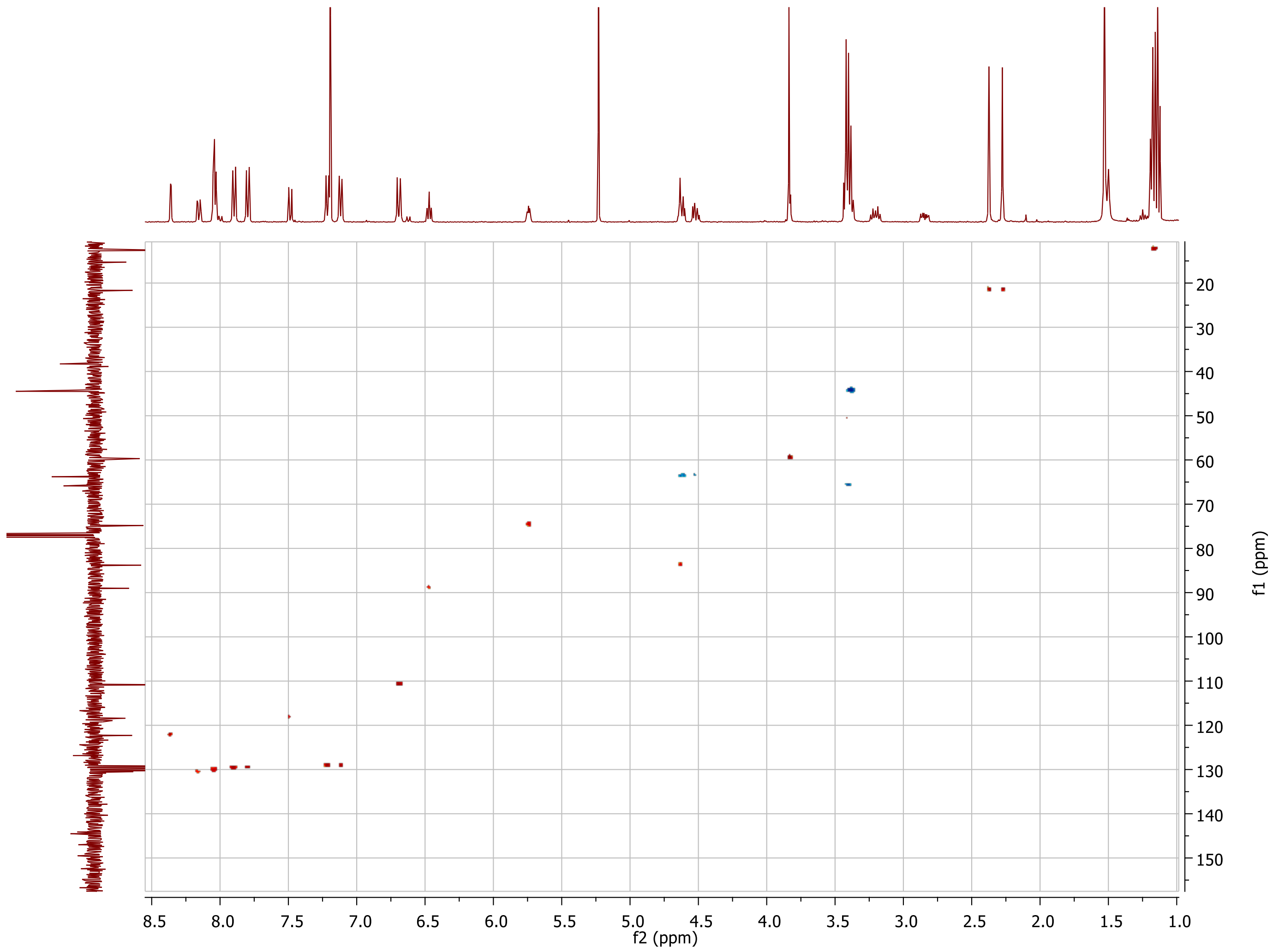
f2 (ppm)
f1 (ppm)

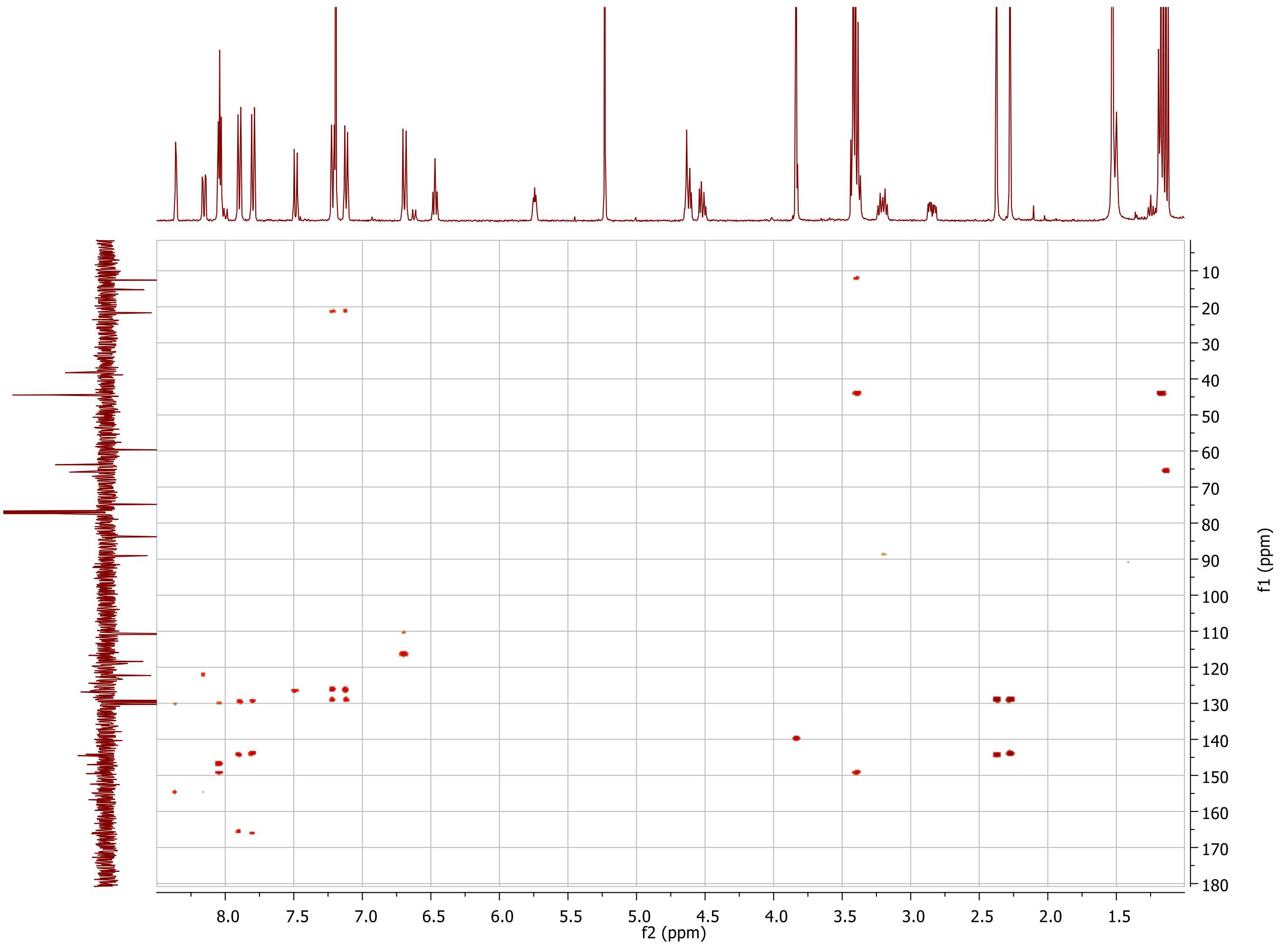

***Figure S38.** ^{1}H-, ^{13}C-, COSY-, HSQC-, HMBC-NMR spectra of AlMF-Nu: 2-(4-(diethylamino)phenyl)-6-(1-((2S,4S,5R)-4-hydroxy-5-(hydroxymethyl)tetrahydrofuran-2-yl)-1H-1,2,3-triazol-4-yl)-3-methoxy-4H-chromen-4-one.*

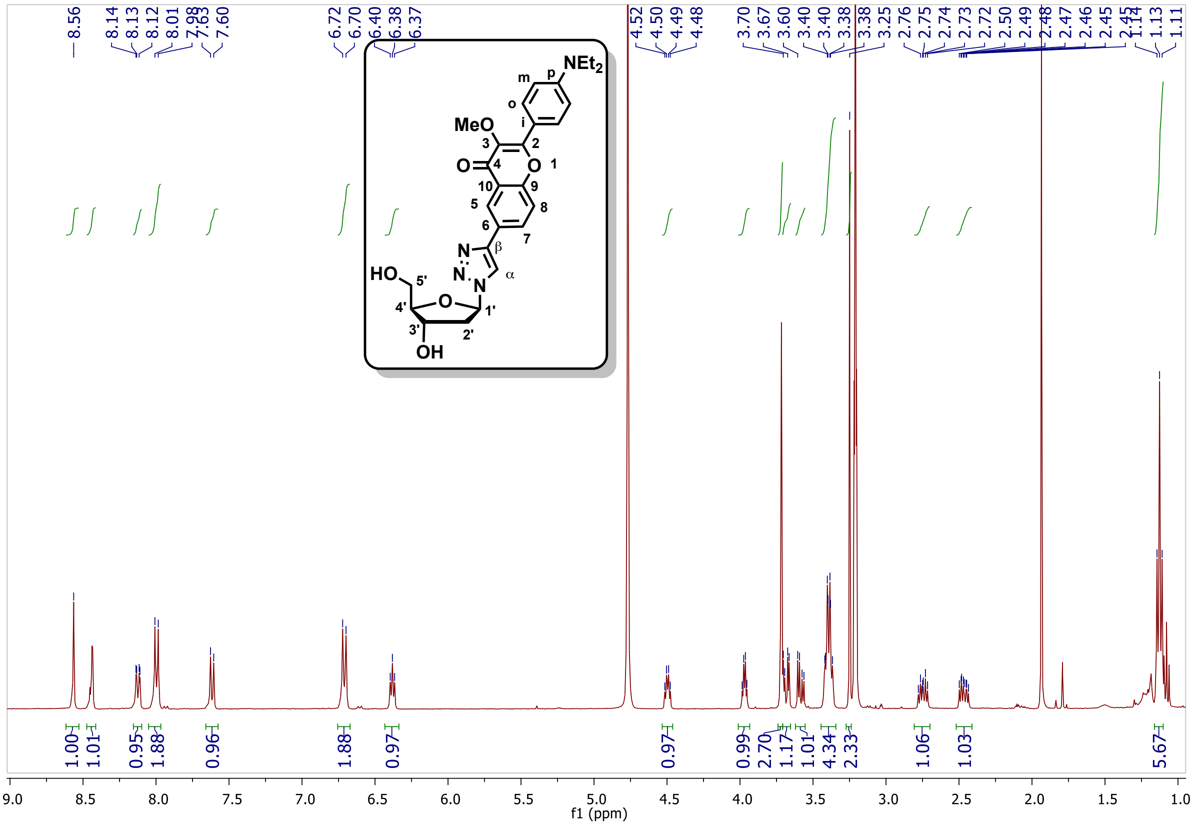

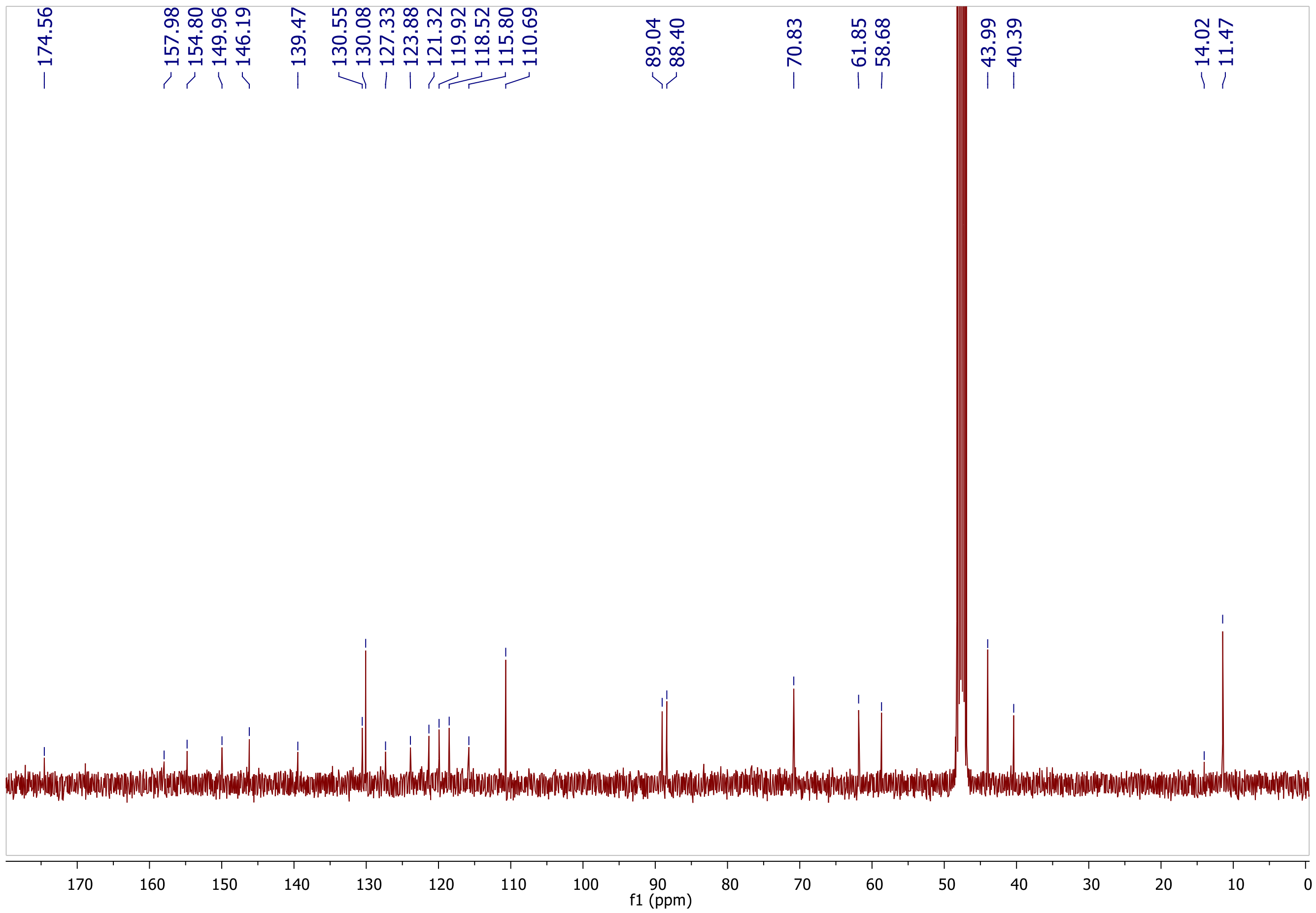
174.56
157.98
154.80
149.96
146.19
139.47
130.55
130.08
127.33
123.88
121.32
119.92
118.52
115.80
110.69
89.04
88.40
70.83
61.85
58.68
43.99
40.39
14.02
11.47
170 160 150 140 130 120 110 100 90 80 70 60 50 40 30 20 10 0
f1 (ppm)

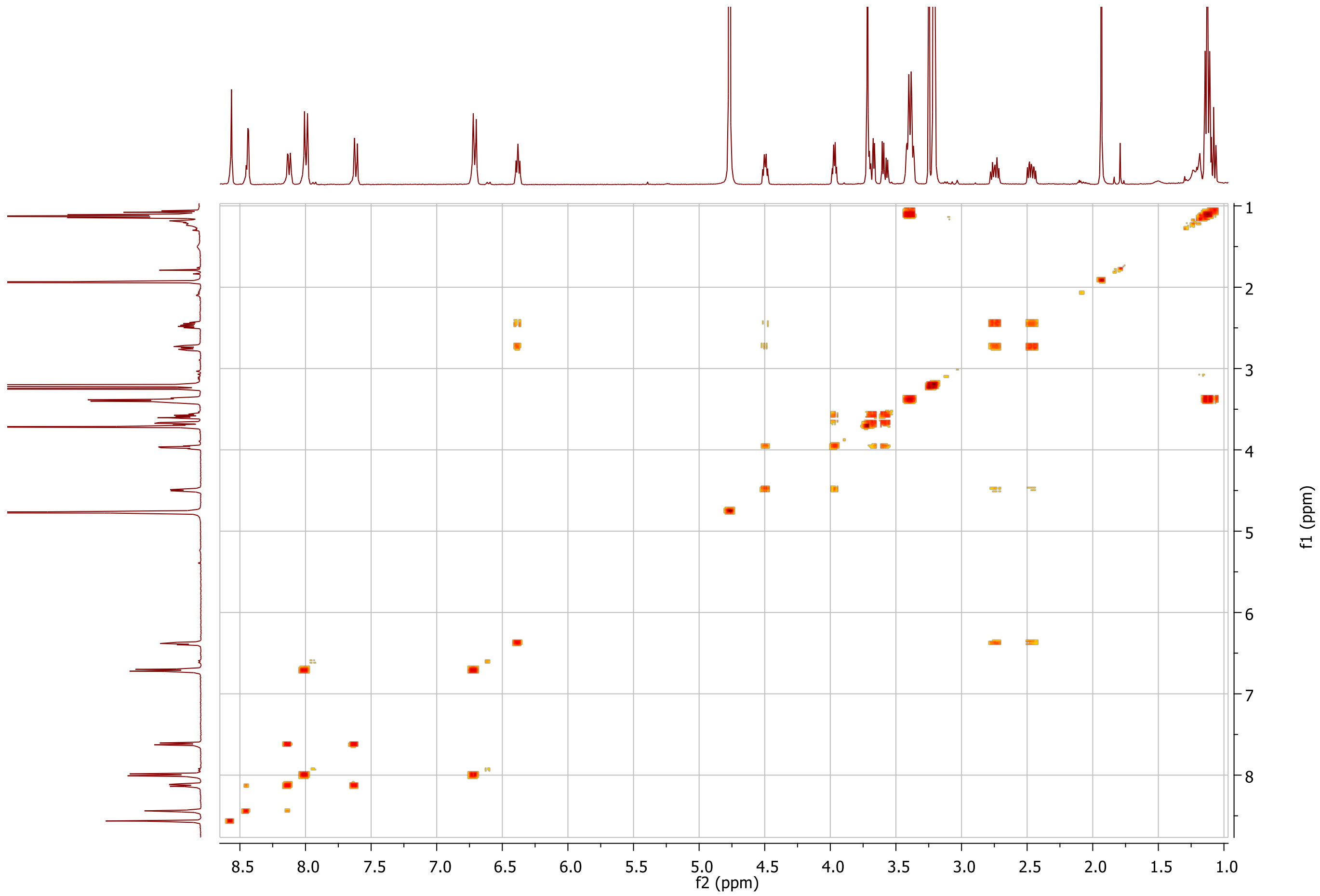
8.5 8.0 7.5 7.0 6.5 6.0 5.5 5.0 4.5 4.0 3.5 3.0 2.5 2.0 1.5 1.0
f2 (ppm)
1 2 3 4 5 6 7 8
f1 (ppm)

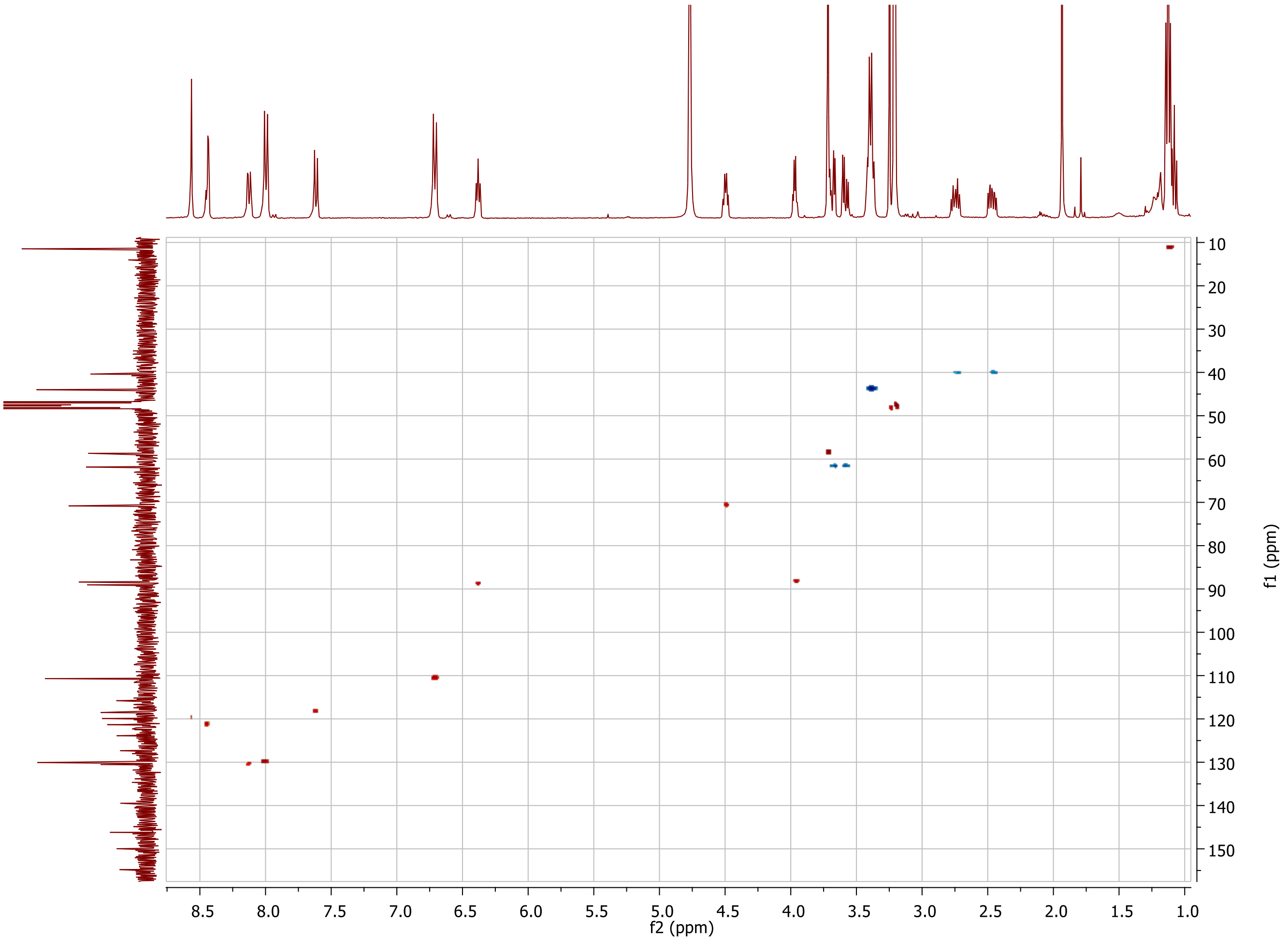
f2 (ppm)
f1 (ppm)

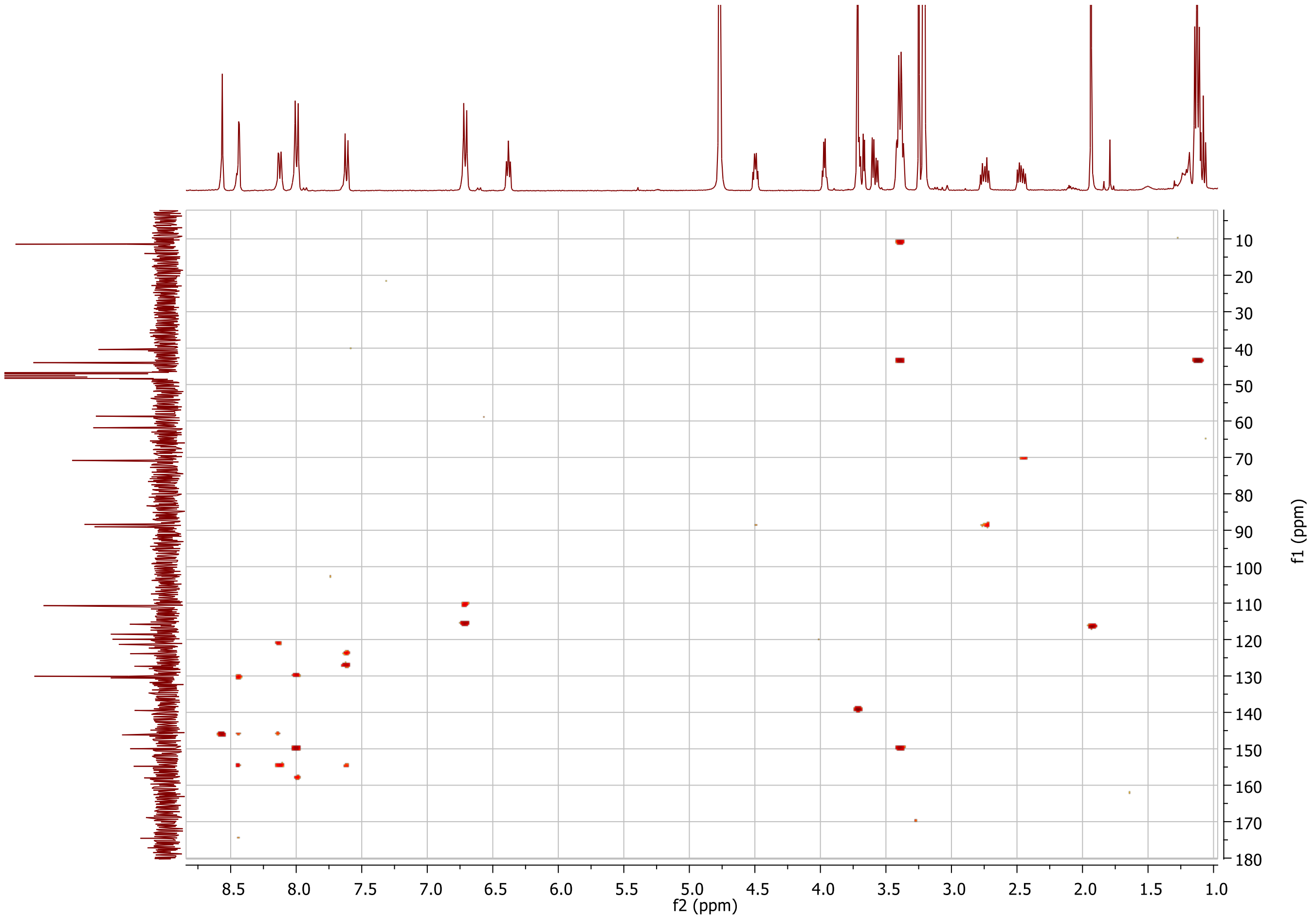
f2 (ppm)
f1 (ppm)
